# When UDG and hAPE1 Meet Cyclopurines. How (5′*R*) and (5′*S*) 5′,8-Cyclo-2′-deoxyadenosine and 5′,8-Cyclo-2′-deoxyguanosine Affect UDG and hAPE1 Activity?

**DOI:** 10.3390/molecules26175177

**Published:** 2021-08-26

**Authors:** Michał Szewczuk, Karolina Boguszewska, Julia Kaźmierczak-Barańska, Bolesław T. Karwowski

**Affiliations:** DNA Damage Laboratory of Food Science Department, Faculty of Pharmacy, Medical University of Lodz, ul. Muszynskiego 1, 90-151 Lodz, Poland; michal.szewczuk@umed.lodz.pl (M.S.); karolina.boguszewska@umed.lodz.pl (K.B.); julia.kazmierczak-baranska@umed.lodz.pl (J.K.-B.)

**Keywords:** 5′,8-cyclo-2′-deoxyadenosine (cdA), 5′,8-cyclo-2′-deoxyguanosine (cdG), tandem lesions, base excision repair, uracil-DNA glycosylase, human AP site endonuclease 1

## Abstract

Ionizing radiation is a factor that seriously damages cellular mechanisms/macromolecules, e.g., by inducing damage in the human genome, such as 5′,8-cyclo-2′-deoxypurines (cdPus). CdPus may become a component of clustered DNA lesions (CDL), which are notably unfavorable for the base excision repair system (BER). In this study, the influence of 5′*S* and 5′*R* diastereomers of 5′,8-cyclo-2′-deoxyadenosine (cdA) and 5′,8-cyclo-2′-deoxyguanosine (cdG) on the uracil-DNA glycosylase (UDG) and human AP site endonuclease 1 (hAPE1) activity has been taken under consideration. Synthetic oligonucleotides containing 2′-deoxyuridine (dU) and cdPu were used as a model of single-stranded CDL. The activity of the UDG and hAPE1 enzymes decreased in the presence of RcdG compared to ScdG. Contrary to the above, ScdA reduced enzyme activity more than RcdA. The presented results show the influence of cdPus lesions located within CDL on the activity of the initial stages of BER dependently on their position toward dU. Numerous studies have shown the biological importance of cdPus (e.g., as a risk of carcinogenesis). Due to that, it is important to understand how to recognize and eliminate this type of DNA damage from the genome.

## 1. Introduction

The stability of genetic information and the ability to reproduce is a key factor in cell survival. Many factors affect DNA and between 10,000 and a million DNA lesions are formed daily in every single cell in the human body [1,2].

Despite the presence of different repair systems, detection and/or removal is more difficult for some DNA damage. Therefore, mutations may be generated that lead to serious consequences, such as carcinogenicity.

Three main types of DNA lesions can be distinguished: isolated, tandem, and clustered DNA lesions (CDL). Tandem lesions occur as two contiguously damaged nucleotides generated by a single radical event, e.g., hydroxyl radical (•OH) and/or one-electron oxidants. Additionally, they may appear as more than one lesion within a single nucleotide with 5′,8-cyclo-2′-deoxypurines (cdPus) as an example [3,4,5]. CDL are referred to as at least two lesions per 1–2 DNA helix turns and are highly mutagenic [6]. Created mostly through the action of ionizing radiation, they are divided into double-stranded breaks (DSBs) and non-DSB clusters and may involve lesions located on the same or the opposing strands [3,7]. CDL containing cdPus are the substrates of nucleotide excision repair (NER) [8,9]. The cdPus form as a result of •OH action on the H5′-atom of the sugar moiety of 2′-deoxypurines [9,10,11,12]. 5′*S* and 5′*R* diastereomers of cdPus may change the spatial structure of ds-DNA through perturbing helix twist and base-pair stacking [9,10,13]. (5′*R*)−5′,8-cyclo-2′-deoxyadenosine (RcdA) and (5′*S*)−5′,8-cyclo-2′-deoxyadenosine (ScdA) are removed from DNA, respectively, 40 and 150 times slower than cis-platin adducts [14]. Studies indicate that when ds-DNA is exposed to ionizing radiation source in order to induce lesion appearance, 5′S diastereomer of both cdA and cdG predominate over 5′*R* [15]. Interestingly, 5′*R* diastereomers of cdA and 5′,8-cyclo-2′-deoxyguanosine (cdG) show higher affinity to NER machinery and are excised faster from the DNA than 5′*S* [9,16].

In general, isolated, oxidative lesions are mostly excised from the DNA by base excision repair (BER). However, BER may also be responsible for correcting lesions located within CDL [7]. Short patch (SP-BER) and long patch (LP-BER) mechanisms differ in the length of the repaired patch: one nucleotide for SP-BER and up to 12 nucleotides for LP-BER [17]. BER involves a group of specific enzymes, such as glycosylases recognizing damaged bases, endonucleases excising the damage, polymerases inserting correct nucleotides, and ligases rejoining the strands [18,19]. One of the most evolutionary conserved glycosylases is uracil-DNA glycosylase (UDG), which recognizes and excises 2′-deoxyuridine (dU) in single-stranded DNA (ss-DNA) and ds-DNA. Its action is followed by human AP site endonuclease 1 (hAPE1), which cleaves an abasic site (AP site) to form a single-strand break (SSB) that is subsequently recognized and processed by polymerases [18,20,21]. UDG is a monofunctional glycosylase widely used as a model enzyme for DNA repair research. It hydrolyzes the N-glycosidic bond between deoxyribose and nucleobase, thus, excising the base from the helix [20,22]. HAPE1 cleaves the phosphodiester backbone immediately 5′ to an AP site via a hydrolytic mechanism and generates a SSB with a 3′-hydroxyl and 5′-deoxyribose phosphate termini [23,24].

CdPus have become an attractive target in cancer diagnosis and a potential marker of treatment effectiveness [25,26]. For example, increased levels of cdPus were detected in tissues of inflammatory bowel disease (IBD) patients where level of lesions in inflamed tissues were approximately 20–40% higher than for non-inflamed tissues. Moreover, authors showed that 5′*R* diastereomer is probably repaired more efficiently than 5′*S* basing on the lower levels detected in examined samples [27]. The presence of cdPus in the genome may have many disadvantageous consequences [9]. As they are not repaired by BER system (the main pathway to correct oxidative DNA damage), they may accumulate and impair functioning of the cell [28]. When detected in the DNA helix, cdPus exert various proteins to bypass them, or if they cannot, their activity may be stopped completely. Studies show that polymerases are able to bypass chosen cdPus to some extent [29,30]. However, when these bulky structures block or impair molecular pathways, it may lead to the inhibition of gene expression, stopping replication, mutagenesis, or to development of neurological disorders, such as *Xeroderma Pigmentosum* [9]. Moreover, numerous studies indicate the unfavorable influence of cdPus lesions on the activity of repair enzymes, such as helicases FANCJ and RECQ1 [10,31]. However, the effect of cdPus’ presence within the CLD on the activity of BER enzymes and their action against second/third lesion is still obscure.

This study aimed to investigate the influence of 5′,8-cyclo-2′-deoxypurines ((5′*S*)−5′,8-cyclo-2′-deoxyadenosine (ScdA), (5′*R*)−5′,8-cyclo-2′-deoxyadenosine (RcdA), (5′*S*)−5′,8-cyclo-2′-deoxyguanosine (ScdG), or (5′*R*)−5′,8-cyclo-2′-deoxyguanosine (RcdG)), being a part of the single-stranded clustered DNA lesions, on the activity of UDG and hAPE1 that removes coexisting lesions (dU). The main focus was to examine the two initial steps of BER (lesion recognition and strand incision) in order to evaluate how cdPus affect the initiation of basic molecular mechanism of DNA damage repair. Substrate oligonucleotides contained lesions separated by five bases in both directions, as closer interlesion distances were previously studied [31,32]. Additionally, both diastereomers of cdG were taken into consideration in this context for the first time.

## 2. Results and Discussion

**Figure 1:**
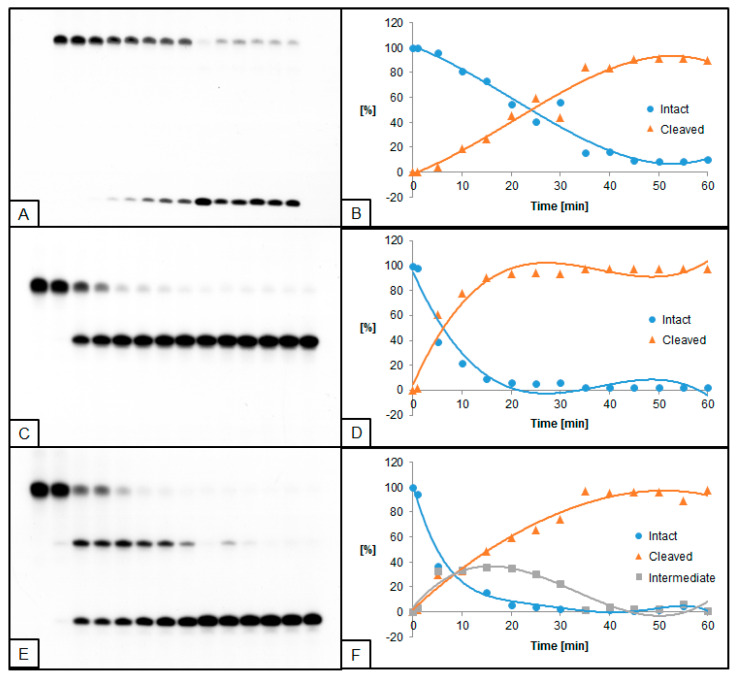
Cleavage of dsDNA containing dU and (5′*S*)−5′,8-cyclo-2′-deoxyadenosine (ScdA) by 0.02 U UDG and 0.5 U hAPE1. (**A**,**B**) dU(−5)ScdA; (**C**,**D**) dU(+5)ScdA; (**E**,**F**) dU(−5)(+5)ScdA. (**A**,**C**,**E**) show 14 lanes each, which correspond to reaction times 0, 1, 5, 10, 15, 20, 25, 30, 35, 40, 45, 50, 55, and 60 min starting from the left. (**B**,**D**,**F**) show the quantity losses of intact ssDNA (blue), the quantity increases of SSB-DNA (orange), and an intermediate oligo fragment (grey).

**Figure 2:**
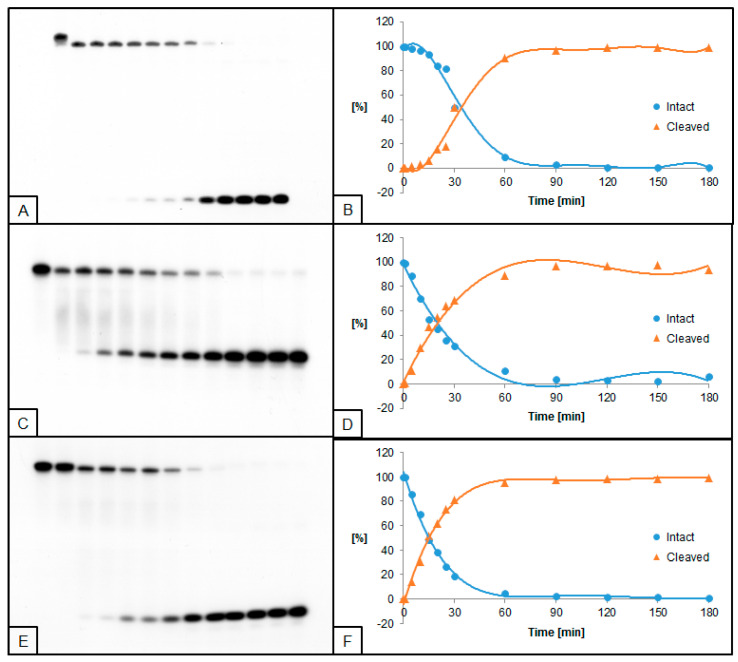
Cleavage of dsDNA containing dU and (5′*S*)−5′,8-cyclo-2′-deoxyadenosine (ScdA) by 0.5 U UDG and 0.02 U hAPE1. (**A**,**B**) dU(−5)ScdA; (**C**,**D**) dU(+5)ScdA; (**E**,**F**) dU(−5)(+5)ScdA. (**A**,**C**,**E**) show 13 lanes each, which correspond to reaction times 0, 1, 5, 10, 15, 20, 25, 30, 60, 90, 120, 150, and 180 min starting from the left. (**B**,**D**,**F**) show the quantity losses of intact ssDNA (blue) and the quantity increases of SSB-DNA (orange).

**Figure 3:**
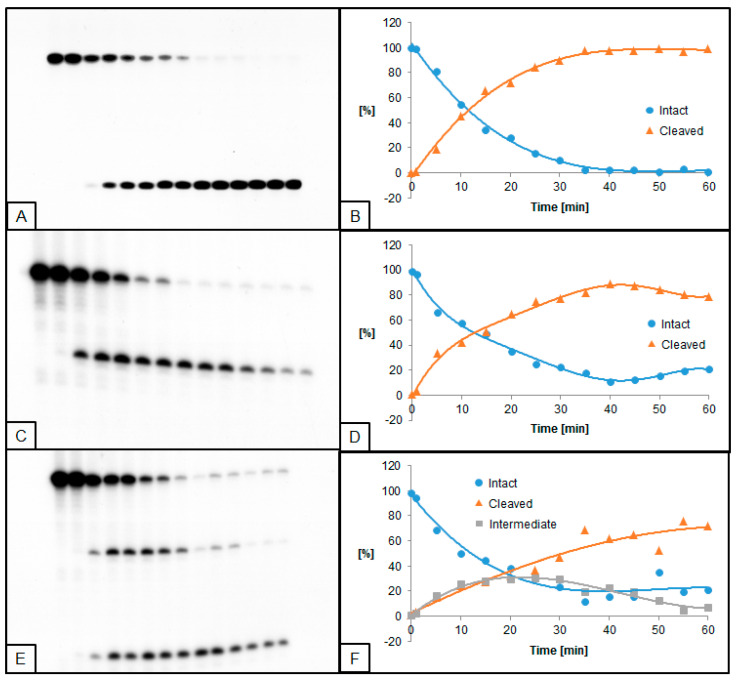
Cleavage of dsDNA containing dU and (5′*R*)−5′,8-cyclo-2′-deoxyadenosine (RcdA) by 0.02 U UDG and 0.5 U hAPE1. (**A**,**B**) dU(−5)RcdA; (**C**,**D**) dU(+5)RcdA; (**E**,**F**) dU(−5)(+5)RcdA. (**A**,**C**,**E**) show 14 lanes each, which correspond to reaction times 0, 1, 5, 10, 15, 20, 25, 30, 35, 40, 45, 50, 55, and 60 min starting from the left. (**B**,**D**,**F**) show the quantity losses of intact ssDNA (blue), the quantity increases of SSB-DNA (orange), and an intermediate oligo fragment (grey).

**Figure 4:**
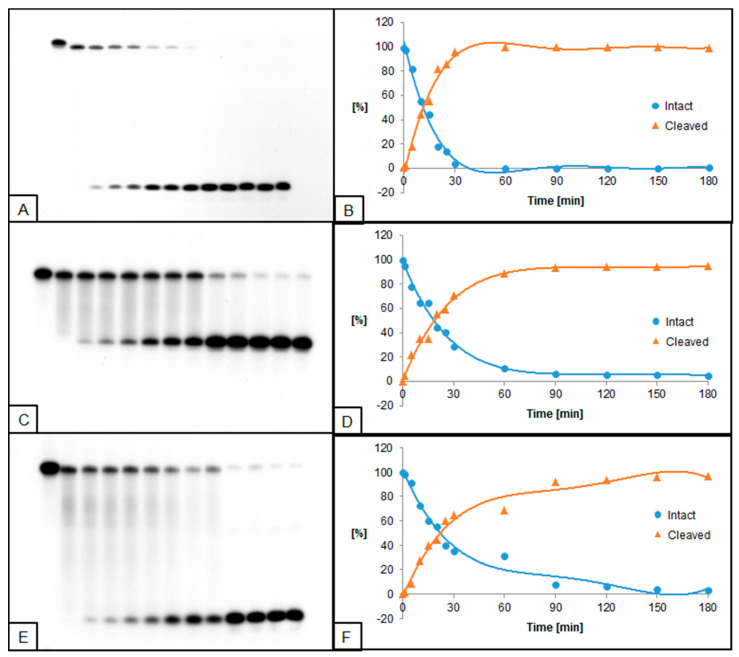
Cleavage of dsDNA containing dU and (5′*R*)−5′,8-cyclo-2′-deoxyadenosine (RcdA) by 0.5 U UDG and 0.02 U hAPE1. (**A**,**B**) dU(−5)RcdA; (**C**,**D**) dU(+5)RcdA; (**E**,**F**) dU(−5)(+5)RcdA. (**A**,**C**,**E**) show 13 lanes each, which correspond to reaction times 0, 1, 5, 10, 15, 20, 25, 30, 60, 90, 120, 150, and 180 min starting from the left. (**B**,**D**,**F**) show the quantity losses of intact ssDNA (blue) and the quantity increases of SSB-DNA (orange).

**Figure 5:**
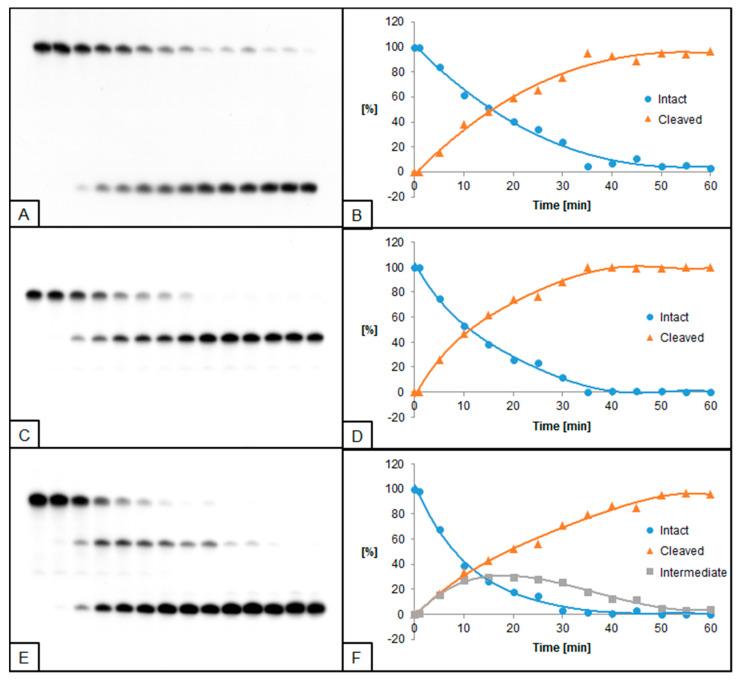
Cleavage of dsDNA containing dU and (5′*S*)−5′,8-cyclo-2′-deoxyguanosine (ScdG) by 0.02 U UDG and 0.5 U hAPE1. (**A**,**B**) dU(−5)ScdG; (**C**,**D**) dU(+5)ScdG; (**E**,**F**) dU(−5)(+5)ScdG. (**A**,**C**,**E**) show 14 lanes each, which correspond to reaction times 0, 1, 5, 10, 15, 20, 25, 30, 35, 40, 45, 50, 55, and 60 min starting from the left. (**B**,**D**,**F**) show the quantity loss of intact ssDNA (blue), the quantity increases of SSB-DNA (orange), and an intermediate oligo fragment (grey).

**Figure 6:**
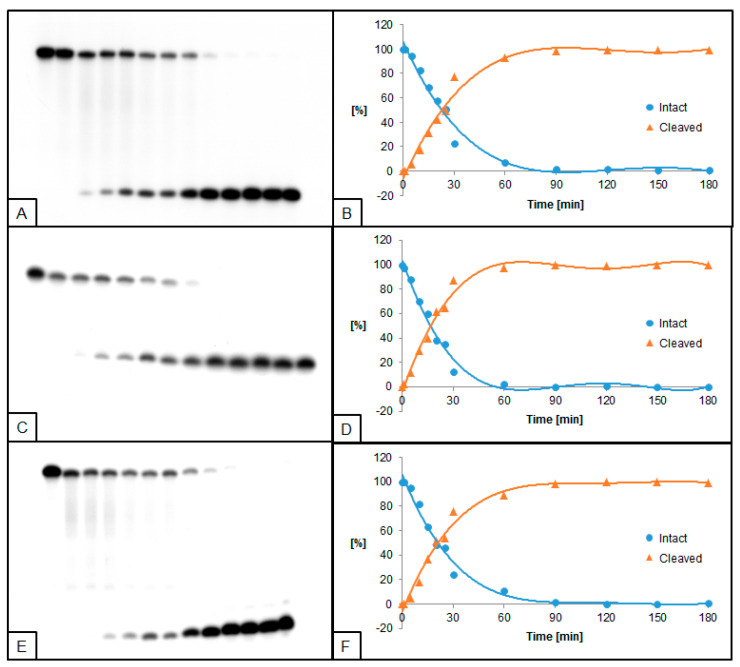
Cleavage of dsDNA containing dU and (5′*S*)−5′,8-cyclo-2′-deoxyguanosine (ScdG) by 0.5 U UDG and 0.02 U hAPE1. (**A**,**B**) dU(−5)ScdG; (**C**,**D**) dU(+5)ScdG; (**E**,**F**) dU(−5)(+5)ScdG. (**A**,**C**,**E**) show 13 lanes each, which correspond to reaction times 0, 1, 5, 10, 15, 20, 25, 30, 60, 90, 120, 150, and 180 min starting from the left. (**B**,**D**,**F**) show the quantity losses of intact ssDNA (blue) and the quantity increases of SSB-DNA (orange).

**Figure 7:**
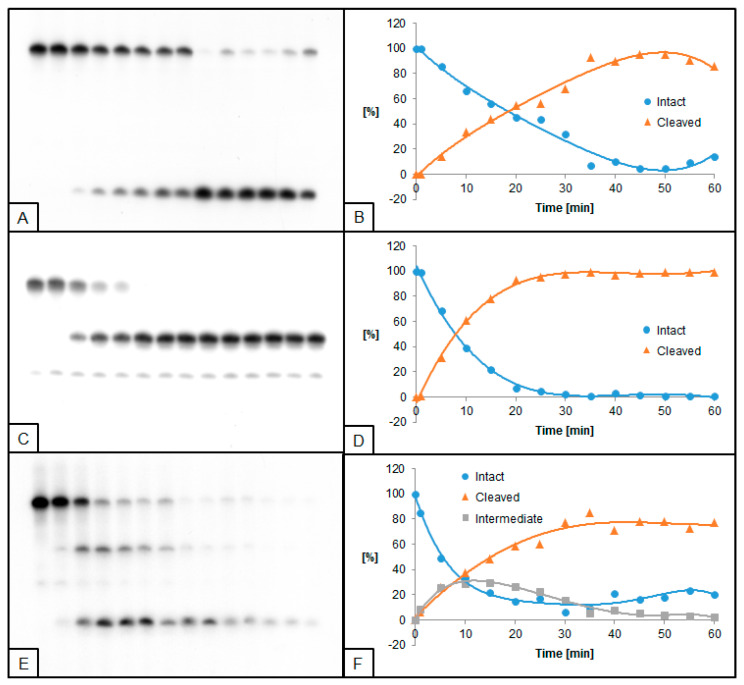
Cleavage of dsDNA containing dU and (5′*R*)−5′,8-cyclo-2′-deoxyguanosine (RcdG) by 0.02 U UDG and 0.5 U hAPE1. (**A**,**B**) dU(−5)RcdG; (**C**,**D**) dU(+5)RcdG; (**E**,**F**) dU(−5)(+5)RcdG. (**A**,**C**,**E**) show 14 lanes each, which correspond to reaction times 0, 1, 5, 10, 15, 20, 25, 30, 35, 40, 45, 50, 55, and 60 min starting from the left. (**B**,**D**,**F**) show the quantity loss of intact ssDNA (blue), the quantity increases of SSB-DNA (orange), and an intermediate oligo fragment (grey).

**Figure 8:**
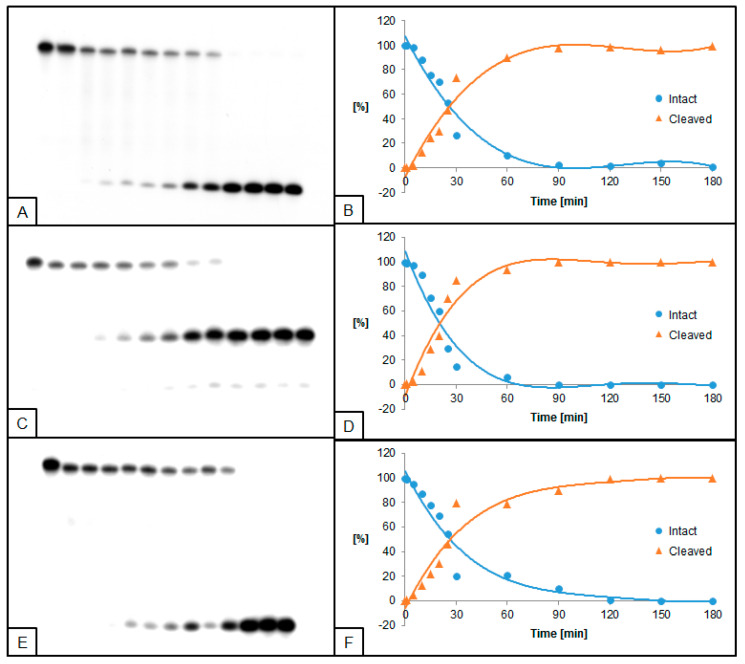
Cleavage of dsDNA containing dU and (5′*R*)−5′,8-cyclo-2′-deoxyguanosine (RcdG) by 0.5 U UDG and 0.02 U hAPE1. (**A**,**B**) dU(−5)RcdG; (**C**,**D**) dU(+5)RcdG; (**E**,**F**) dU(−5)(+5)RcdG. (**A**,**C**,**E**) show 13 lanes each, which correspond to reaction times 0, 1, 5, 10, 15, 20, 25, 30, 60, 90, 120, 150, and 180 min starting from the left. (**B**,**D**,**F**) show the quantity loss of intact ssDNA (blue) and the quantity increase of SSB-DNA (orange).

In the presented study, the activities of UDG and hAPE1 in the context of single-stranded CDL have been examined. UDG and hAPE1 are enzymes catalyzing the first two steps of the BER system—excision of recognized damage (AP site formation) and AP site hydrolysis (SSB formation), respectively. A set of radiolabeled, double-stranded oligonucleotides containing chosen cdPus, and one or two dU (see Table 1, Materials and Methods) was treated with UDG and/or hAPE1 (see Materials and Methods). As a model lesion, dU was chosen due to its well-established properties as a substrate for UDG and an AP site precursor. The obtained results were visualized by autoradiography (Figures 1–8). The strand cleavage sites were observed as one or two additional bands on an X-ray film, which represent the different oligonucleotide lengths: 40-mer (intact strand), 28-mer (cleaved strand for dU(+5) or intermediate strand for dU(−5)(+5)), and 18-mer (cleaved strand for dU(−5)) (Figure S89).

To examine the excision rate of dU, a mixture of both enzymes was used. For UDG activity assay, hAPE1 concentration was higher (0.5 U hAPE1 and 0.02 U UDG). As a result of using hAPE1 in excess, the AP sites were cleaved immediately to form SSB, which allowed easier visualization of results. In the case of hAPE1 activity testing, UDG concentration was higher (0.5 U UDG and 0.02 U hAPE1). Since hAPE1 cannot create AP sites, the excess of UDG immediately forms AP sites, and subsequent hAPE1 action (SSB formation) may be detected. The immediate formation of AP sites is visible on autoradiograms as a difference in bands placement between lanes corresponding to 0 and 1 min of reaction time (e.g., Figures 2A and 4A). Since cdPus are not substrates for the BER system, two oligonucleotides containing one or two dU lesions were selected as controls—dU0 and dU(−5)(+5)dA (Figure S7).

To ensure no additional enzymatic interactions between ds-DNA and tested enzymes, control assays for all oligonucleotides were performed (see Section 3 and Figure S89) and results were visualized using autoradiography (Figures S5 and S6). UDG and hAPE1 were used separately in the highest concentration (0.5 U). For the above-mentioned assays, no additional bands were denoted, showing that neither UDG nor hAPE1 were able to perform complete SSB formation when used separately.

### 2.1. UDG and hAPE1 Action towards 2′-Deoxyuridine

The efficiency of control oligonucleotide (dU0 and dU(−5)(+5)dA) degradation by UDG and hAPE1 was examined as the reference for further experiments.

A single dU showed 50% of excision from oligo dU0 after 20 min and full excision after 35 min (Figure S7). The presence of a second dU (dU(−5)(+5)dA, Table 1) did not affect this process (Figure S7). Although both incisions appeared after 5 min, creating 28-mer and 18-mer, the maximum strand cleavage was obtained also after 35 min.

The efficiency of hAPE1 action increased with two dUs in one strand (50% of SSB formation observed after 30 min and maximum strand cleavage after 90 min, Figure S7) comparing to single dU (50% of SSB formation observed after 60 min and maximum strand cleavage after 120 min, Figure S7).

The results for control oligos (dU0 and dU(−5)(+5)dA) are available in Supplementary Materials (Figure S7, Tables S2 and S3) and were used as reference points for further analyses.

### 2.2. The Influence of ScdA on UDG and hAPE1 Activity

Single dU located +5 bases to ScdA (dU(+5)ScdA, Figure 1C,D) was excised by UDG more effectively than in control (Figures S4 and S5). However, when dU was located in the opposite direction at the −5 position (dU(−5)ScdA) the decrease of UDG activity was noted (Figure 1A,B). Similar results were observed for the hAPE1 (Figure 2A–D, Figures S4 and S6). The comparative studies performed for CDL containing two dU moieties (dU(−5)(+5)dA vs. dU(−5)(+5)ScdA) elucidated that the activity of both enzymes increased for dU(−5)(+5)ScdA (Figure 1E,F and Figure 2E,F, Figures S7–S9). The UDG excised dU in approximately 50% after the similar time (10 min for dU(−5)(+5)dA vs. 15 min for dU(−5)(+5)ScdA), but total dU excision reached 90% after 50 min for dU(−5)(+5)dA and 97% after 35 min for dU(−5)(+5)ScdA. In the case of hAPE1 against dU(−5)(+5)ScdA, 50% of AP sites were hydrolyzed after 15 min, reaching the total strand cleavage of approximately 95% after 60 min (Figure 2F). It is compared to 30 and 90 min, respectively, which were needed to reach the same levels in the case of control dU(−5)(+5)dA oligo.

### 2.3. The Influence of RcdA on UDG and hAPE1 Activity

The influence of RcdA on the activity of UDG and hAPE1 was also evaluated. In general, UDG and hAPE1 activity reached higher levels for RcdA than for ScdA compared to control. Furthermore, for substrates with single dU lesions (dU(−5)RcdA and dU(+5)RcdA), the trend of excising dU was different than for ScdA—dU(−5)RcdA was excised prior to dU(+5)RcdA. For these oligos, 50% of dU excision was obtained after approximately 10 min, while the maximum of 97% was reached after 35 min for dU(−5)RcdA, compared to 89% after 40 min for dU(+5)RcdA. For dU located towards 5′-end, the time needed for the total dU excision by UDG decreased from 45 (Figure 1B) for ScdA to 35 min (Figure 3B) for RcdA. In the case of dU located towards 3′-end, the maximum dU excision was obtained after 40 min for both ScdA and RcdA (Figure 1D and Figure 3D). A similar effect was observed for hAPE1 activity. The 50% AP site cleavage was obtained faster for dU(−5)RcdA than dU(−5)ScdA (10 min vs. 30 min, respectively). However, for oligos with dU(+5), the difference was not detected. Additionally, in the case of dU(−5)(+5)RcdA, the UDG and hAPE1 activities were lower than dU(−5)(+5)ScdA (Figure 4E,F). At the same time, dU(−5)(+5)RcdA was the only case in which the activities of both enzymes were lower than in control (dU(−5)(+5)dA).

### 2.4. The Influence of ScdG on UDG and hAPE1 Activity

Based on previously obtained results concerning both cdA diastereomers, the influence of cdG on UDG and hAPE1 activity was taken under consideration. First, the ScdG was evaluated. For oligonucleotides containing a single dU (Figure 5A–D and Figure 6A–D), the activities of UDG and hAPE1 were similar to those obtained for RcdA (Figure 3A–D and Figure 4A–D). The dU located towards 3′-end was excised first, which was also denoted in the case of ScdA. Fifty percent of dU excision was obtained after 10 min for dU(+5)ScdG, compared to 15 min for dU(−5)ScdG. Moreover, the dU(−5)(+5)ScdG enzymatic cleavage elucidated similar results to dU(−5)(+5)ScdA but not to dU(−5)(+5)RcdA, which was particularly noticeable for UDG (Figure 5E,F). The total cleavage of the dU(−5)(+5)ScdG reached 95% after 50 min (UDG assay) and 99% after 90 min (hAPE1 assay) (Figure 5E,F and Figure 6E,F), compared to RcdA with 76% after 55 min and 96% after 150 min, respectively (Figure 3E,F and Figure 4E,F).

### 2.5. The Influence of RcdG on UDG and hAPE1 Activity

The presence of RcdG influenced the activity of UDG and hAPE1 differently than RcdA (Figure 3 and Figure 4). Moreover, the activity of both enzymes towards a single dU was lower for RcdG than ScdG. The only exception was dU(+5)RcdG—maximum dU excision by UDG was obtained after 30 min vs. 35 min for ScdG. Fifty percent cleavage of dU(−5)ScdG and dU(+5)ScdG by UDG was observed after approximately 15 min and 10 min, respectively (Figure 5A–D), compared to 20 min and 10 min observed for RcdG (Figure 7A–D). The activity of hAPE1 also differed between diastereomers for oligos with single dU. On the one hand, 50% of AP site cleavage was obtained after comparable time, and maximum cleavage values were similar between ScdG and RcdG. On the other hand, the AP site was cleaved more efficiently for dU(−5)ScdG and dU(+5)ScdG in the range from 5 to 20 min. For example, after 20 min, 42% and 62% of the AP site was cleaved for dU(−5)ScdG and dU(+5)ScdG, respectively (Figure 5, Figure 6, Figure 7 and Figure 8, Tables S2 and S3). For dU(−5)RcdG and dU(+5)RcdG, these values were 30% and 40%, respectively (Figure 7 and Figure 8, Tables S2 and S3). From 25 min onwards, values were comparable for both diastereomers. Surprisingly, this was in opposition to the results obtained for cdA, where the activity of both enzymes was higher for RcdA (Figure 3 and Figure 4) than for ScdA (Figure 1 and Figure 2). Despite that, the trend observed for ScdA and ScdG of excising dU located towards 3′-end prior to 5′-end was still maintained. The excision of 3′-directed dU by UDG from the strands containing two dU began after 5 min for both ScdG and RcdG. However, complete excision of 5′-directed dU was obtained after 60 min for ScdG (Figure 6E,F), but not for RcdG (Figure 8E,F).

This study continues the research concerning the influence of cdPus on the initiation of BER repair pathway, where the impact of both diastereomers of cdA on the excision of dU moieties by UDG and hAPE1 was shown [32]. To date, both diastereomers of cdG have not been investigated due to the challenging process of their synthesis and problems with their incorporation into DNA fragments [33].

First, the major observation is that UDG and hAPE1 activities increased in the presence of cdPus (compared with corresponding control). Furthermore, enzyme activity was lower for RcdG and ScdA than for ScdG and RcdA. These results are not entirely consistent with recent ones, where the influence of cdPus on complex DNA repair was investigated in nuclear extracts of eukaryotic cells [34]. It was concluded that incision of AP site-containing ds-DNA by endonucleases is more efficient when ScdA and ScdG are considered as a part of clustered lesions, in comparison to RcdA and RcdG. Also, the lesion repair was more efficient for 5′*S* diastereomer of both cdPus than for 5′*R* diastereomer [34].

Second, in the case of oligonucleotides containing single dU lesion, the activity of hAPE1 was affected to a greater extent (increased up to approximately 82% vs. control) than UDG activity (increased up to approximately 66% vs. control). In the case of dU lesions located towards 3′-end, the activity of UDG increased slower than hAPE1. For oligos containing dU located towards 5′-end, UDG activity was elevated only in the presence of RcdA and ScdG, while hAPE1 activity increased for all tested diastereomers. This may suggest that the presence of cdPus affects the mechanism of AP site recognition by hAPE1 more than it affects preceding UDG action.

Finally, it is assumed that the mechanism of recognition of both diastereomers of cdA and cdG by NER system might be different due to possible stereospecificity of enzymes involved [6]. It may also concern process of BER induction. Different helix distortion may lead to different mechanism of dU recognition, dependent on the type of cdPus located nearby. It is known that 5′*R* diastereomer of both cdA and cdG causes greater DNA duplex distortion and destabilization than 5′*S* diastereomer. This leads to higher efficiency of NER against 5′*R* than 5′*S* isomers [16]. Moreover, studies concerning HeLa cell extracts show that NER efficiency varies when ScdA and ScdG are paired with different complementary bases [33]. It may indicate that the efficiency of CDL repair depends on many factors and, to some extent, may give grounds for the results obtained in this study. It is worth noting that for oligonucleotides containing combination of two uracil moieties and cdPus, the increase in both UDG and hAPE1 activity was observed to a lesser extent comparing to oligonucleotides with single dU. UDG activity increased for oligos containing RcdG, while hAPE1 activity increased for oligos containing ScdA and ScdG. In other cases, no notable changes or decrease in enzyme activity were denoted. This may have occurred due to simultaneous recognition of both incision sites by protein molecules. Thus, DNA helix may be less prone to loosening within two locations at the same time and/or it may be more complicated for two enzyme molecules to operate in close proximity to each other. To unveil if these presumptions are correct, advanced research concerning both UDG and hAPE1 kinetics and mechanisms of lesion recognition are needed.

## 3. Materials and Methods

### 3.1. Substrate Oligonucleotides Synthesis and Purification

The oligonucleotides were synthesized and purified in the Bioorganic Chemistry Department, Polish Academy of Science, Lodz, Poland, using a Geneworld synthesizer (K & A Laborgeraete GbR, Schaafheim, Germany) and nucleotide phosphoroamidites (ChemGenes Corporation, Wilmington, MA, USA). The phosphoroamidite derivatives of (5′*R*)/(5′*S*) cdA and cdG were synthesized as described previously by Romieu et al. [35]. The crude oligonucleotides were purified by HPLC using Varian analytics with UV detection at wavelengths λ = 260 nm and Phenomenex C-18 column (Synergi 4 µm Fusion-RP 80Å, 250 × 4.6 mm) [36]. The complete sequences of substrate oligonucleotides are listed in Table 1. Nomenclature follows the rule that if dU is located towards the 3′-end of the strand in relation to the cdA/cdG, the number is positive. Otherwise, the numbers are negative. To give an example, oligo denoted as dU(−5)ScdA indicates that the dU was located five bases towards the 5′-end of the strand in relation to the ScdA, while in dU(+5)ScdA, dU was located five bases towards the 3′-end. The melting temperatures of oligonucleotides containing cdPus exceeded 70 °C, providing their stability in experimental conditions [32].

### 3.2. Oligonucleotides Concentration

The concentration of the obtained oligonucleotides was determined from a maximum absorbance ~260 nm using Varian Cary 1.3 E spectrophotometer (Varian, Brunn am Gebirge, Austria). The obtained quantities of analyzed oligonucleotides are presented in Table 2. The online oligonucleotide properties calculator OligoCalc [37] was used for the extinction coefficient determination of the oligonucleotides.

### 3.3. Mass Spectroscopy of Oligonucleotides

Oligonucleotides were analyzed in the negative-ion mode on a Waters Synapt G2-Si HDMS quadrupole time of flight hybrid mass spectrometer (Waters, Manchester, UK). Samples were dissolved in 10 mM ammonium acetate with 50% acetonitrile to obtain a final concentration of 0.1 OD/mL. Samples were injected into the source of the mass spectrometer by a syringe pump with a flow rate of 10 μL/min. Other parameters of the analysis were as follows: capillary voltage, 2.6 kV; cone voltage, 40 V; source temperature, 120 °C; desolvation temperature, 400 °C; cone gas, 30 L/h; and desolvation gas, 600 L/h. The data were collected in full-scan negative-ion mode (mass range of 50–2000 *m*/*z*) and the data processing was performed with Waters MassLynx 4.1 software (deconvolution with MaxEnt1 function, Waters Corporation, Milfors, MA, USA) [36]. The obtained and found masses of analyzed oligonucleotides are presented in Table 3. Mass spectra are available in Supplementary Materials (Figures S46–S60).

### 3.4. Preparation of 5′-End-Labeled Oligonucleotides

The 40-mer single-stranded oligonucleotides (230 pmol) were 5′-end-labeled using 5U of T4 polynucleotide kinase (New England BioLabs, Ipswich, MA, USA) with 2 μCi (0.2 μL) [γ-^32^P]ATP (Hartmann Analytic GmbH, Braunschweig, Germany) in 20 μL of buffer (pH 7.6 at 25 °C, 70 mM Tris-HCl, 10 mM MgCl_2_, 5 mM DTT). The reactions were performed for 30 min at 37 °C. The protein denaturation was obtained by heating the samples to 95 °C for 5 min [36]. Radiograms showing the efficiency of oligo labeling are available in the Supplementary Materials (Figures S1–S4).

### 3.5. Oligonucleotide Hybridization

The radiolabeled oligonucleotides were hybridized with a 2-fold excess of the purified non-radiolabeled complementary strand in pure H_2_O. Complementary strands were selected as follows: matrix SA for dU0, dU(−5)ScdA, and dU(−5)RcdA; matrix SA-A for dU(−5)(+5)dA, dU(+5)ScdA, dU(−5)(+5)ScdA, dU(+5)RcdA, and dU(−5)(+5)RcdA; matrix SG for dU(−5)ScdG and dU(−5)RcdG; matrix SG-A for dU(+5)ScdG, dU(−5)(+5)ScdG, dU(+5)RcdG, and dU(−5)(+5)RcdG.

After 10 min in 90 °C, samples were cooled down to room temperature over 3–4 h to ensure proper annealing of complementary strands. Obtained double-stranded DNA (dsDNA) fragments were precipitated with 250 μL of ice-cold ethanol for 30 min and centrifuged with 13,000 rpm at 4 °C for 30 min. Ethanol was removed, and samples were dried under reduced pressure at room temperature. The efficiency of the hybridization process and the purity of both single- and double-stranded radiolabeled oligonucleotides were examined by PAGE electrophoresis on 15% denaturing polyacrylamide gel containing 8 M urea in 1 × TBE (89 mM Tris-HCl, 89 mM boric acid, 2 mM EDTA) for 120 min at a constant power of 45 W [36]. Radiograms showing the efficiency of oligonucleotides hybridization are available in the Supplementary Materials (Figures S1–S4).

### 3.6. UDG and hAPE1 Cleavage Assay

UDG and hAPE1 were purchased from NEB (New England BioLabs, Ipswich, MA, USA). The general procedure of the UDG and hAPE1 cleavage assay of each oligonucleotide was as follows. The radiolabeled oligonucleotides (2.3 pmol) were incubated in 5 µL of reaction buffer (50 mM potassium acetate, 20 mM Tris-acetate, 10 mM magnesium acetate, and 1 mM DTT, pH = 7.9) with UDG and hAPE1 at 37 °C for each reaction time. For UDG cleavage assay, 0.02 U of UDG and 0.5 U of hAPE1 were used for 0, 1, 5, 10, 15, 20, 25, 30, 35, 40, 45, 50, 55, and 60 min. For hAPE1 cleavage assay, 0.5 U of UDG and 0.02 U of hAPE1 were used for 0, 1, 5, 10, 15, 20, 25, 30, 60, 90, 120, 150, and 180 min. Moreover, control assays for both enzymes were performed using the procedure as follows. The radiolabeled oligonucleotides (2.3 pmol) were incubated in 5 µL of reaction buffer (50 mM potassium acetate, 20 mM Tris-acetate, 10 mM magnesium acetate, and 1 mM DTT, pH = 7.9) with UDG or hAPE1 at 37 °C for each reaction time. For the UDG control assay, 0.5 U of UDG was used for 0 and 60 min. For the hAPE1 control assay, 0.5 U hAPE1 was used for 0 and 180 min.

The reactions were stopped by cooling down the samples in an ice/water bath and addition of 5 µL of denaturing loading dye (95% formamide, 2 mM EDTA, 0.025% bromophenol blue, 0.025% xylene cyanol). The efficiency of the cleavage was determined on 15% or 20% denaturing polyacrylamide gel containing 8 M urea in 1 × TBE (89 mM Tris-HCl, 89 mM boric acid, 2 mM EDTA) for 120 min at a constant power of 45 W. The results of PAGE electrophoresis were visualized by autoradiography. Each set of data was quantified using Quantity One 1-D analysis software (Bio-Rad, Hercules, CA, USA). All experiments were performed three times to ensure that repetitive and consistent data are provided. To obtain a percentage value of DNA cleavage, the intensity of each band was calculated as a percentage of the total intensity of all bands within one lane.

## 4. Conclusions

DNA damage can affect genome stability and may lead to serious molecular problems, such as mutagenesis. CdPus appear in the genome as a specific type of DNA tandem lesions that could be a part of CDL. Numerous studies indicate the biological significance of CDL and cdPus [28,36,38,39]. Since cdPus are mainly excised by the NER system, little is known about their influence on BER. Recent studies indicate that the presence of cdPus within CDL slows down DNA repair, especially when a short distance between lesions is considered [32,40]. In this article, the influence of cdPus-containing CDL on dU excision by initial BER enzymes has been evaluated.

Results of this study are summarized in Table 4 and may lead to the following conclusions:

- For all substrate oligos, AP sites generated by UDG were suitable for subsequent hAPE1 action. This suggests that cdPus do not affect the structure of the AP site created after dU excision by UDG.
- dU present within CDL is excised more efficiently when located towards the 3′-end of cdPus (except for oligonucleotides containing RcdA and single dU lesion).
- The presence of ScdA within CDL (dU(−5)ScdA, dU(+5)ScdA and dU(−5)(+5)ScdA) forces the negative effect on dU excision by UDG in comparison with RcdA, however, the opposite trend for ScdG and RcdG was denoted.
- The cleavage of dU located in the +5 position within CDL containing two dU was higher for dU(−5)(+5)ScdA and dU(−5)(+5)RcdG than for dU(−5)(+5)RcdA and dU(−5)(+5)ScdG.
- The activity of hAPE1 increased for the majority of cdPus (except for dU(−5)(+5)RcdA and dU(−5)(+5) RcdG).
- The activity of UDG increased or did not change for the majority of cdPus, especially for oligonucleotides containing a single dU located towards 3′-end (except for dU(−5)ScdA and dU(−5)(+5)RcdA where enzyme activity was lower than the control).
- For oligos with a single dU (dU(−5) and dU(+5)), the hAPE1 activity increased for all substrates; the UDG activity was also elevated with the exception of dU(−5)ScdA and dU(−5)RcdG.
- The observed increase in enzymatic activities was higher for hAPE1 than for UDG.

UDG and hAPE1 activity mostly increased towards cdPus-containing CDL (compared to control). That may indicate a significant impact of cdPus on lesion recognition by BER enzymes. It is relevant to answer the question of how cdPus increase the activity of initial BER steps. Little is known about protein recruitment to the lesion proximity and mechanism of lesion recognition. The results presented above indicate that cdG increases the activity of examined enzymes or leaves them unaffected. In the case of cdA, the enzymatic activities differed depending on the mutual distribution of lesions within the CDL. Perhaps it is related to the ability to form two hydrogen bonds by adenine, which makes the system more flexible. The presence of guanine with three hydrogen bonds stabilizes the DNA helix, which is subsequently more readable for BER repair enzymes. Therefore, further studies concerning the spatial structure of CDL and crystallographic research are necessary. It is assumed that five base pairs distance between dU and cdPus prevents inhibition of dU excision. At the same time, changes in the DNA structure provided by cdPus may affect protein recruitment and lesion recognition.

It is known that any impact on BER machinery may lead to its slowdown and, thereby, cause mutagenesis. Since cancer has become a challenging task for modern medicine, researchers are investigating the field of DNA damage. The results presented in this study, concerning the effects of specific DNA lesions on repair enzymes, could be useful in cancer drug research and lead to therapeutic applications.

## Figures and Tables

**Table 1:** The sequence of substrate oligonucleotides containing 2′-deoxyuridine (dU) and 5′,8-cyclo-2′-deoxypurines.

	End	1	2	3	4	5	6	7	8	9	10	11	12	13	14	15	16	17	18	19	20	21	22	23	24	25	26	27	28	29	30	31	32	33	34	35	36	37	38	39	40
Matrix SA	3′	G	A	G	A	A	C	A	G	T	C	C	T	T	A	T	A	A	C	A	G	A	G	A	T	A	C	G	A	G	G	G	T	G	G	T	T	T	C	C	G
Matrix SA-A	3′	G	A	G	A	A	C	A	G	T	C	C	T	T	A	T	A	A	C	A	G	A	G	A	T	A	C	G	A	A	G	G	T	G	G	T	T	T	C	C	G
Matrix SG	3′	G	A	G	A	A	C	A	G	T	C	C	T	T	A	T	A	A	C	A	G	A	G	A	C	A	C	G	A	G	G	G	T	G	G	T	T	T	C	C	G
Matrix SG-A	3′	G	A	G	A	A	C	A	G	T	C	C	T	T	A	T	A	A	C	A	G	A	G	A	C	A	C	G	A	A	G	G	T	G	G	T	T	T	C	C	G
dU0	5′	C	T	C	T	T	G	T	C	A	G	G	A	A	T	A	T	T	G	T	C	**U**	C	T	A	T	G	C	T	C	C	C	A	C	C	A	A	A	G	G	C
dU(−5)(+5)dA	5′	C	T	C	T	T	G	T	C	A	G	G	A	A	T	A	T	T	G	**U**	C	T	C	T	A	T	G	C	T	**U**	C	C	A	C	C	A	A	A	G	G	C
dU(−5)ScdA	5′	C	T	C	T	T	G	T	C	A	G	G	A	A	T	A	T	T	G	**U**	C	T	C	T	**SX**	T	G	C	T	C	C	C	A	C	C	A	A	A	G	G	C
dU(+5)ScdA	5′	C	T	C	T	T	G	T	C	A	G	G	A	A	T	A	T	T	G	T	C	T	C	T	**SX**	T	G	C	T	**U**	C	C	A	C	C	A	A	A	G	G	C
dU(−5)(+5)ScdA	5′	C	T	C	T	T	G	T	C	A	G	G	A	A	T	A	T	T	G	**U**	C	T	C	T	**SX**	T	G	C	T	**U**	C	C	A	C	C	A	A	A	G	G	C
dU(−5)RcdA	5′	C	T	C	T	T	G	T	C	A	G	G	A	A	T	A	T	T	G	**U**	C	T	C	T	**RX**	T	G	C	T	C	C	C	A	C	C	A	A	A	G	G	C
dU(+5)RcdA	5′	C	T	C	T	T	G	T	C	A	G	G	A	A	T	A	T	T	G	T	C	T	C	T	**RX**	T	G	C	T	**U**	C	C	A	C	C	A	A	A	G	G	C
dU(−5)(+5)RcdA	5′	C	T	C	T	T	G	T	C	A	G	G	A	A	T	A	T	T	G	**U**	C	T	C	T	**RX**	T	G	C	T	**U**	C	C	A	C	C	A	A	A	G	G	C
dU(−5)ScdG	5′	C	T	C	T	T	G	T	C	A	G	G	A	A	T	A	T	T	G	**U**	C	T	C	T	**SY**	T	G	C	T	C	C	C	A	C	C	A	A	A	G	G	C
dU(+5)ScdG	5′	C	T	C	T	T	G	T	C	A	G	G	A	A	T	A	T	T	G	T	C	T	C	T	**SY**	T	G	C	T	**U**	C	C	A	C	C	A	A	A	G	G	C
dU(−5)(+5)ScdG	5′	C	T	C	T	T	G	T	C	A	G	G	A	A	T	A	T	T	G	**U**	C	T	C	T	**SY**	T	G	C	T	**U**	C	C	A	C	C	A	A	A	G	G	C
dU(−5)RcdG	5′	C	T	C	T	T	G	T	C	A	G	G	A	A	T	A	T	T	G	**U**	C	T	C	T	**RY**	T	G	C	T	C	C	C	A	C	C	A	A	A	G	G	C
dU(+5)RcdG	5′	C	T	C	T	T	G	T	C	A	G	G	A	A	T	A	T	T	G	T	C	T	C	T	**RY**	T	G	C	T	**U**	C	C	A	C	C	A	A	A	G	G	C
dU(−5)(+5)RcdG	5′	C	T	C	T	T	G	T	C	A	G	G	A	A	T	A	T	T	G	**U**	C	T	C	T	**RY**	T	G	C	T	**U**	C	C	A	C	C	A	A	A	G	G	C

Abbreviations mean the following: SX—(5′S)−5′,8-cyclo-2′-deoxyadenosine; RX—(5′R)−5′,8-cyclo-2′-deoxyadenosine; SY—(5′S)−5′,8-cyclo-2′-deoxyguanosine; RY—(5′R)−5′,8-cyclo-2′-deoxyguanosine; U—2′-deoxyuridine.

**Table 2:** The obtained quantities [OD] and [nmol] of oligonucleotides.

Oligonucleotide	Quantity [OD]	Quantity [nmol]
Matrix SA	27.2	62.56
Matrix SA-A	12.6	28.98
Matrix SG	30.0	69.00
Matrix SG-A	13.6	31.28
dU0	26.6	61.18
dU(−5)(+5)dA	28.2	64.86
dU(−5)ScdA	24.5	56.35
dU(+5)ScdA	22.6	51.98
dU(−5)(+5)ScdA	22.5	51.75
dU(−5)RcdA	22.0	50.60
dU(+5)RcdA	25.0	57.50
dU(−5)(+5)RcdA	26.2	60.26
dU(−5)ScdG	18.6	42.78
dU(+5)ScdG	11.0	25.30
dU(−5)(+5)ScdG	9.6	22.08
dU(−5)RcdG	2.0	4.60
dU(+5)RcdG	1.3	2.99
dU(−5)(+5)RcdG	2.2	5.06

**Table 3:** The calculated and found molecular masses [Da] of oligonucleotides.

Oligonucleotide	Calculated Mass	Found Mass
Matrix SA	12,409.00	12,409.82
dU0	12,158.83	12,168.25
dU(−5)(+5)dA	12,168.85	12,168.05
dU(−5)ScdA	12,166.84	12,166.50
dU(+5)ScdA	12,181.85	12,181.54
dU(−5)(+5)ScdA	12,167.85	12,167.20
dU(−5)RcdA	12,166.84	12,166.20
dU(+5)RcdA	12,181.85	12,181.10
dU(−5)(+5)RcdA	12,167.85	12,167.00
dU(−5)ScdG	12,182.84	12,182.10
dU(+5)ScdG	12,197.85	12,197.00
dU(−5)(+5)ScdG	12,183.85	12,182.90
dU(−5)RcdG	12,182.84	12,182.64
dU(+5)RcdG	12,197.85	12,195.75
dU(−5)(+5)RcdG	12,183.85	12,183.90

**Table 4:** The activity of UDG and hAPE1 towards dU within cdPus-containing CLD, in relation to controls: dU0 and dU(−5)(+5)dA.

cdPus	UDG Activity			hAPE1 Activity		
ScdA	-	+	ND	+	+	+
RcdA	+	+	-	+	+	-
ScdG	+	+	ND	+	+	+
RcdG	ND	+	+	+	+	ND
	−5	+5	−5/+5	−5	+5	−5/+5
	The relative position of dU towards cdPus within CDL					

Abbreviations mean the following: ND—the difference in enzyme activity not detected, “+”—increased enzyme activity in relation to control, “-”—decreased enzyme activity in relation to control.

## Data Availability

Not applicable.

## Supplementary Materials

Table S1: Complete sequences of applied oligonucleotides, Tables S2 and S3: Raw numerical data of densitometry, Figure S1: Efficiency of oligo labeling and hybridization, Figures S2 and S3: Visualization of control cleavage assay of oligonucleotides, Figures S4–S45: Autoradiograms and graphs of cleavage assays of oligonucleotides, Figures S46–S60: Mass spectra of applied oligonucleotides, Figures S61–S88: Cleavage assays of oligonucleotides: graphs including error bars, Figure S89: Schematic representation of cleavage assays and control cleavage assays.

## Sample Availability

Not available.
