# Supplementary material for: When UDG and hAPE1 Meet Cyclopurines. How (5′R) and (5′S) 5′,8-Cyclo-2′-deoxyadenosine and 5′,8-Cyclo-2′-deoxyguanosine Affect UDG and hAPE1 Activity?
Source: Molecules. 2021 Aug 26;26(17):5177. doi: 10.3390/molecules26175177 (PMC8434022; doi:10.3390/molecules26175177)
Supplement: Supplementary file 1 [file molecules-26-05177-s001.zip › molecules-1324002-supplementary.pdf]

When UDG and hAPE1 meet Cyclopurines. How (5'*R*) and (5'*S*) 5',8-cyclo-2'-deoxyadenosine and 5',8-cyclo-2'-deoxyguanosine affect UDG and hAPE1 activity?

Supplementary Materials

Table of contents

Page	Item	Description
2	Table S1	Complete sequences of applied oligonucleotides
3	Figure S1	Efficiency of oligo labeling and hybridization
4-5	Figure S2-S3	Visualization of control cleavage assay of oligonucleotides
6-33	Figure S4-S45	Autoradiograms and graphs of cleavage assays of oligonucleotides
34-48	Figure S46-S60	Mass spectra of applied oligonucleotides
49-62	Figure S61-S88	Cleavage assays of oligonucleotides: graphs including error bars
63-72	Table S2-S3	Raw numerical data of densitometry
73	Figure S89	Schematic representation of cleavage assays and control cleavage assays

Table S1. Complete sequences of applied oligonucleotides. Abbreviations mean the following: SX - (5'S)-5'8-cyclo-2'-deoxyadenosine; RX - (5'R)-5'8-cyclo-2'-deoxyadenosine; SY - (5'S)-5'8-cyclo-2'-deoxyguanosine; RY - (5'R)-5'8-cyclo-2'-deoxyguanosine; U – 2'-deoxyuridine.

Oligo	End	Sequence	End
Matrix SA	3'	GAGAACAGTCCTTATAACAGAGATACGAGGGTGGTTTCCG	5'
Matrix SA-A	3'	GAGAACAGTCCTTATAACAGAGATACGAAGGTGGTTTCCG	5'
Matrix SG	3'	GAGAACAGTCCTTATAACAGAGACACGAGGGTGGTTTCCG	5'
Matrix SG-A	3'	GAGAACAGTCCTTATAACAGAGACACGAAGGTGGTTTCCG	5'
dU0	5'	CTCTTGTCAGGAATATTGTC**U**CTATGCTCCCACCAAAGGC	3'
dU(-5)(+5)dA	5'	CTCTTGTCAGGAATATTG**U**CTCTATGCT**U**CCACCAAAGGC	3'
dU(-5)ScdA	5'	CTCTTGTCAGGAATATTG**U**CTCT**SX**TGCTCCCACCAAAGGC	3'
dU(+5)ScdA	5'	CTCTTGTCAGGAATATTGTCTCT**SX**TGCT**U**CCACCAAAGGC	3'
dU(-5)(+5)ScdA	5'	CTCTTGTCAGGAATATTG**U**CTCT**SX**TGCT**U**CCACCAAAGGC	3'
dU(-5)RcdA	5'	CTCTTGTCAGGAATATTG**U**CTCT**RX**TGCTCCCACCAAAGGC	3'
dU(+5)RcdA	5'	CTCTTGTCAGGAATATTGTCTCT**RX**TGCT**U**CCACCAAAGGC	3'
dU(-5)(+5)RcdA	5'	CTCTTGTCAGGAATATTG**U**CTCT**RX**TGCT**U**CCACCAAAGGC	3'
dU(-5)ScdG	5'	CTCTTGTCAGGAATATTGUCTCT**SY**TGCTCCCACCAAAGGC	3'
dU(+5)ScdG	5'	CTCTTGTCAGGAATATTGTCTCT**SY**TGCTUCCACCAAAGGC	3'
dU(-5)(+5)ScdG	5'	CTCTTGTCAGGAATATTGUCTCT**SY**TGCTUCCACCAAAGGC	3'
dU(-5)RcdG	5'	CTCTTGTCAGGAATATTGUCTCT**RY**TGCTCCCACCAAAGGC	3'
dU(+5)RcdG	5'	CTCTTGTCAGGAATATTGTCTCT**RY**TGCTUCCACCAAAGGC	3'
dU(-5)(+5)RcdG	5'	CTCTTGTCAGGAATATTGUCTCT**RY**TGCTUCCACCAAAGGC	3'

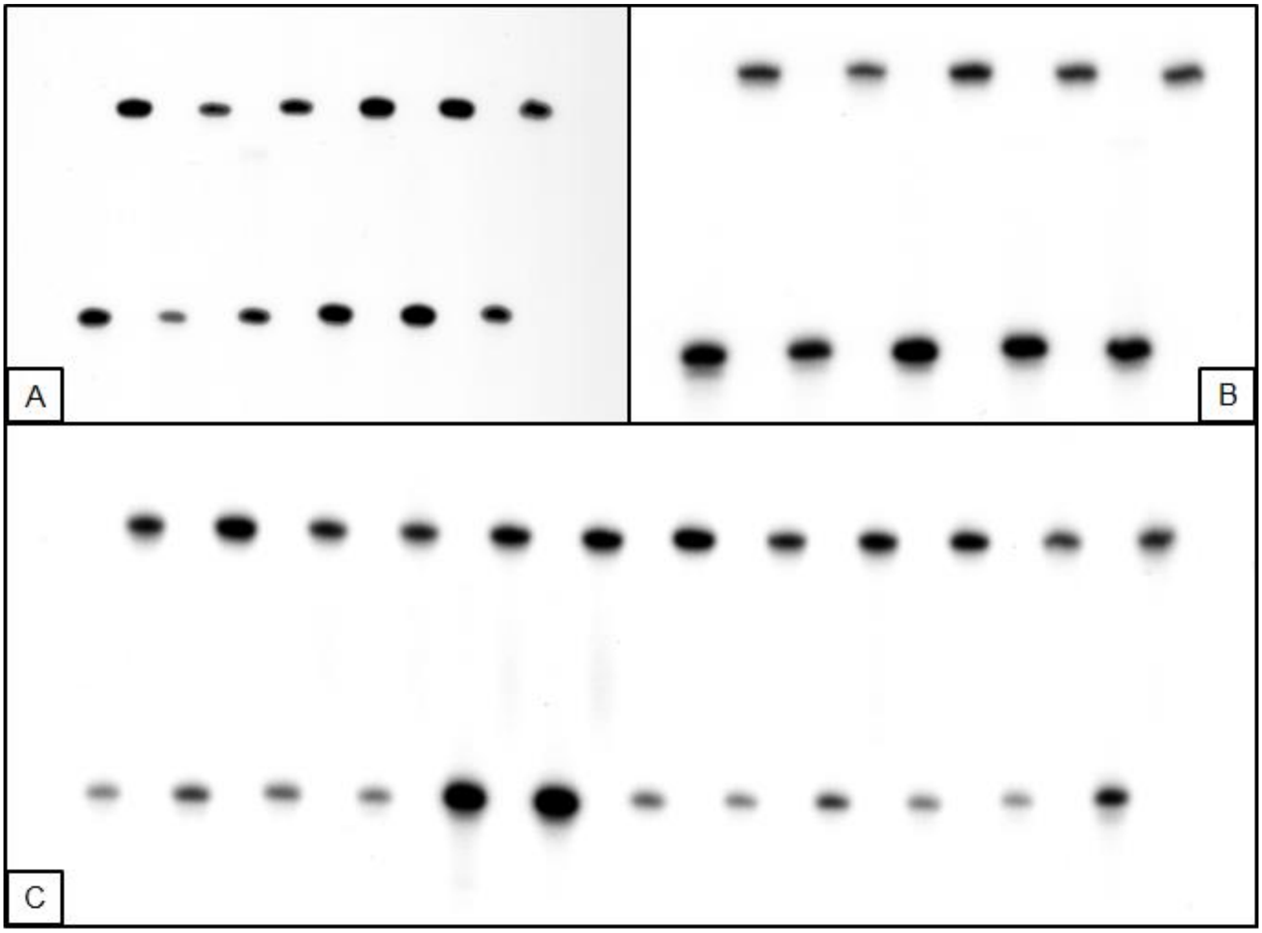

Figure S1. Visualization of efficient labeling (ssDNA) and hybridization (dsDNA) of oligonucleotides. Upper row of bands show 40-mer ds-DNA, lower row of bands show 40-mer ss-DNA. The order of tested oligonucleotides was as follows: dU(-5)ScdA (lanes 5,6), dU(-5)RcdA (lanes 9,10) (A); dU0 (lanes 1,2) (B); dU(-5)(+5)dA (lanes 3,4), dU(+5)ScdA (lanes 5,6), dU(-5)(+5)ScdA (lanes 7,8), dU(+5)RcdA (lanes 9,10), dU(-5)(+5)RcdA (lanes 11,12), dU(-5)ScdG (lanes 13,14), dU(+5)ScdG (lanes 15,16), dU(-5)(+5)ScdG (lanes 17,18), dU(-5)RcdG (lanes 19,20), dU(+5)RcdG (lanes 21,22), dU(-5)(+5)RcdG (23,24) (C).

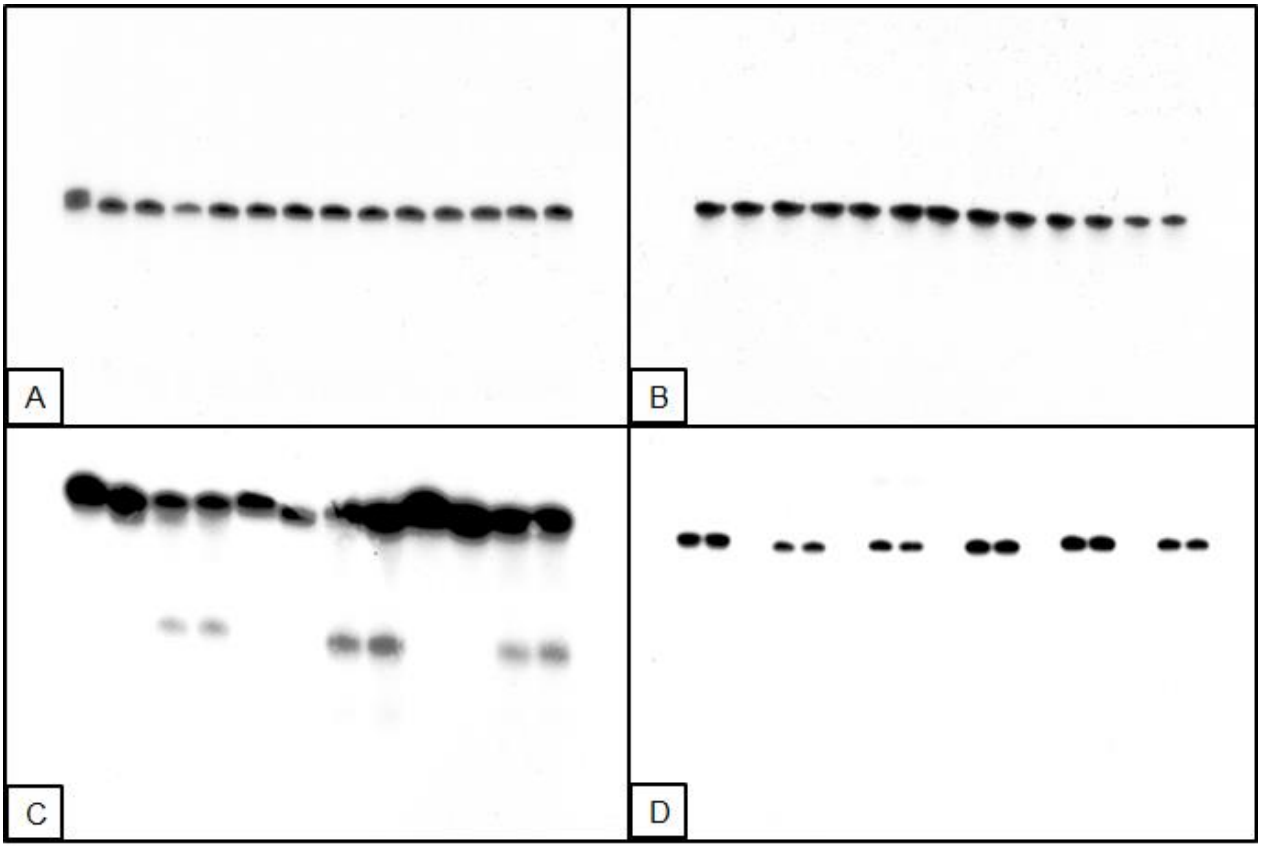

Figure S2. Visualization of control cleavage assays of oligonucleotides by 0.5U UDG (A,C) or 0.5U hAPE1 (B,C). Single row of bands indicate no cleavage of ds-DNA by the enzymes used separately. (A) cleavage of dU0 by 0.5U UDG, (B) cleavage of dU0 by 0.5U hAPE1. (A) show 14 lanes each, which correspond to reaction times 0, 1, 5, 10, 15, 20, 25, 30, 35, 40, 45, 50, 55, and 60 min starting from the left. (B) show 13 lanes each, which correspond to reaction times 0, 1, 5, 10, 15, 20, 25, 30, 60, 90, 120, 150 and 180 min starting from the left. (C) cleavage of dU(-5)ScdA (lane 5: 0 min; lane 6: 60 min) and dU(-5)RcdA (lane 9: 0 min; lane 10: 60 min) by 0.5U UDG. (D) cleavage of dU(-5)ScdA (lane 7: 0 min; lane 8: 60 min) and dU(-5)RcdA (lane 13: 0 min; lane 14: 60 min) by 0.5U hAPE1.

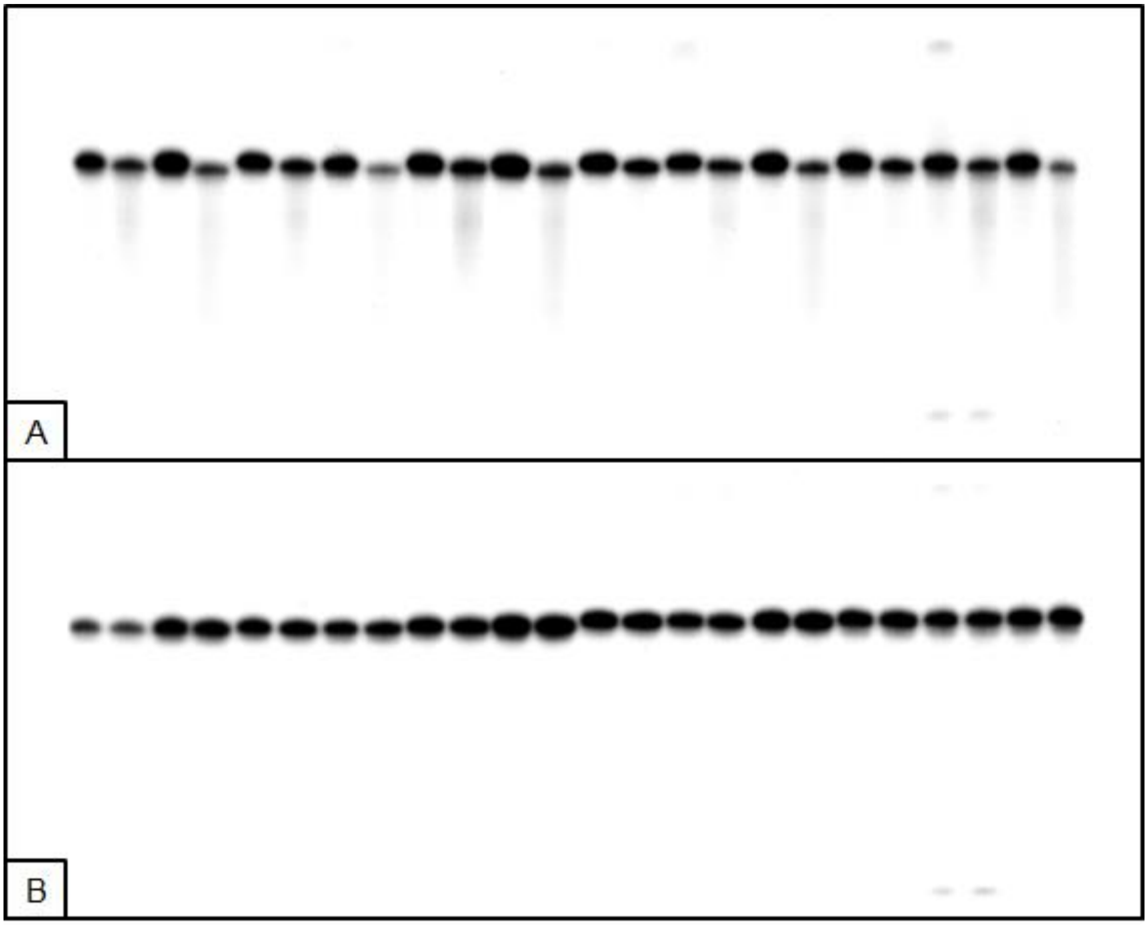

Figure S3. Visualization of control cleavage assays of oligonucleotides by 0.5U UDG (A) or 0.5U hAPE1 (B). Single row of bands indicate no cleavage of ds-DNA by the enzymes used separately. For each oligonucleotide the reaction times were 0 min (left lane of the pair) and 60 min (right lane of the pair) (A) or 0 min (left lane of the pair) and 180 min (right lane of the pair) (B). The order of tested oligonucleotides on both autoradiograms (A) and (B) was as follows: dU(-5)(+5)dA (lanes 3,4), dU(+5)ScdA (lanes 5,6), dU(-5)(+5)ScdA (lanes 7,8), dU(+5)RcdA (lanes 9,10), dU(-5)(+5)RcdA (lanes 11,12), dU(-5)ScdG (lanes 13,14), dU(+5)ScdG (lanes 15,16), dU(-5)(+5)ScdG (lanes 17,18), dU(-5)RcdG (lanes 19,20), dU(+5)RcdG (lanes 21,22), dU(-5)(+5)RcdG (23,24).

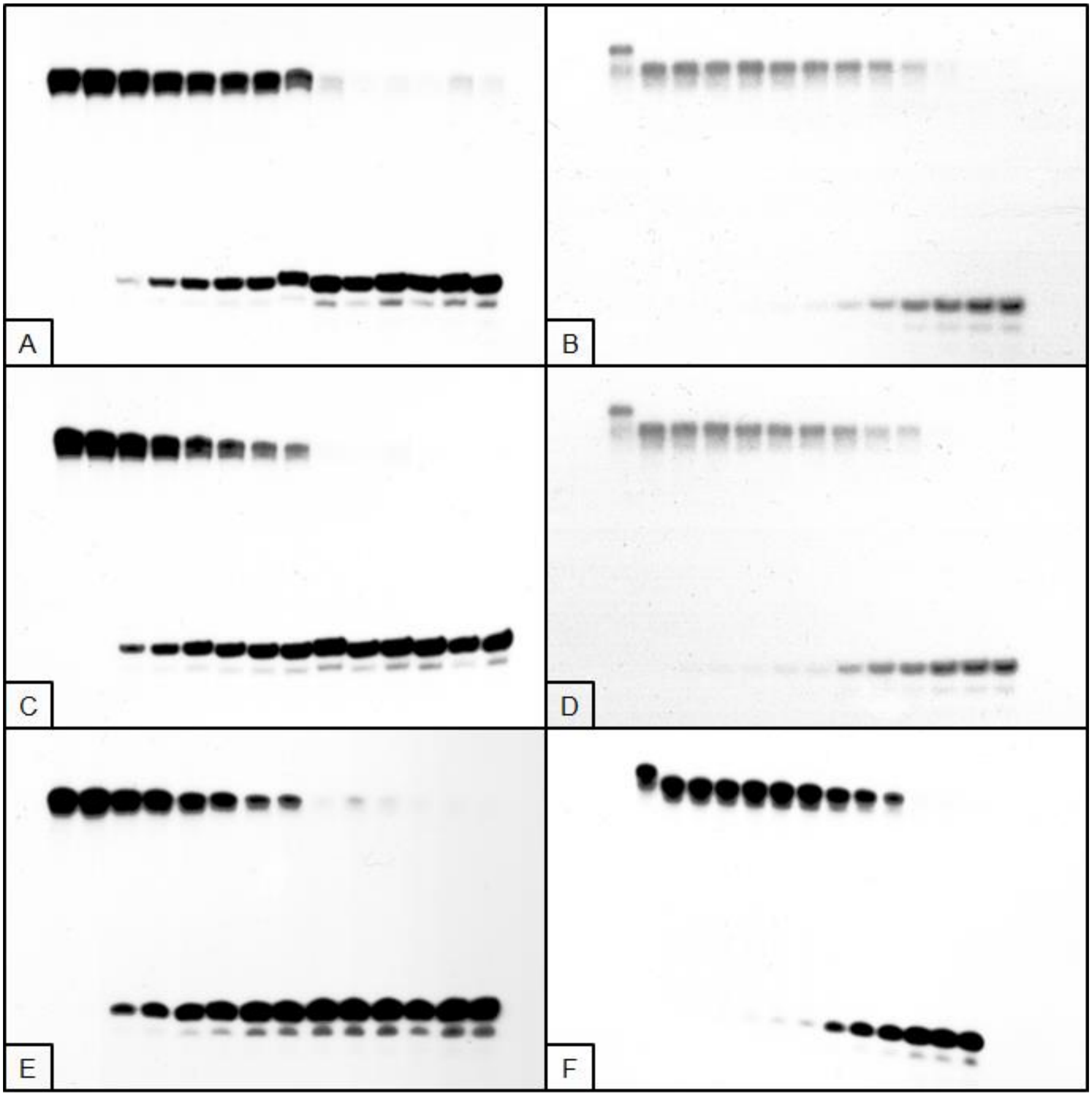

Figure S4. Cleavage assay of dU0 by 0.02U UDG and 0.5U hAPE1 (A,C,E) or 0.5U UDG and 0.02U hAPE1 (B,D,F). (A), (C) and (E) show 14 lanes each, which correspond to reaction times 0, 1, 5, 10, 15, 20, 25, 30, 35, 40, 45, 50, 55, and 60 min starting from the left. (B), (D) and (F) show 13 lanes each, which correspond to reaction times 0, 1, 5, 10, 15, 20, 25, 30, 60, 90, 120, 150 and 180 min starting from the left.

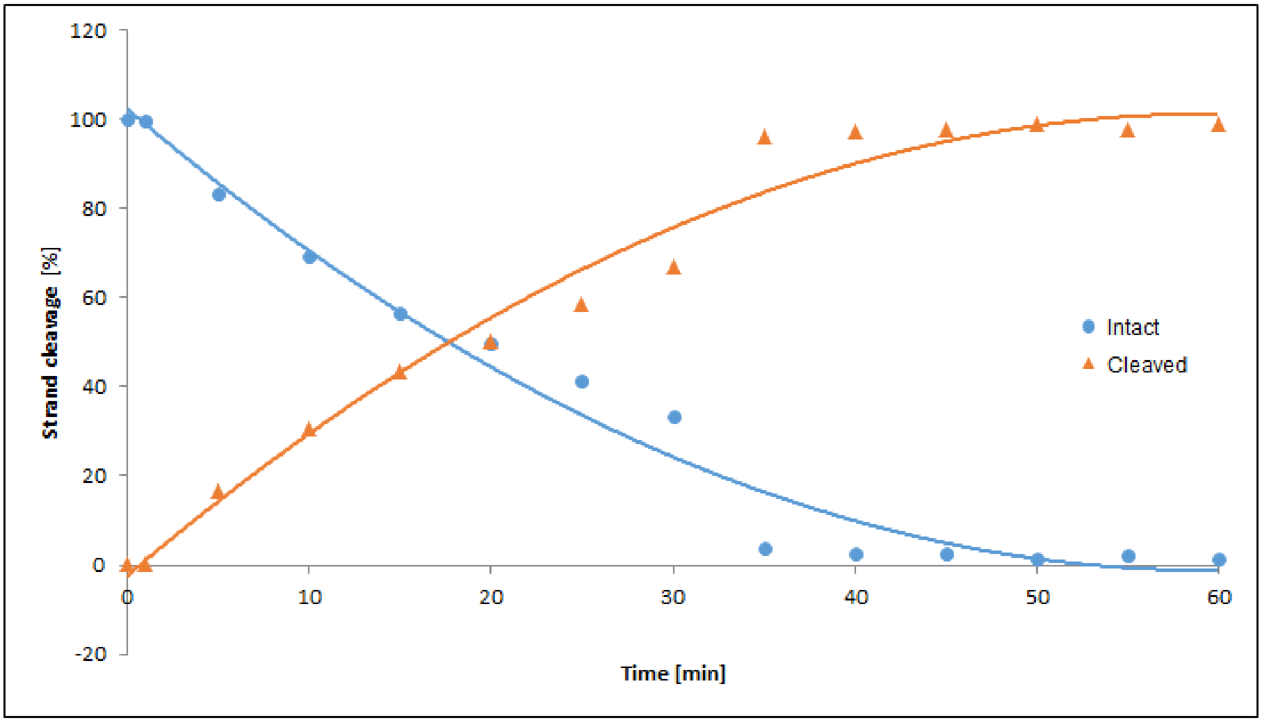

Figure S5. Cleavage assay of dU0 by 0.02U UDG and 0.5U hAPE1. The quantity loss of intact ssDNA (blue) and the quantity increase of SSB-DNA (orange).

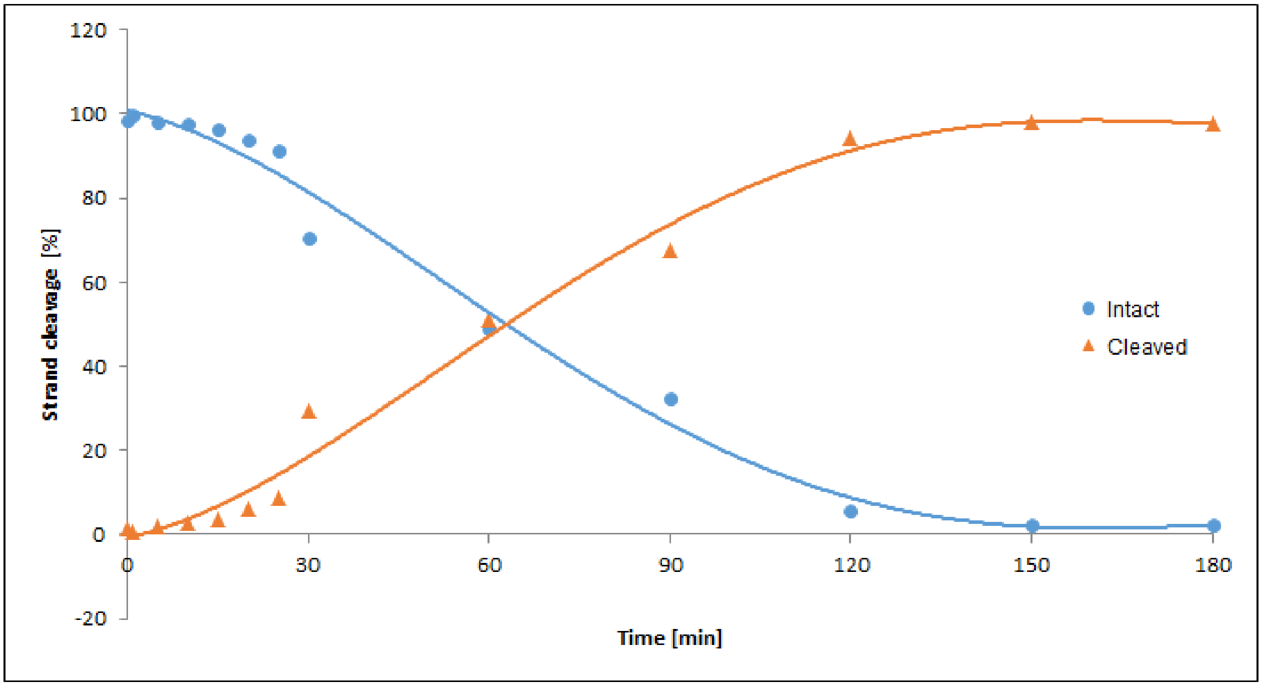

Figure S6. Cleavage assay of dU0 by 0.5U UDG and 0.02U hAPE1. The quantity loss of intact ssDNA (blue) and the quantity increase of SSB-DNA (orange).

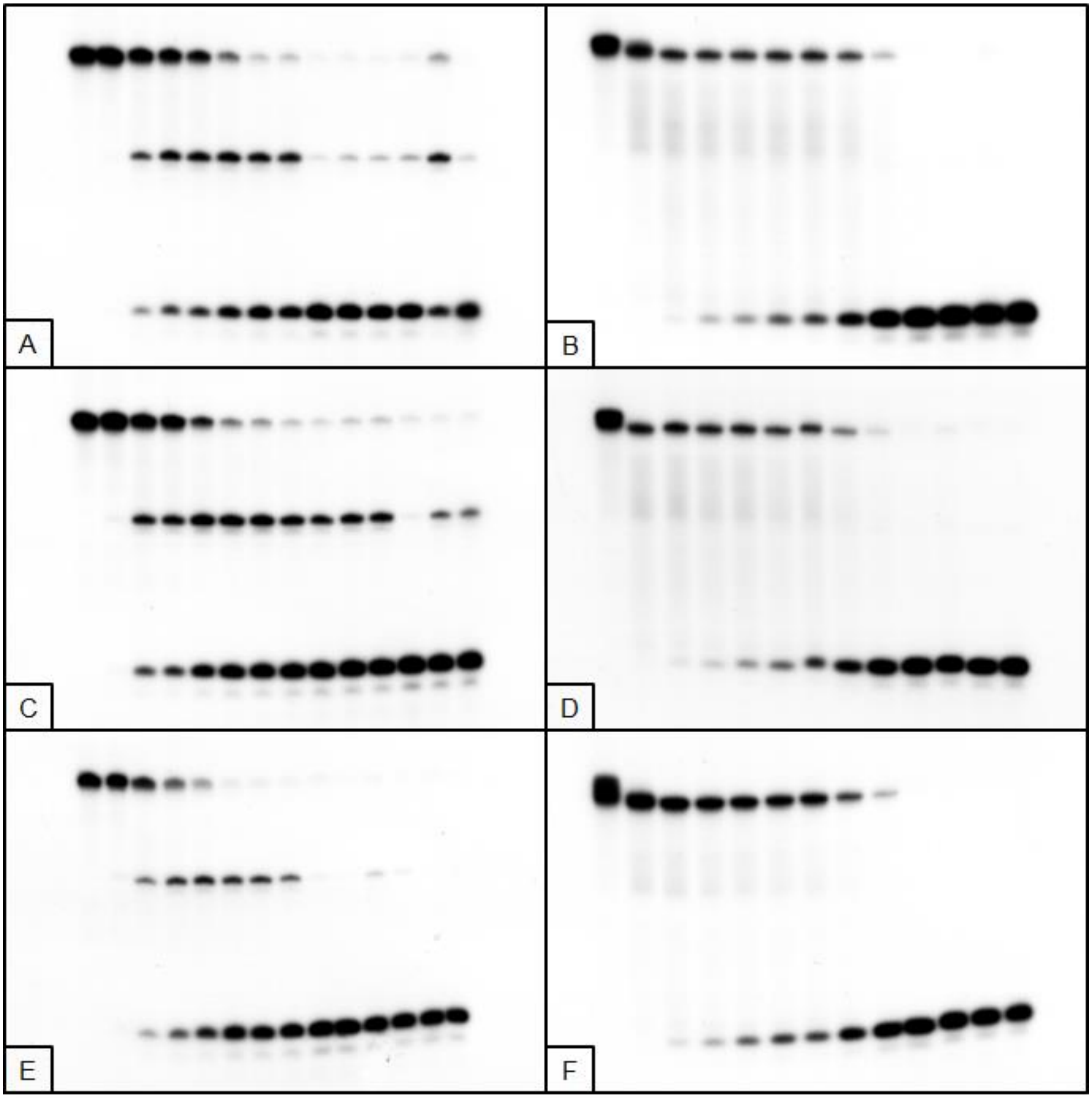

Figure S7. Cleavage assay of dU(-5)(+5)dA by 0.02U UDG and 0.5U hAPE1 (A,C,E) or 0.5U UDG and 0.02U hAPE1 (B,D,F). (A), (C) and (E) show 14 lanes each, which correspond to reaction times 0, 1, 5, 10, 15, 20, 25, 30, 35, 40, 45, 50, 55, and 60 min starting from the left. (B), (D) and (F) show 13 lanes each, which correspond to reaction times 0, 1, 5, 10, 15, 20, 25, 30, 60, 90, 120, 150 and 180 min starting from the left.

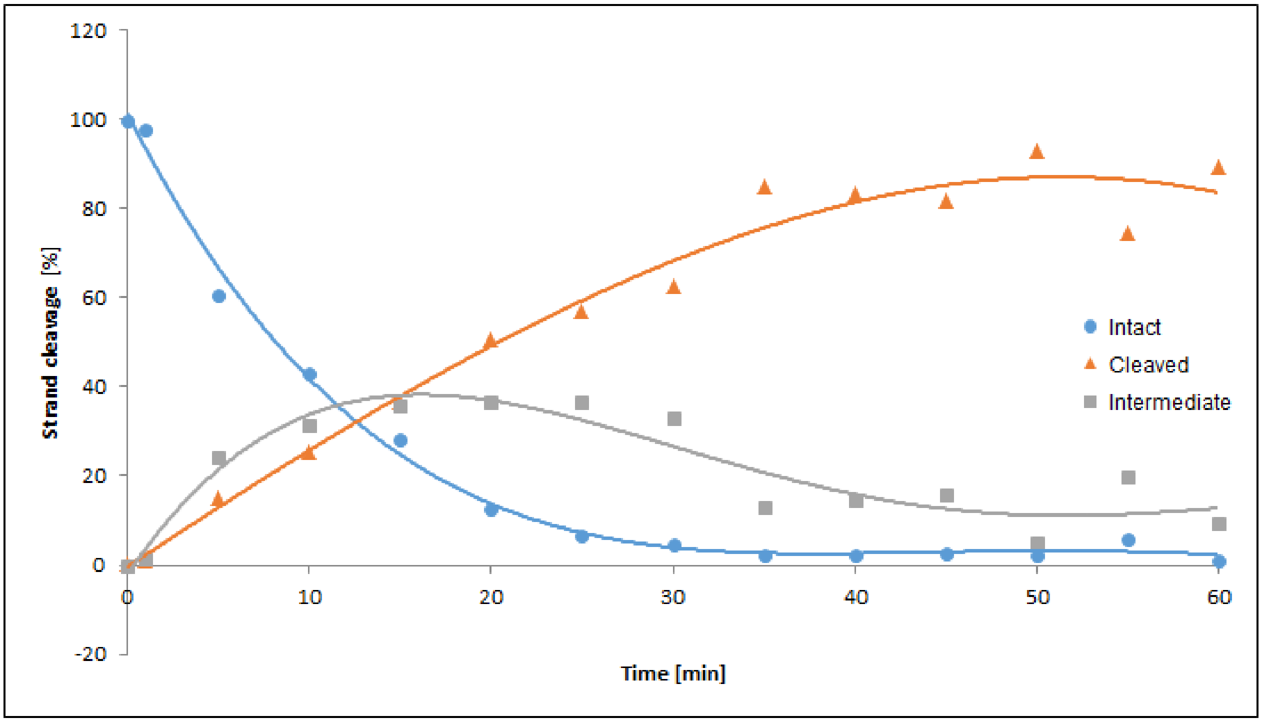

Figure S8. Cleavage assay of dU(-5)(+5)dA by 0.02U UDG and 0.5U hAPE1. The quantity loss of intact ssDNA (blue), the quantity increase of SSB-DNA (orange) and an intermediate oligo fragment (grey).

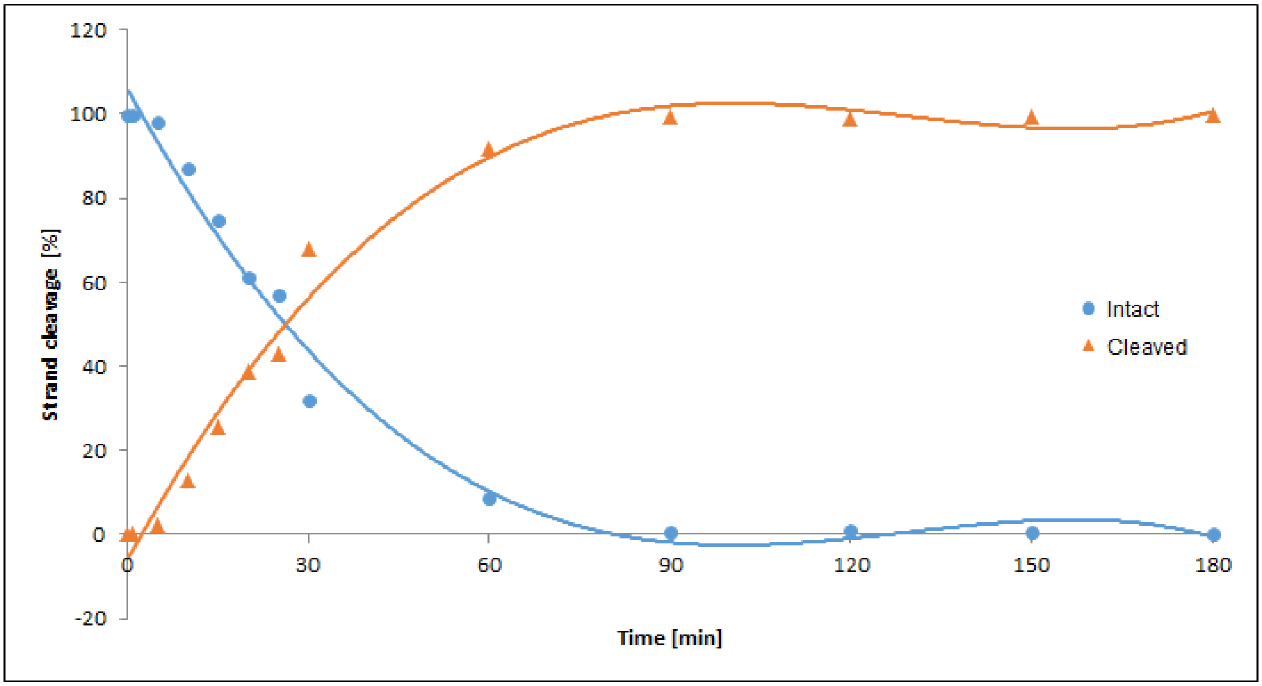

Figure S9. Cleavage assay of dU(-5)(+5)dA by 0.5U UDG and 0.02U hAPE1. The quantity loss of intact ssDNA (blue) and the quantity increase of SSB-DNA (orange).

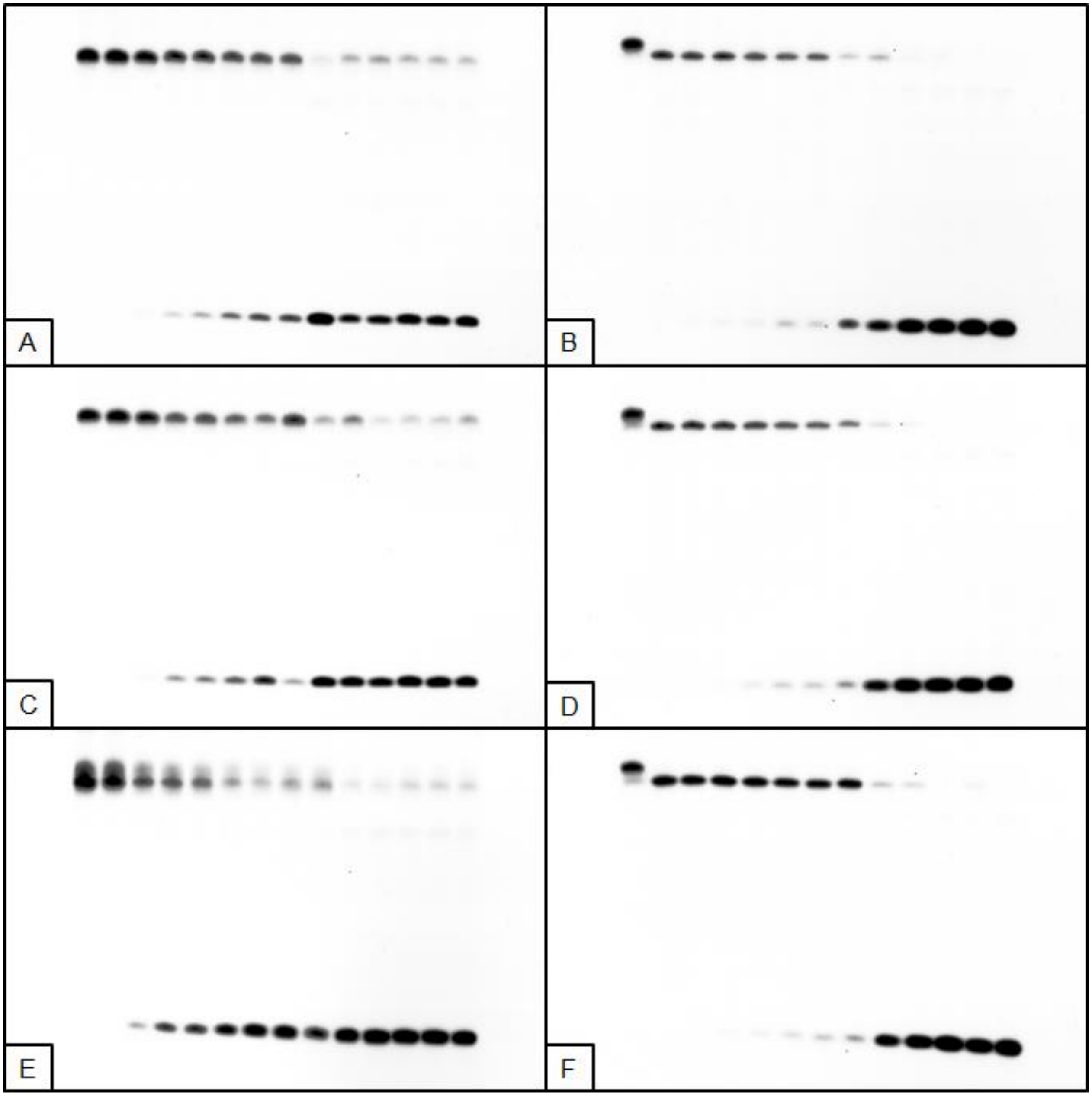

Figure S10. Cleavage assay of dU(-5)ScdA by 0.02U UDG and 0.5U hAPE1 (A,C,E) or 0.5U UDG and 0.02U hAPE1 (B,D,F). (A), (C) and (E) show 14 lanes each, which correspond to reaction times 0, 1, 5, 10, 15, 20, 25, 30, 35, 40, 45, 50, 55, and 60 min starting from the left. (B), (D) and (F) show 13 lanes each, which correspond to reaction times 0, 1, 5, 10, 15, 20, 25, 30, 60, 90, 120, 150 and 180 min starting from the left.

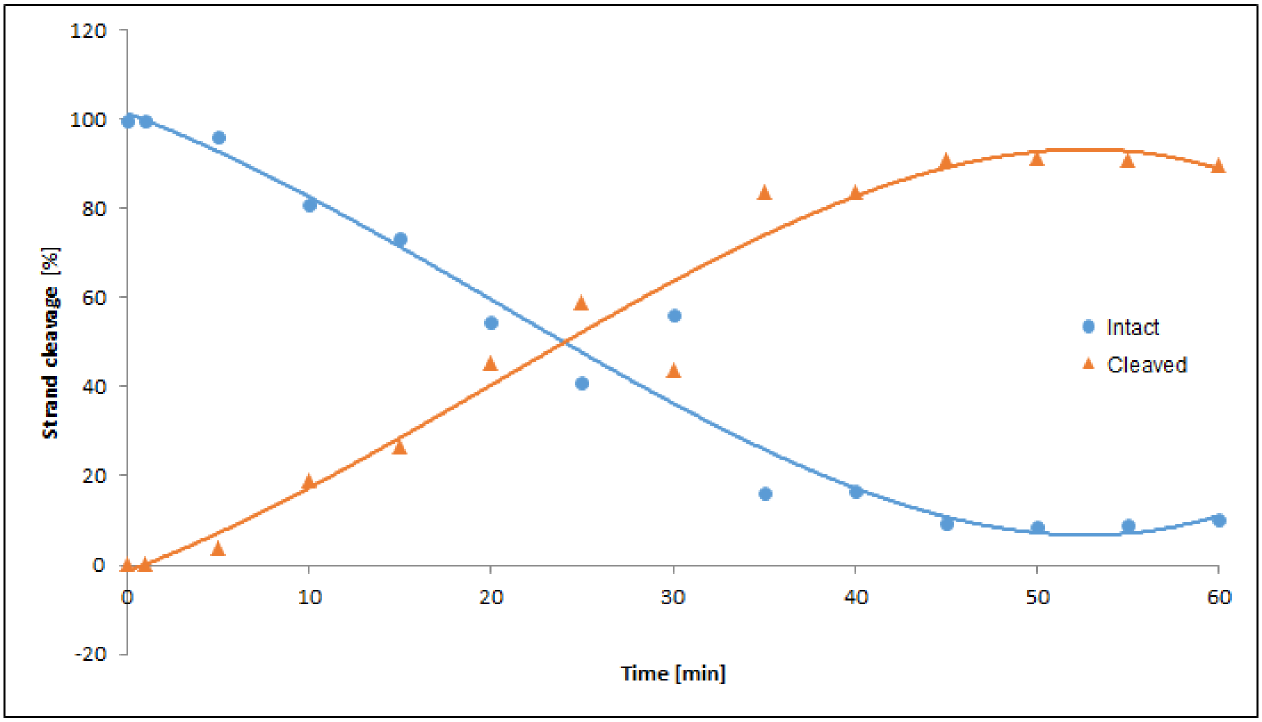

Figure S11. Cleavage assay of dU(-5)ScdA by 0.02U UDG and 0.5U hAPE1. The quantity loss of intact ssDNA (blue) and the quantity increase of SSB-DNA (orange).

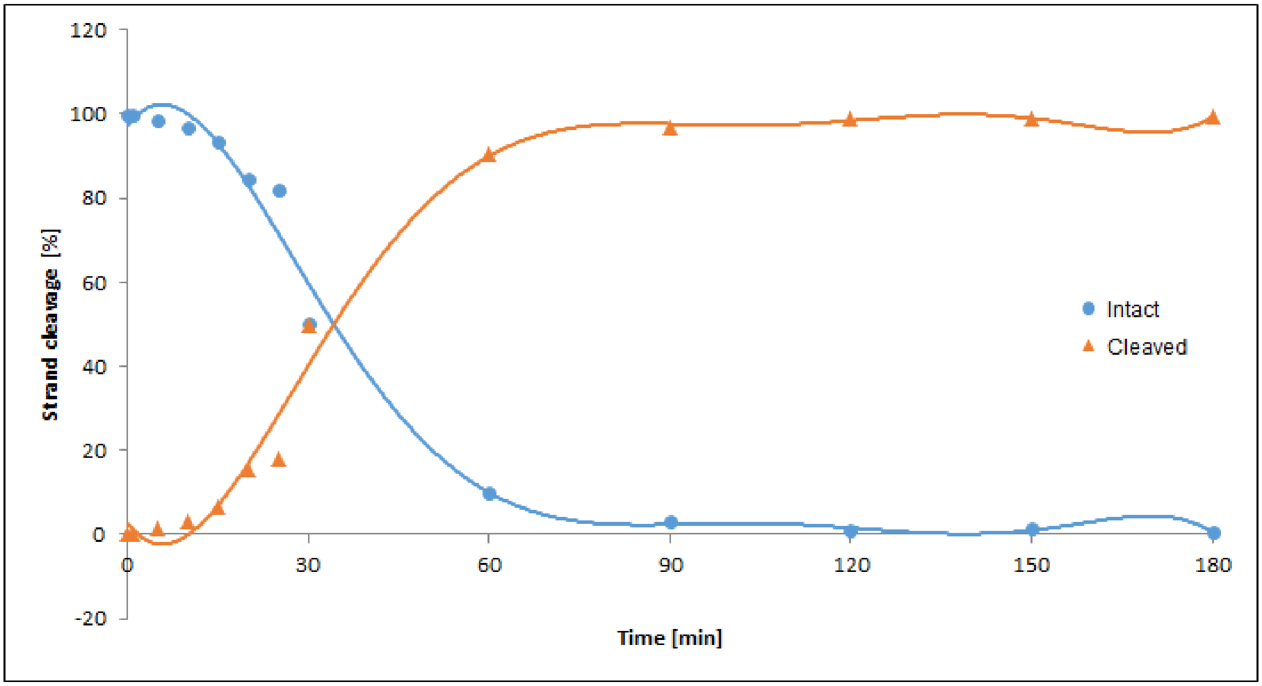

Figure S12. Cleavage assay of dU(-5)ScdA by 0.5U UDG and 0.02U hAPE1. The quantity loss of intact ssDNA (blue) and the quantity increase of SSB-DNA (orange).

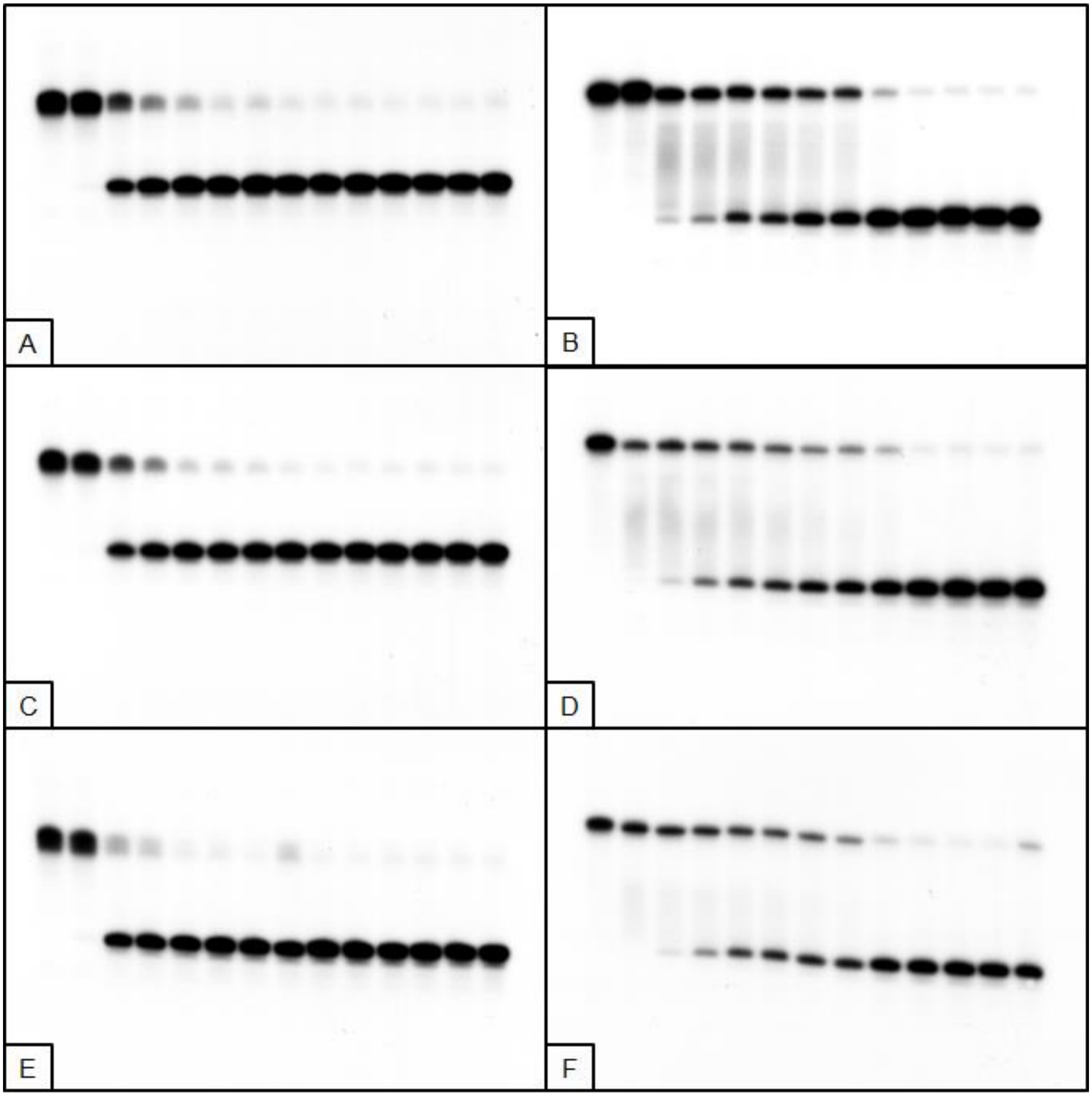

Figure S13. Cleavage assay of dU(+5)ScdA by 0.02U UDG and 0.5U hAPE1 (A,C,E) or 0.5U UDG and 0.02U hAPE1 (B,D,F). (A), (C) and (E) show 14 lanes each, which correspond to reaction times 0, 1, 5, 10, 15, 20, 25, 30, 35, 40, 45, 50, 55, and 60 min starting from the left. (B), (D) and (F) show 13 lanes each, which correspond to reaction times 0, 1, 5, 10, 15, 20, 25, 30, 60, 90, 120, 150 and 180 min starting from the left.

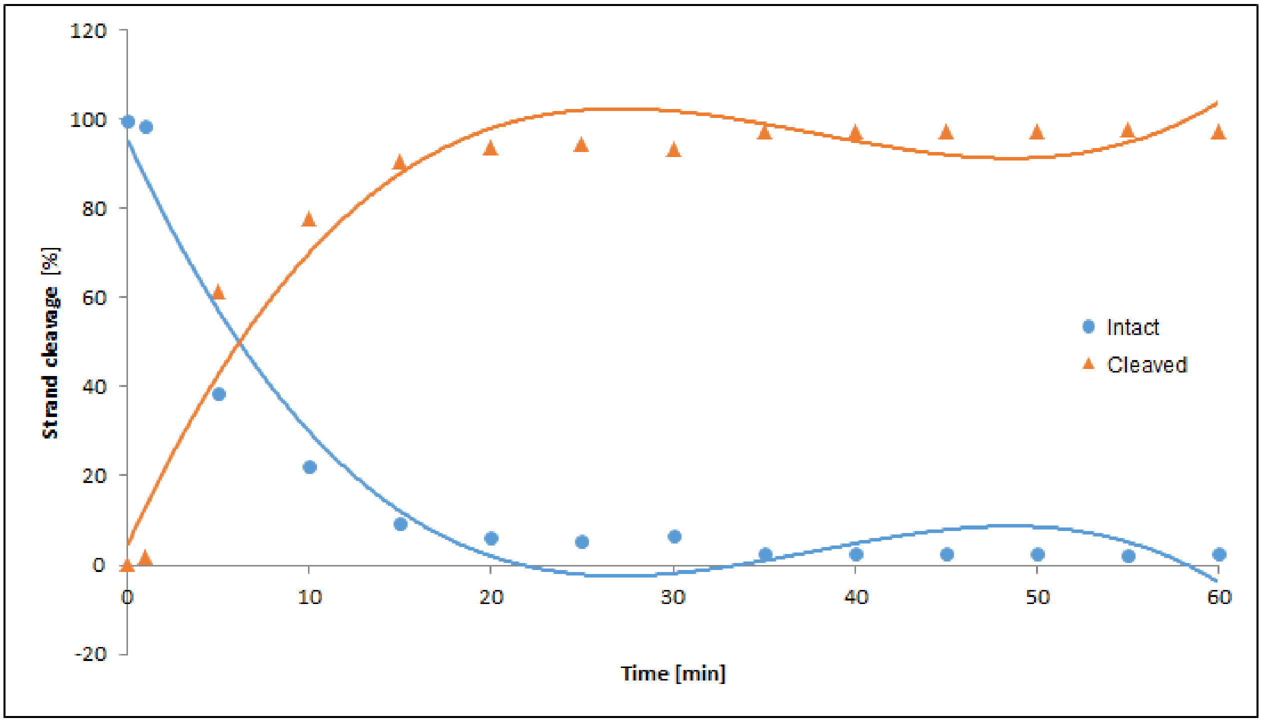

Figure S14. Cleavage assay of dU(+5)ScdA by 0.02U UDG and 0.5U hAPE1. The quantity loss of intact ssDNA (blue) and the quantity increase of SSB-DNA (orange).

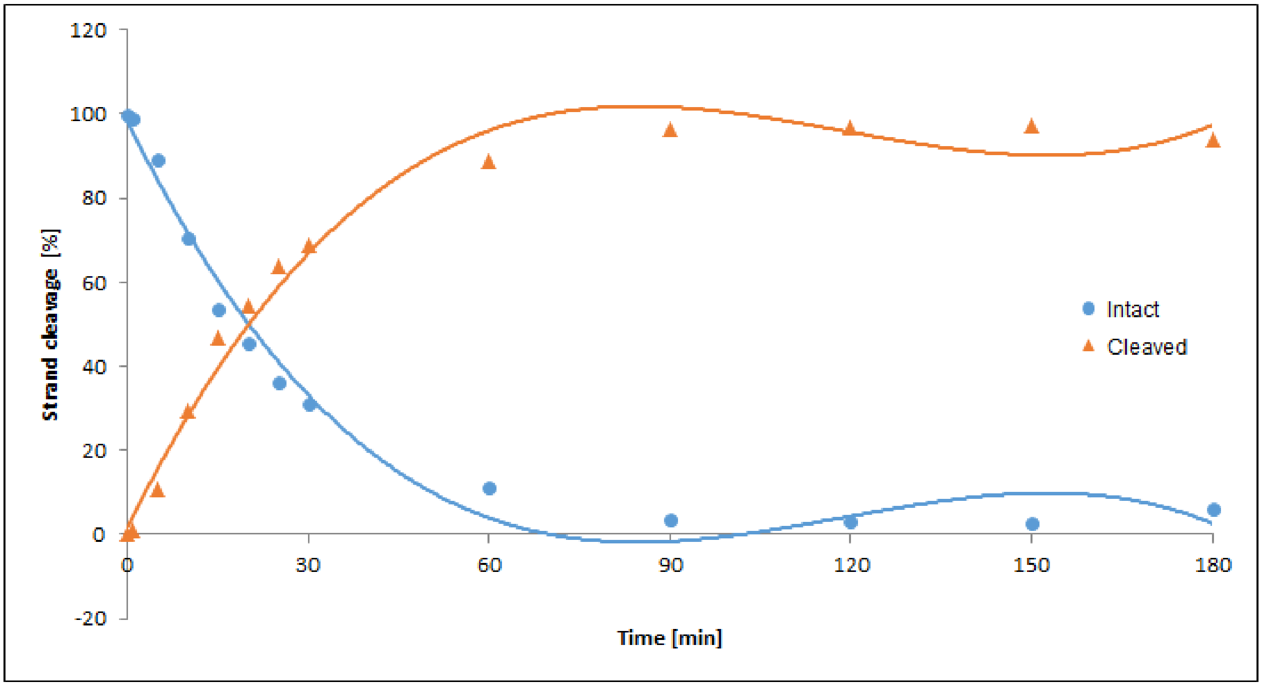

Figure S15. Cleavage assay of dU(+5)ScdA by 0.5U UDG and 0.02U hAPE1. The quantity loss of intact ssDNA (blue) and the quantity increase of SSB-DNA (orange).

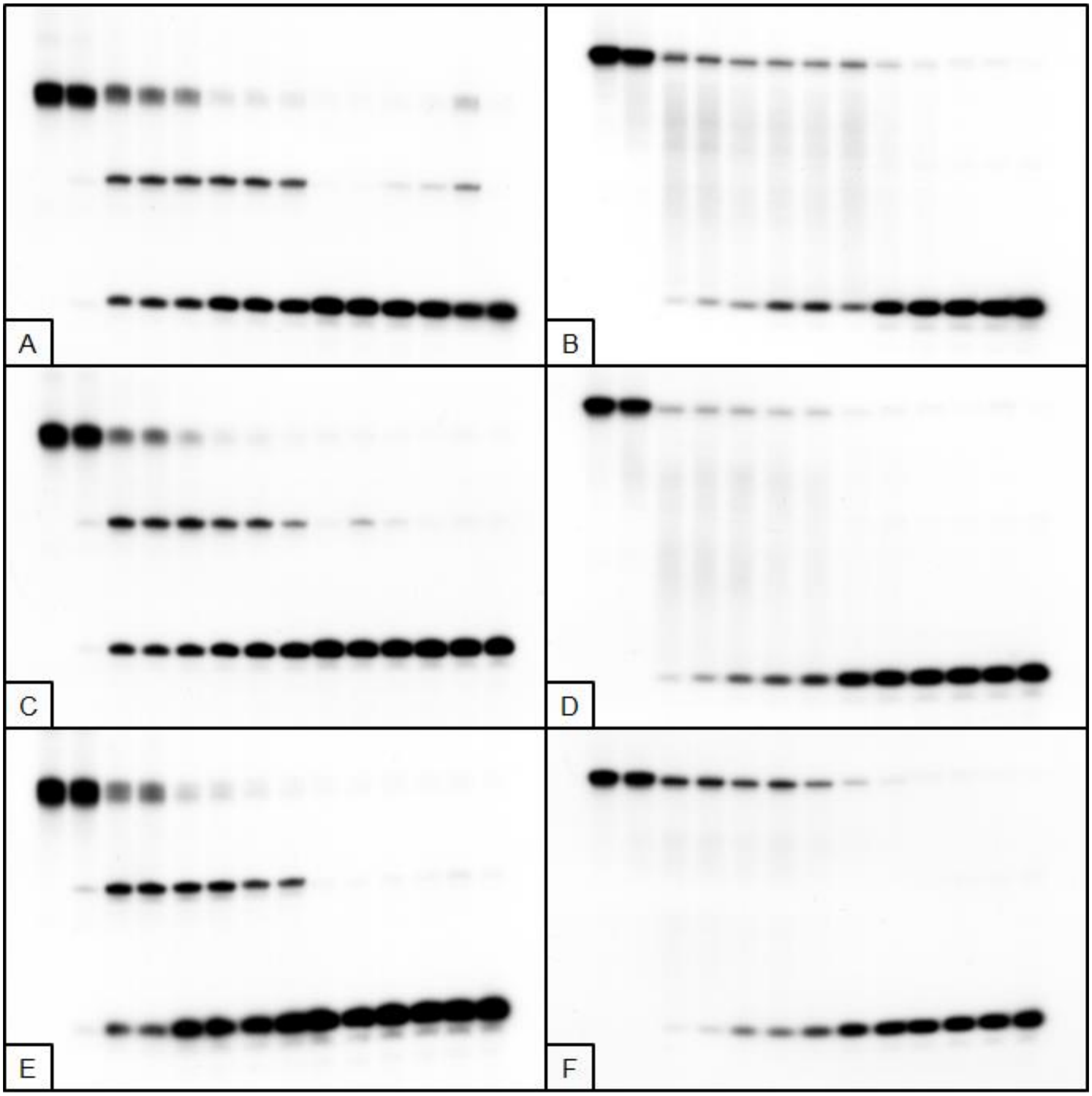

Figure S16. Cleavage assay of dU(-5)(+5)ScdA by 0.02U UDG and 0.5U hAPE1 (A,C,E) or 0.5U UDG and 0.02U hAPE1 (B,D,F). (A), (C) and (E) show 14 lanes each, which correspond to reaction times 0, 1, 5, 10, 15, 20, 25, 30, 35, 40, 45, 50, 55, and 60 min starting from the left. (B), (D) and (F) show 13 lanes each, which correspond to reaction times 0, 1, 5, 10, 15, 20, 25, 30, 60, 90, 120, 150 and 180 min starting from the left.

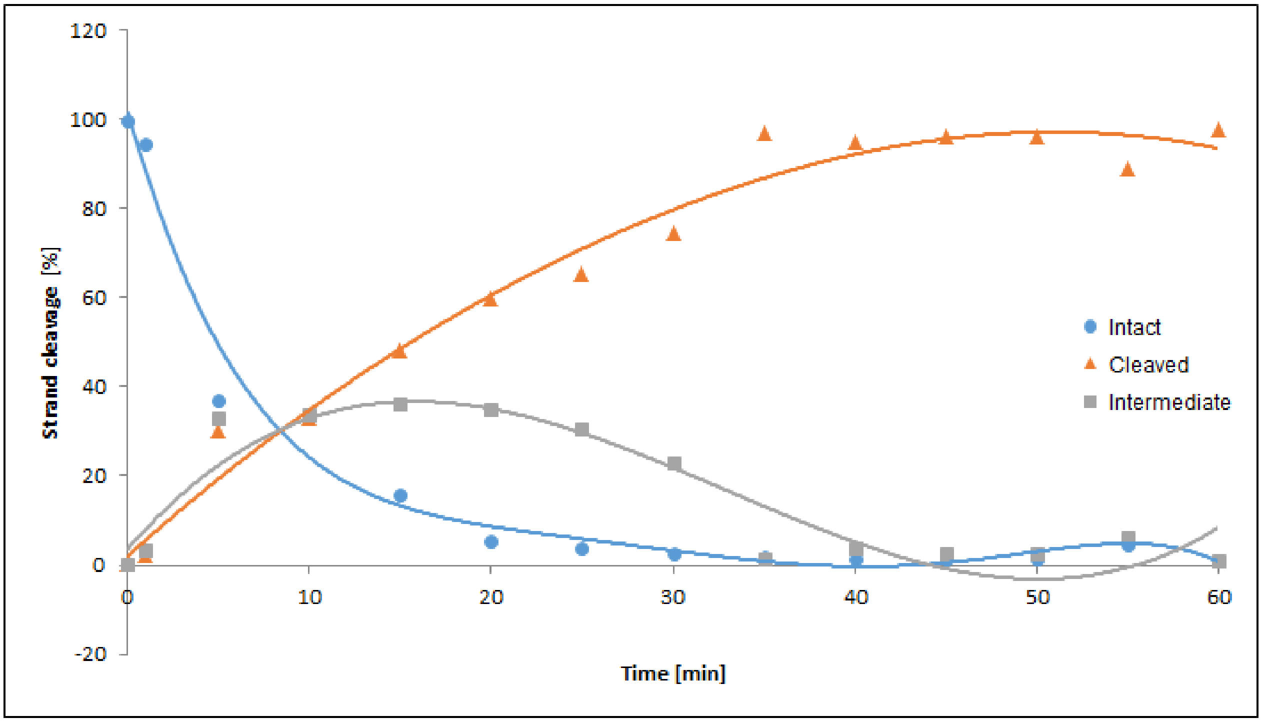

Figure S17. Cleavage assay of dU(-5)(+5)ScdA by 0.02U UDG and 0.5U hAPE1. The quantity loss of intact ssDNA (blue), the quantity increase of SSB-DNA (orange) and an intermediate oligo fragment (grey).

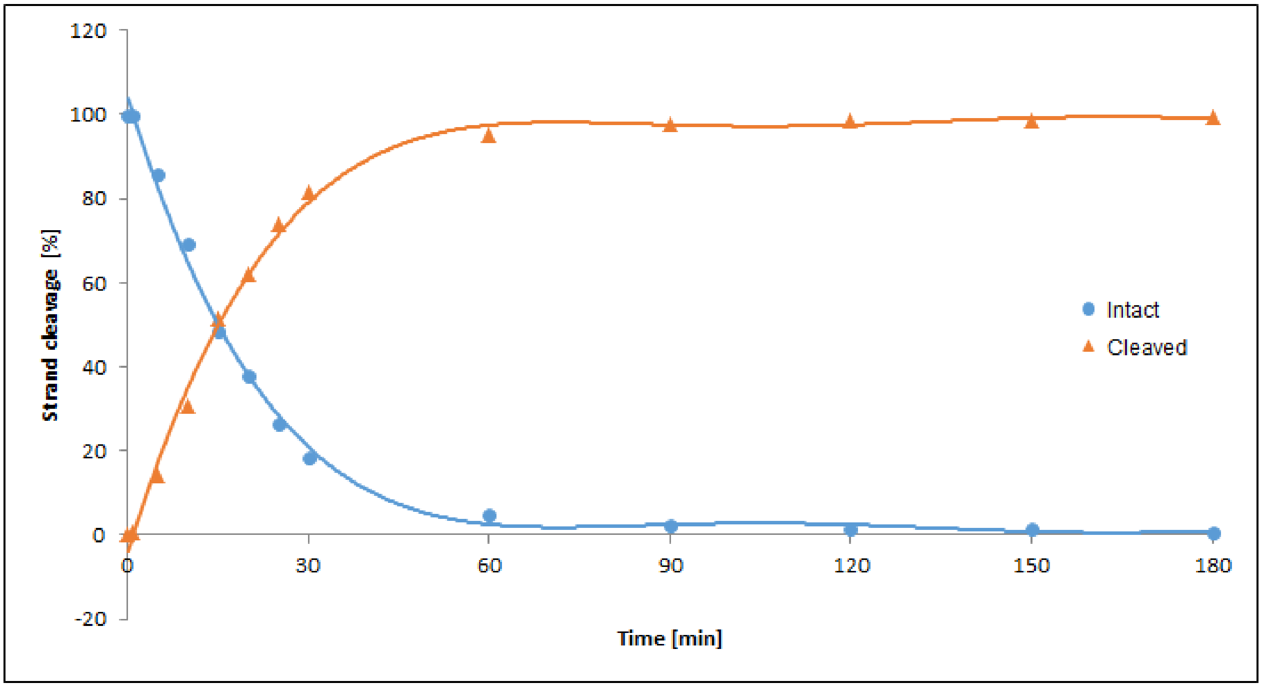

Figure S18. Cleavage assay of dU(-5)(+5)ScdA by 0.5U UDG and 0.02U hAPE1. The quantity loss of intact ssDNA (blue) and the quantity increase of SSB-DNA (orange).

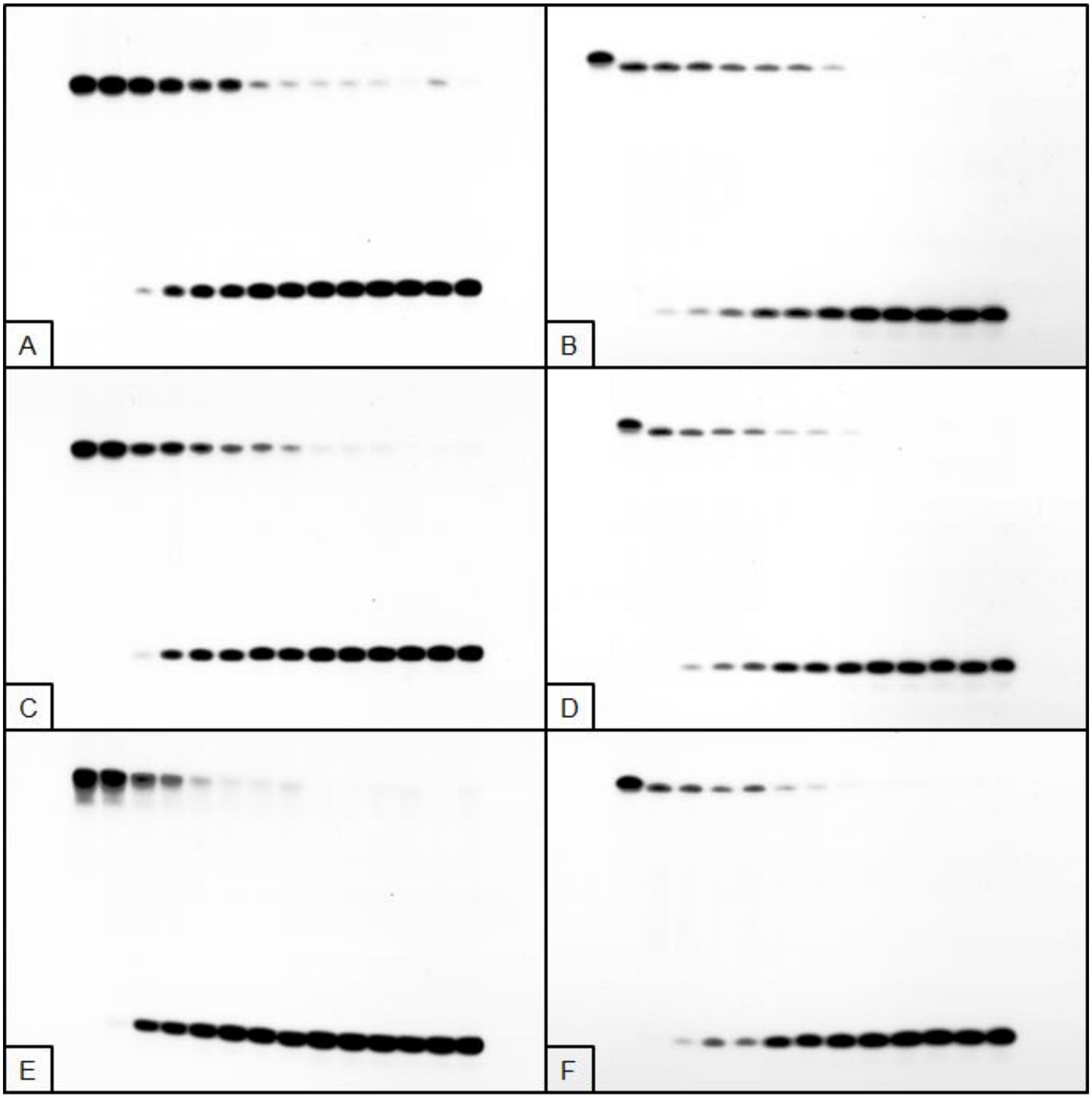

Figure S19. Cleavage assay of dU(-5)RcdA by 0.02U UDG and 0.5U hAPE1 (A,C,E) or 0.5U UDG and 0.02U hAPE1 (B,D,F). (A), (C) and (E) show 14 lanes each, which correspond to reaction times 0, 1, 5, 10, 15, 20, 25, 30, 35, 40, 45, 50, 55, and 60 min starting from the left. (B), (D) and (F) show 13 lanes each, which correspond to reaction times 0, 1, 5, 10, 15, 20, 25, 30, 60, 90, 120, 150 and 180 min starting from the left.

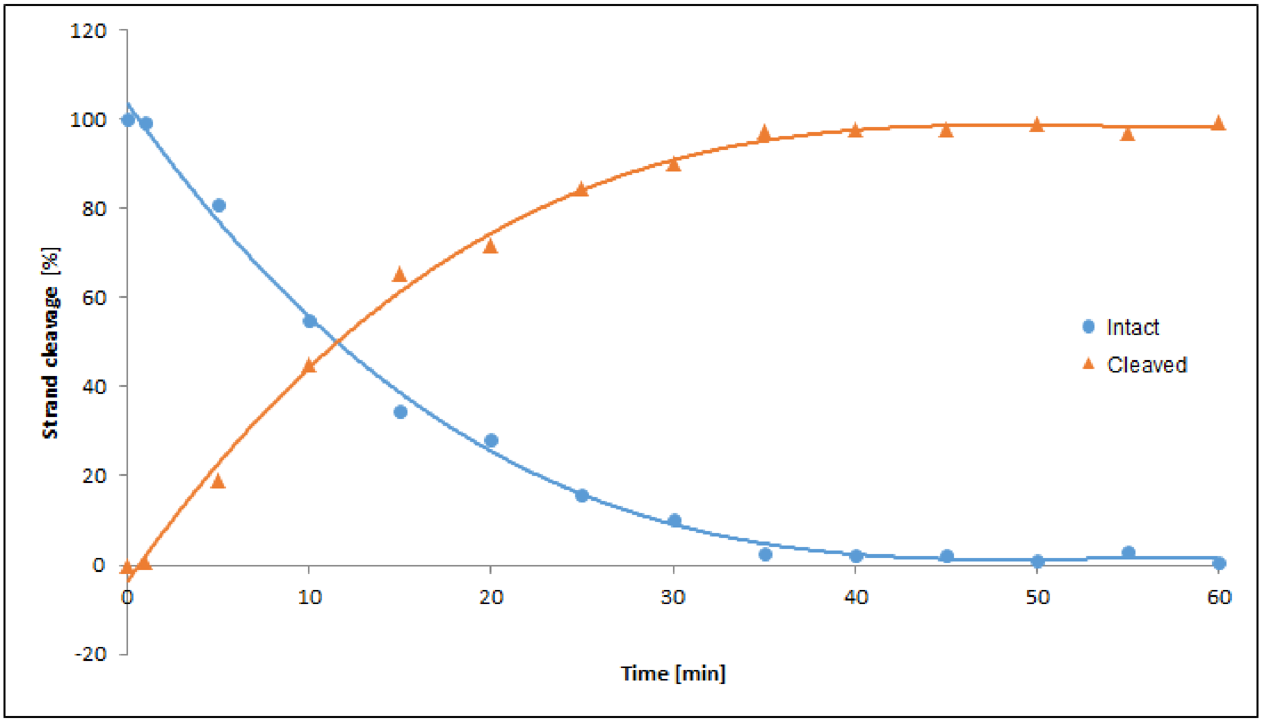

Figure S20. Cleavage assay of dU(-5)RcdA by 0.02U UDG and 0.5U hAPE1. The quantity loss of intact ssDNA (blue) and the quantity increase of SSB-DNA (orange).

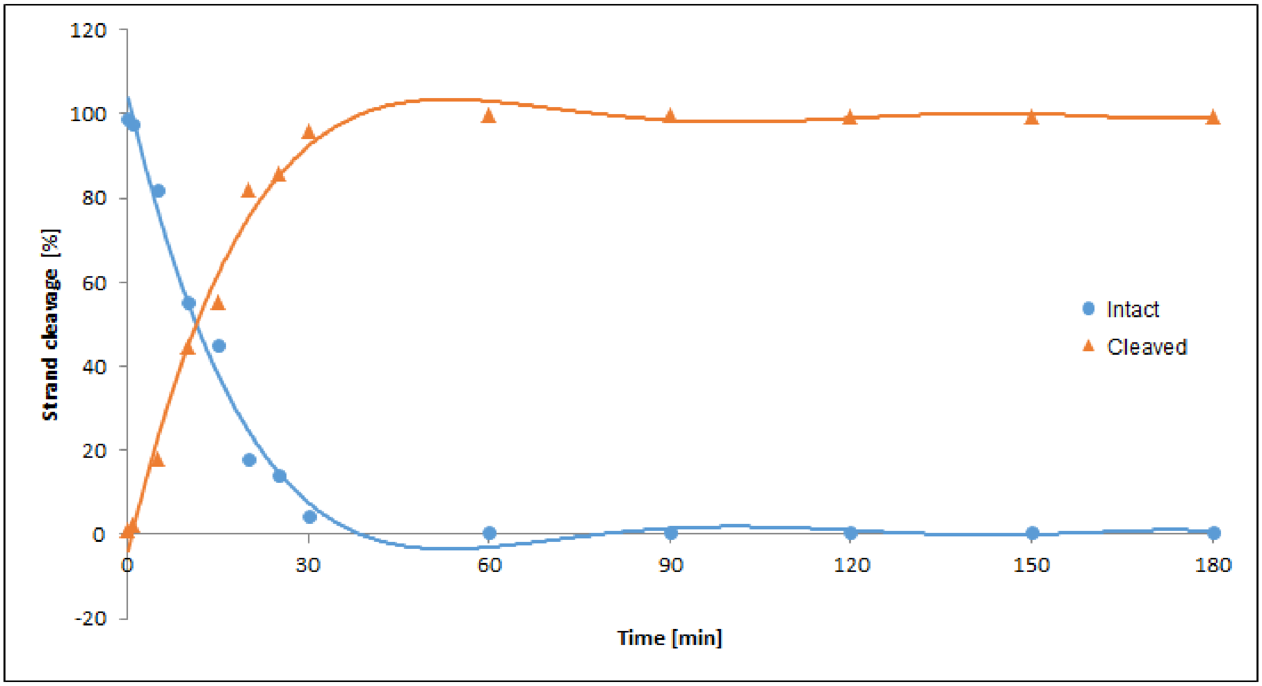

Figure S21. Cleavage assay of dU(-5)RcdA by 0.5U UDG and 0.02U hAPE1. The quantity loss of intact ssDNA (blue) and the quantity increase of SSB-DNA (orange).

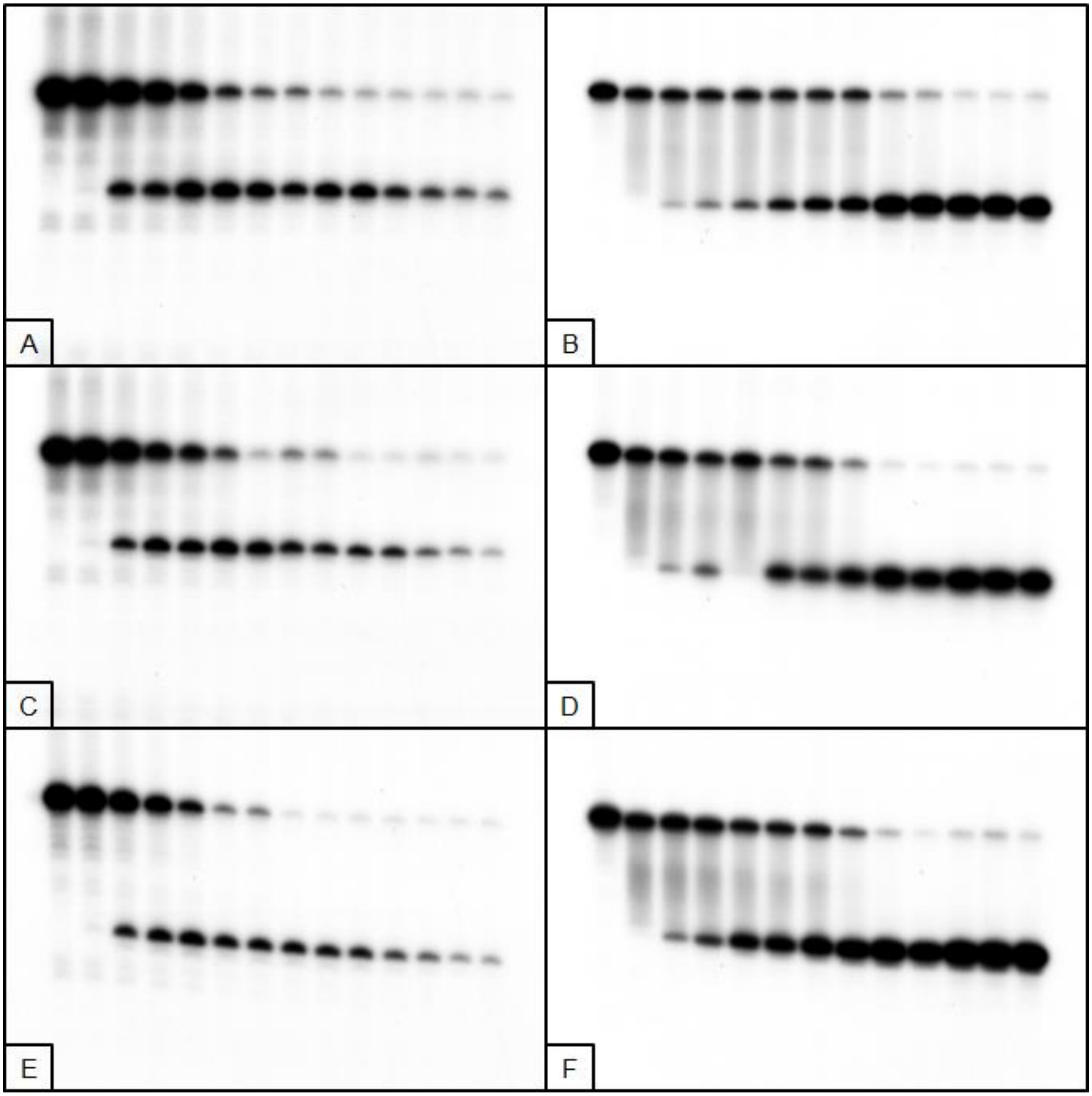

Figure S22. Cleavage assay of dU(+5)RcdA by 0.02U UDG and 0.5U hAPE1 (A,C,E) or 0.5U UDG and 0.02U hAPE1 (B,D,F). (A), (C) and (E) show 14 lanes each, which correspond to reaction times 0, 1, 5, 10, 15, 20, 25, 30, 35, 40, 45, 50, 55, and 60 min starting from the left. (B), (D) and (F) show 13 lanes each, which correspond to reaction times 0, 1, 5, 10, 15, 20, 25, 30, 60, 90, 120, 150 and 180 min starting from the left.

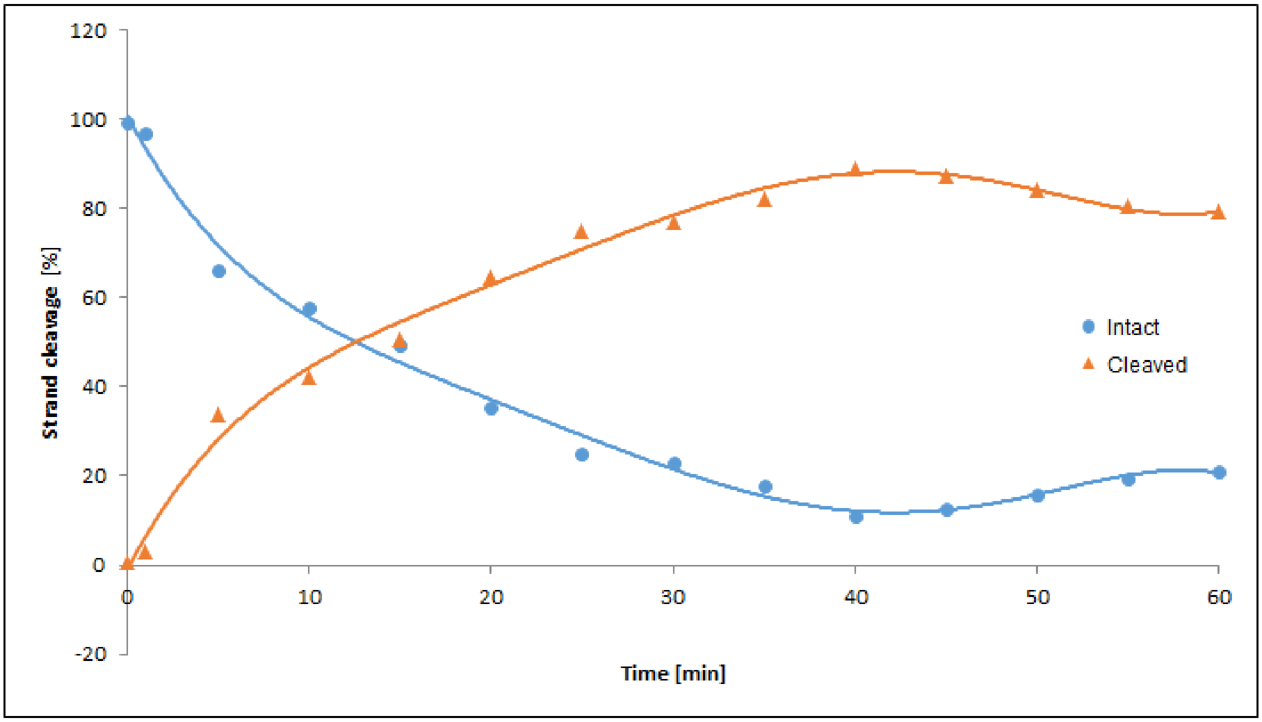

Figure S23. Cleavage assay of dU(+5)RcdA by 0.02U UDG and 0.5U hAPE1. The quantity loss of intact ssDNA (blue) and the quantity increase of SSB-DNA (orange).

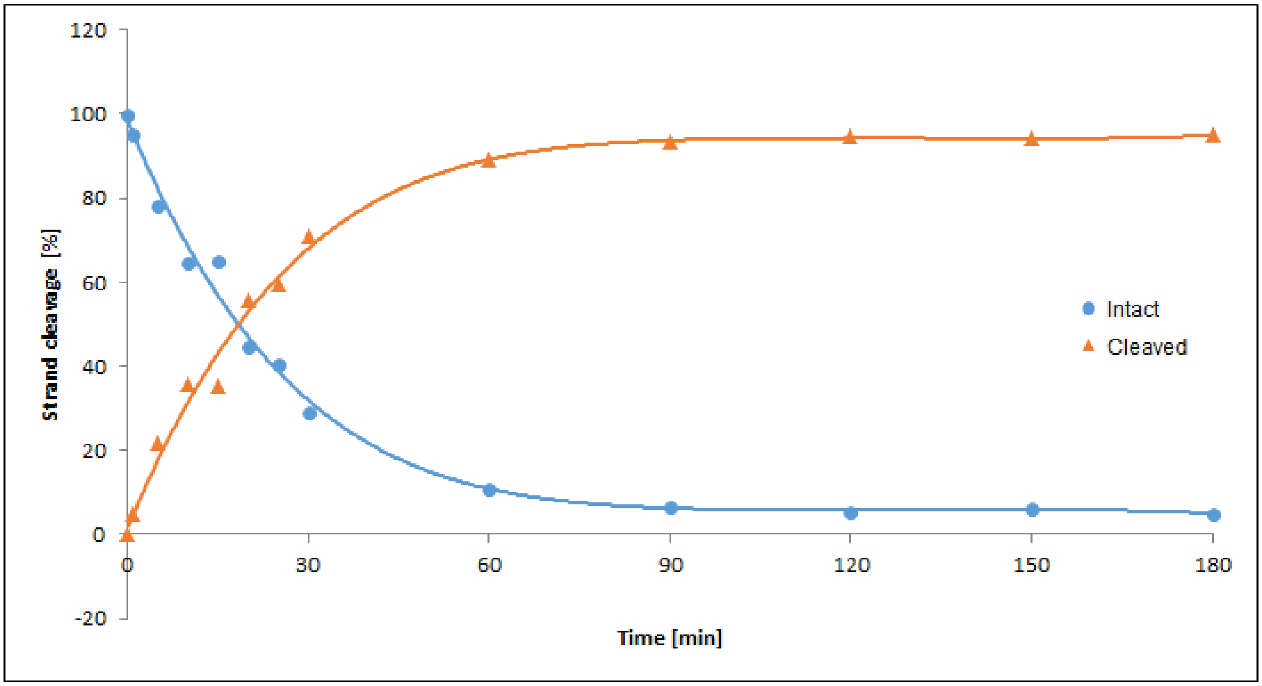

Figure S24. Cleavage assay of dU(+5)RcdA by 0.5U UDG and 0.02U hAPE1. The quantity loss of intact ssDNA (blue) and the quantity increase of SSB-DNA (orange).

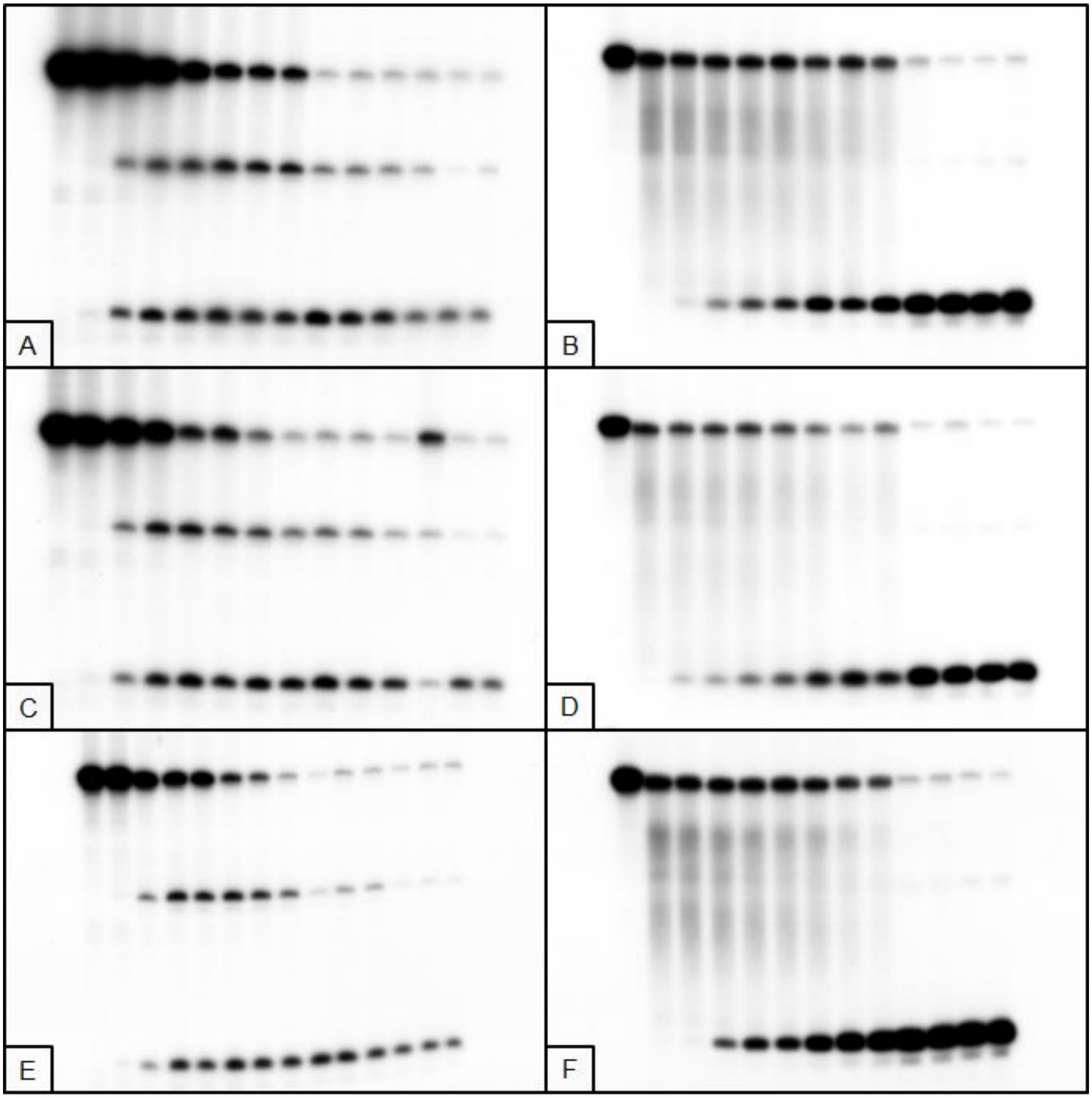

Figure S25. Cleavage assay of dU(-5)(+5)RcdA by 0.02U UDG and 0.5U hAPE1 (A,C,E) or 0.5U UDG and 0.02U hAPE1 (B,D,F). (A), (C) and (E) show 14 lanes each, which correspond to reaction times 0, 1, 5, 10, 15, 20, 25, 30, 35, 40, 45, 50, 55, and 60 min starting from the left. (B), (D) and (F) show 13 lanes each, which correspond to reaction times 0, 1, 5, 10, 15, 20, 25, 30, 60, 90, 120, 150 and 180 min starting from the left.

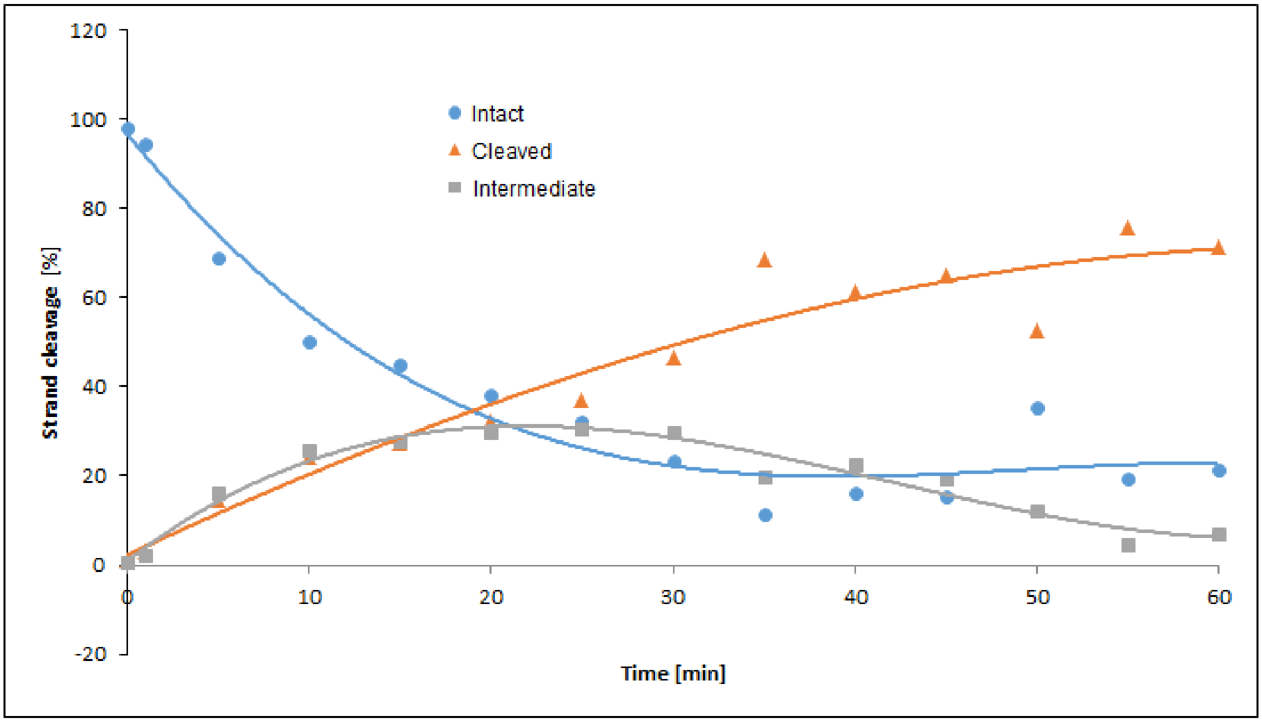

Figure S26. Cleavage assay of dU(-5)(+5)RcdA by 0.02U UDG and 0.5U hAPE1. The quantity loss of intact ssDNA (blue), the quantity increase of SSB-DNA (orange) and an intermediate oligo fragment (grey).

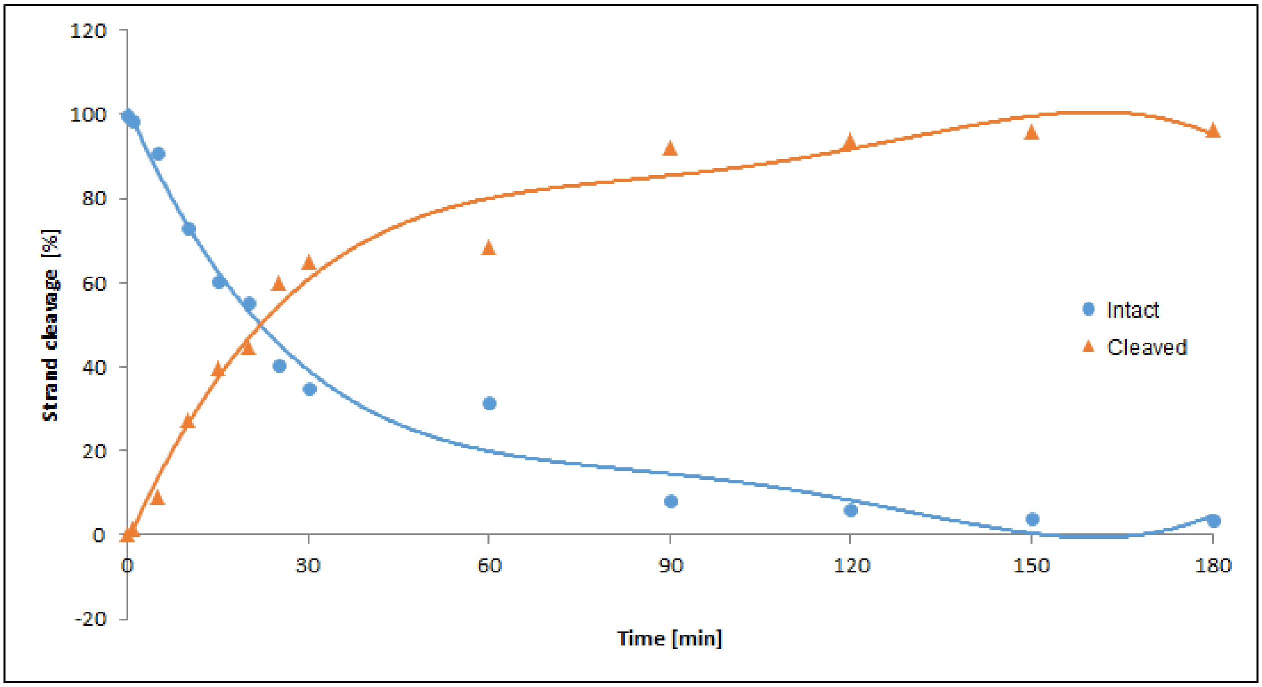

Figure S27. Cleavage assay of dU(-5)(+5)RcdA by 0.5U UDG and 0.02U hAPE1. The quantity loss of intact ssDNA (blue) and the quantity increase of SSB-DNA (orange).

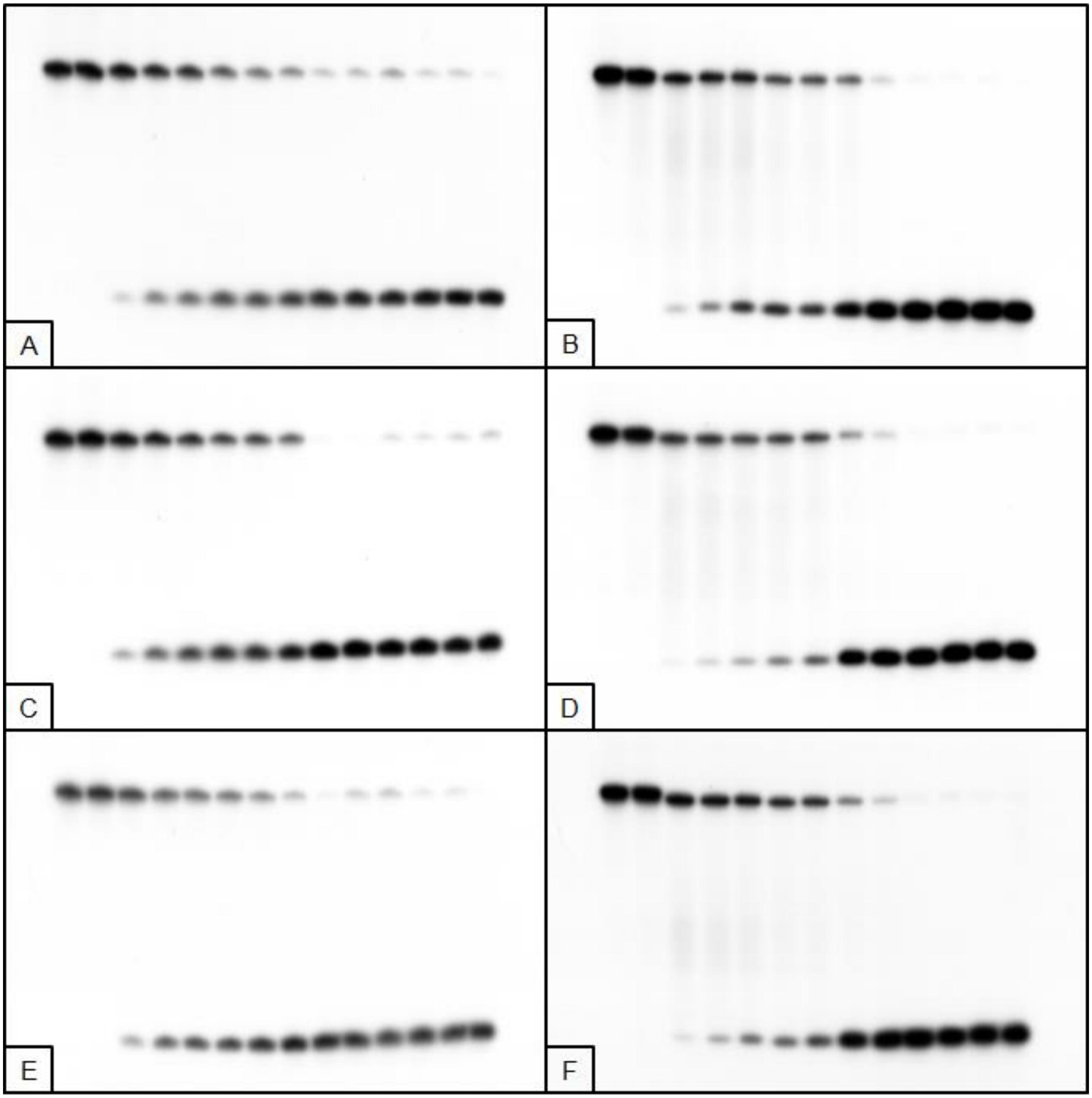

Figure S28. Cleavage assay of dU(-5)ScdG by 0.02U UDG and 0.5U hAPE1 (A,C,E) or 0.5U UDG and 0.02U hAPE1 (B,D,F). (A), (C) and (E) show 14 lanes each, which correspond to reaction times 0, 1, 5, 10, 15, 20, 25, 30, 35, 40, 45, 50, 55, and 60 min starting from the left. (B), (D) and (F) show 13 lanes each, which correspond to reaction times 0, 1, 5, 10, 15, 20, 25, 30, 60, 90, 120, 150 and 180 min starting from the left.

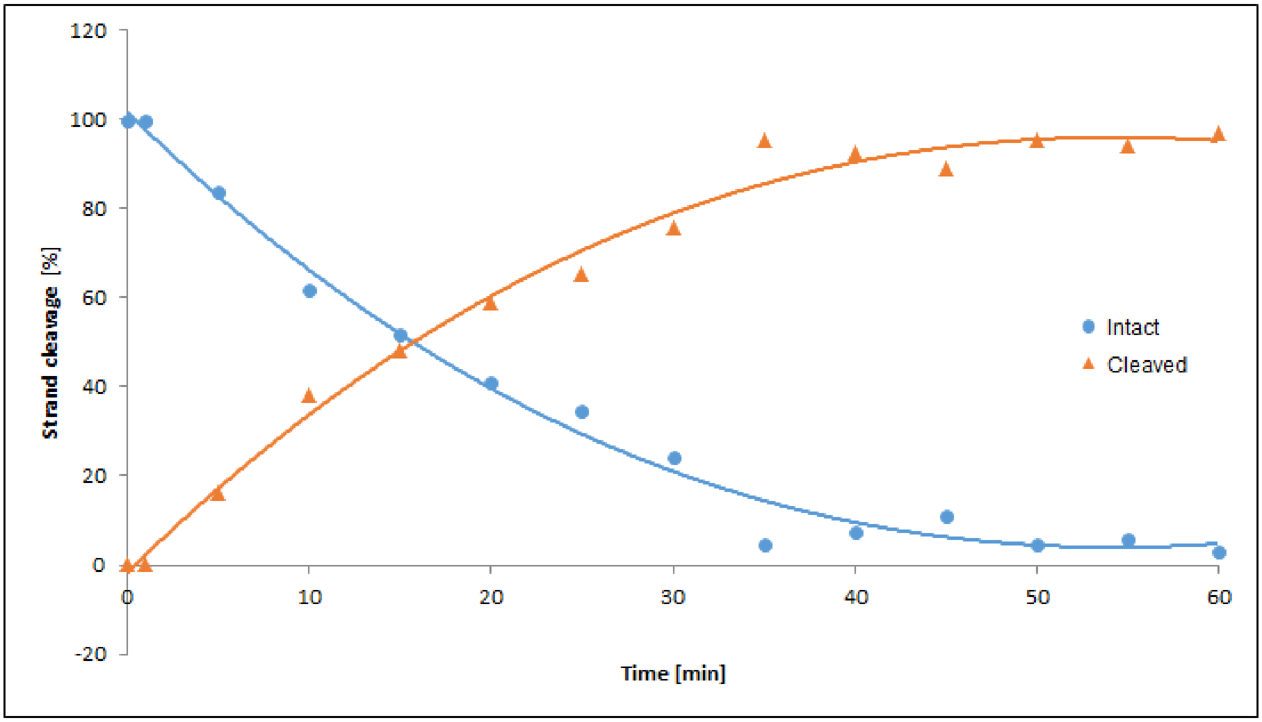

Figure S29. Cleavage assay of dU(-5)ScdG by 0.02U UDG and 0.5U hAPE1. The quantity loss of intact ssDNA (blue) and the quantity increase of SSB-DNA (orange).

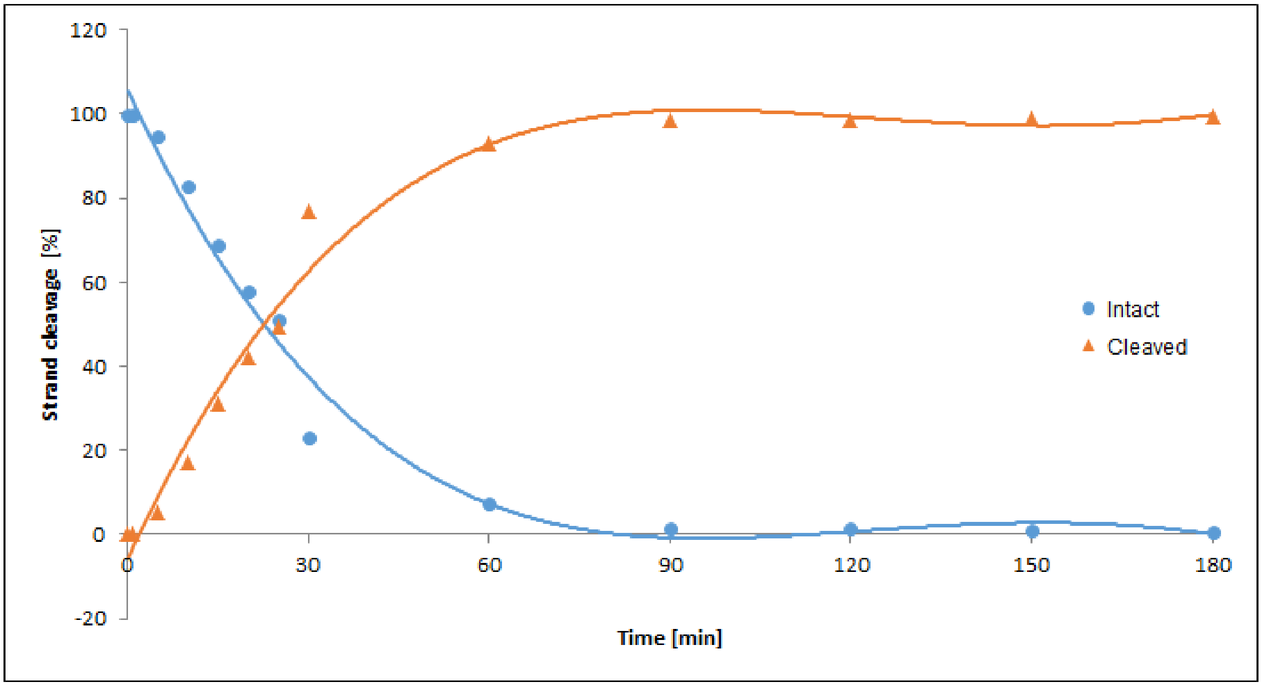

Figure S30. Cleavage assay of dU(-5)ScdG by 0.5U UDG and 0.02U hAPE1. The quantity loss of intact ssDNA (blue) and the quantity increase of SSB-DNA (orange).

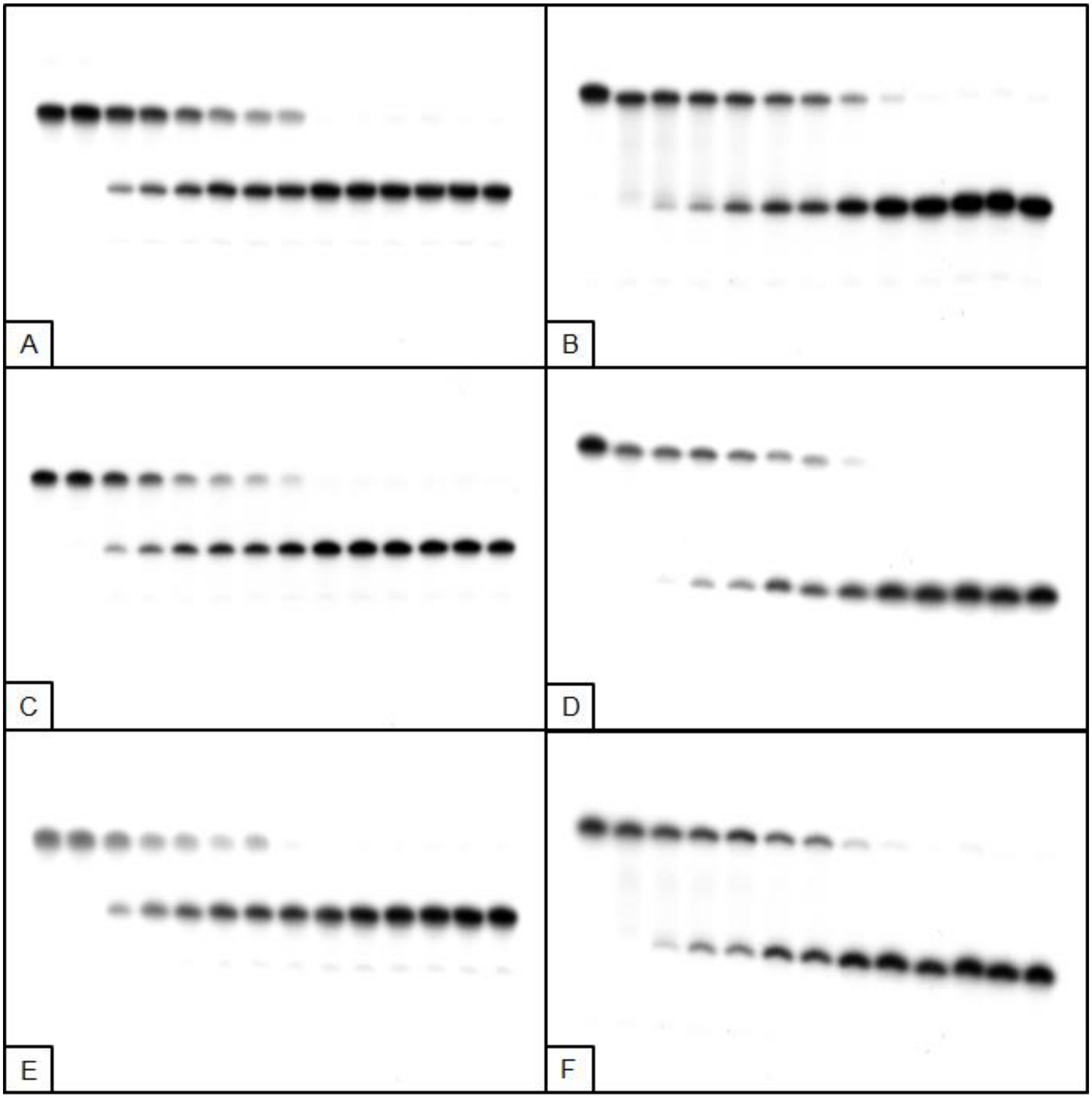

Figure S31. Cleavage assay of dU(+5)ScdG by 0.02U UDG and 0.5U hAPE1 (A,C,E) or 0.5U UDG and 0.02U hAPE1 (B,D,F). (A), (C) and (E) show 14 lanes each, which correspond to reaction times 0, 1, 5, 10, 15, 20, 25, 30, 35, 40, 45, 50, 55, and 60 min starting from the left. (B), (D) and (F) show 13 lanes each, which correspond to reaction times 0, 1, 5, 10, 15, 20, 25, 30, 60, 90, 120, 150 and 180 min starting from the left.

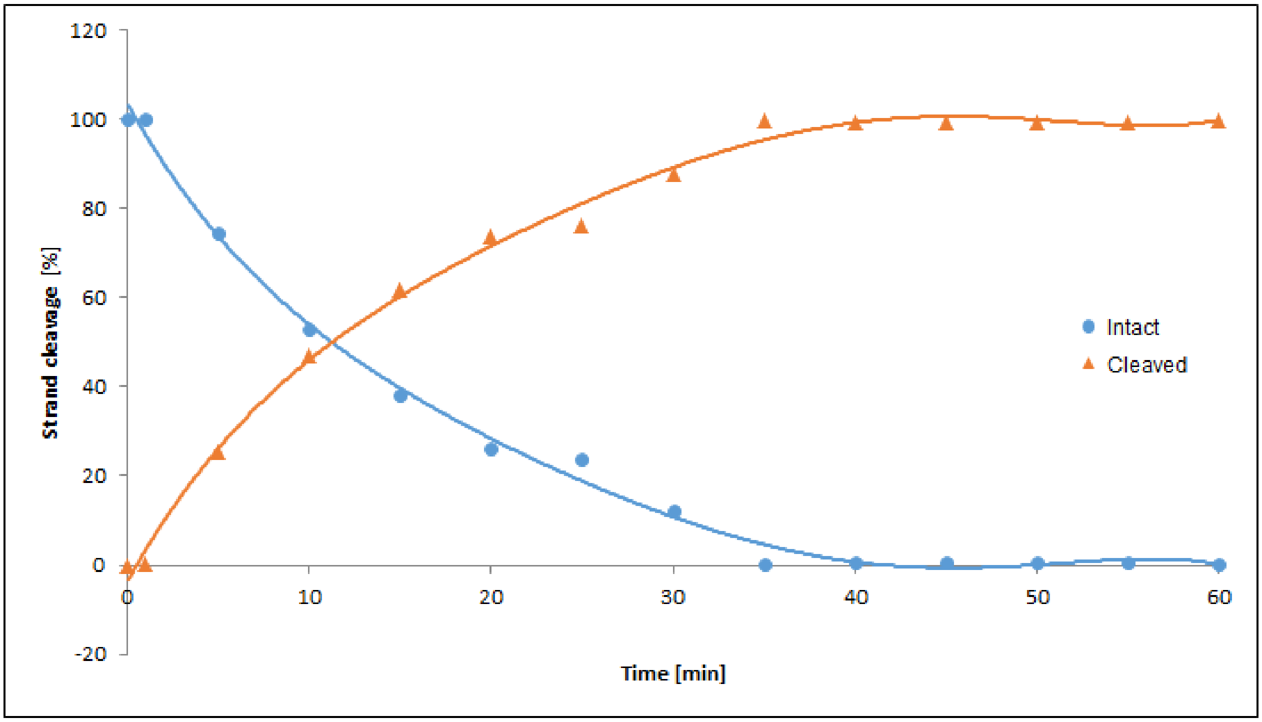

Figure S32. Cleavage assay of dU(+5)ScdG by 0.02U UDG and 0.5U hAPE1. The quantity loss of intact ssDNA (blue) and the quantity increase of SSB-DNA (orange).

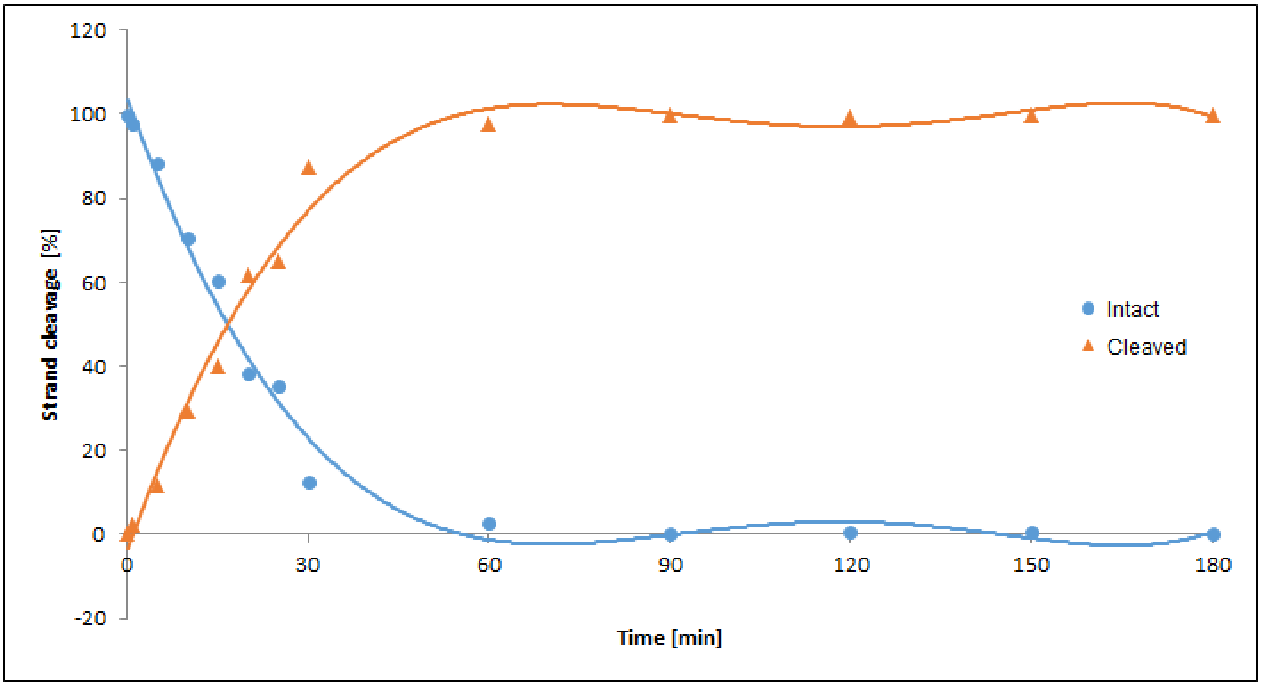

Figure S33. Cleavage assay of dU(+5)ScdG by 0.5U UDG and 0.02U hAPE1. The quantity loss of intact ssDNA (blue) and the quantity increase of SSB-DNA (orange).

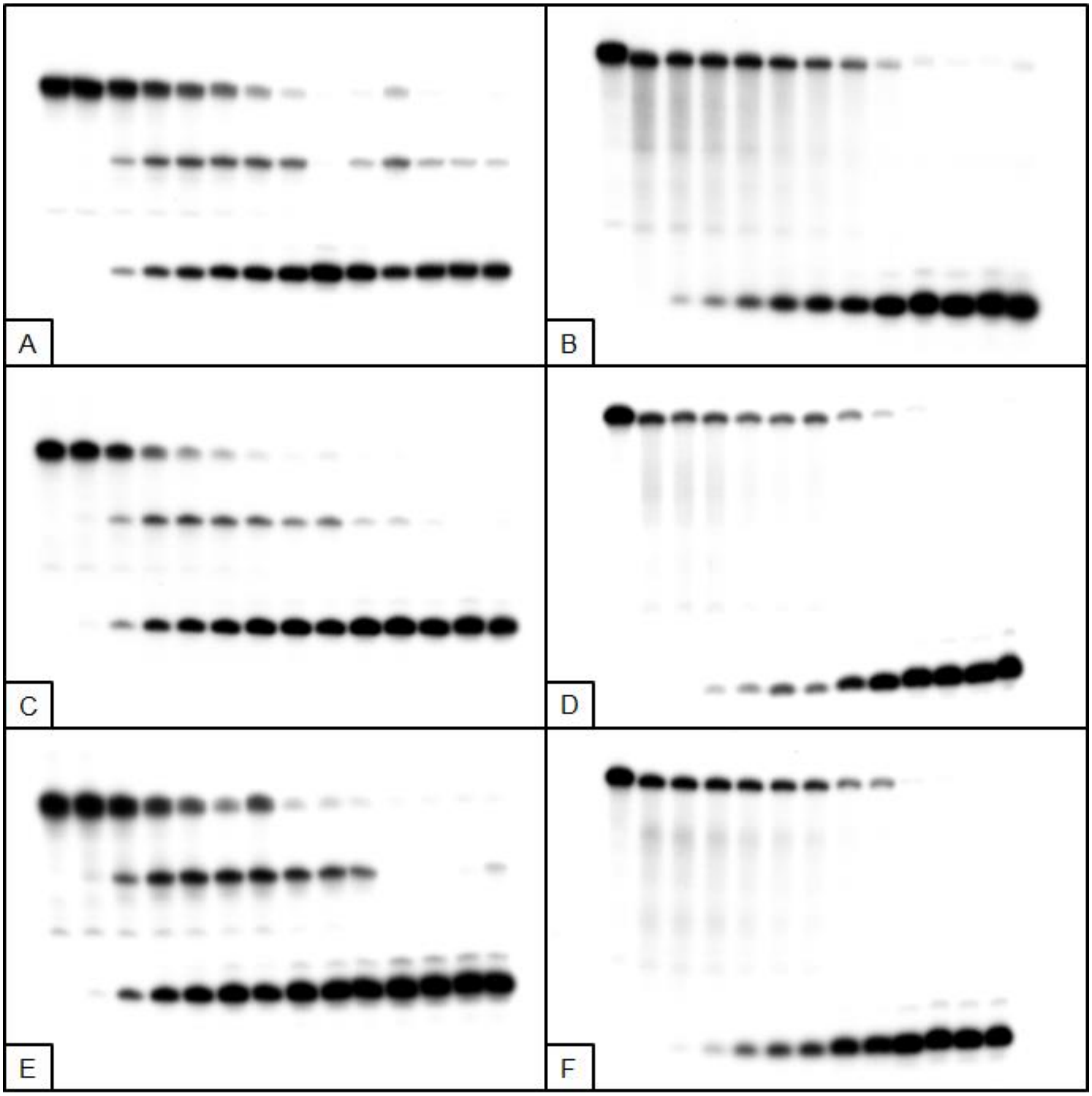

Figure S34. Cleavage assay of dU(-5)(+5)ScdG by 0.02U UDG and 0.5U hAPE1 (A,C,E) or 0.5U UDG and 0.02U hAPE1 (B,D,F). (A), (C) and (E) show 14 lanes each, which correspond to reaction times 0, 1, 5, 10, 15, 20, 25, 30, 35, 40, 45, 50, 55, and 60 min starting from the left. (B), (D) and (F) show 13 lanes each, which correspond to reaction times 0, 1, 5, 10, 15, 20, 25, 30, 60, 90, 120, 150 and 180 min starting from the left.

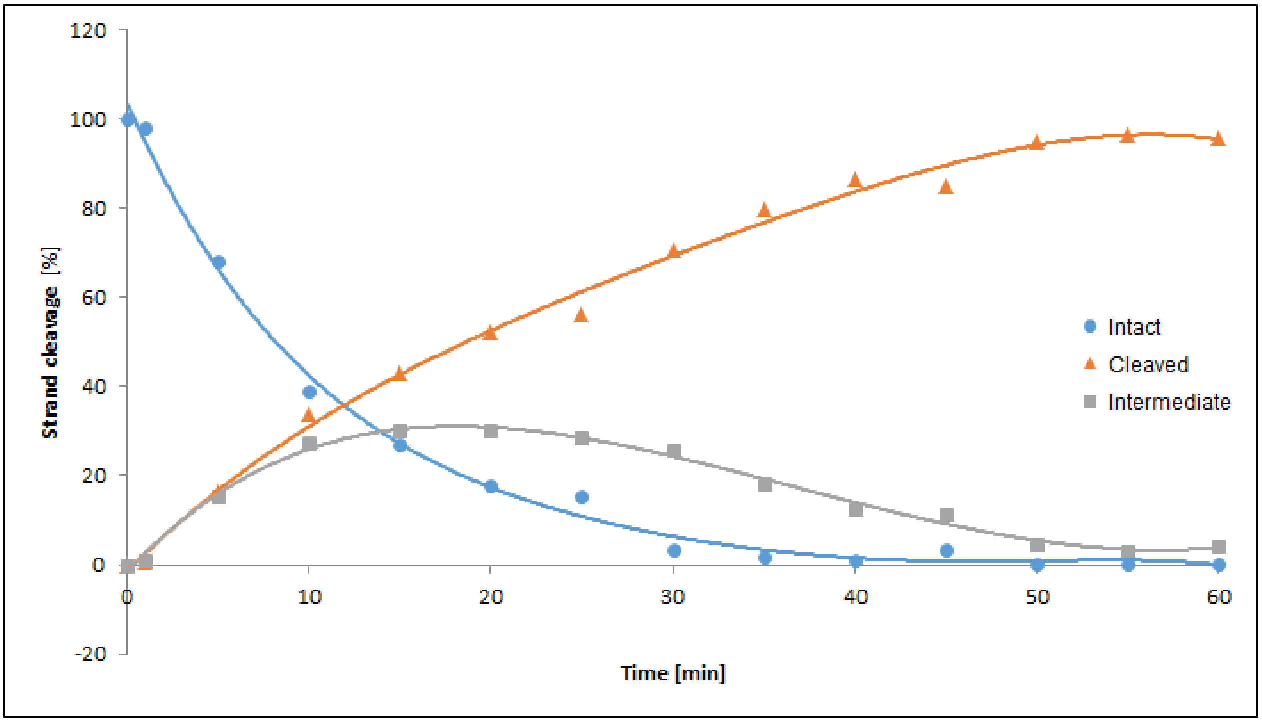

Figure S35. Cleavage assay of dU(-5)(+5)ScdG by 0.02U UDG and 0.5U hAPE1. The quantity loss of intact ssDNA (blue), the quantity increase of SSB-DNA (orange) and an intermediate oligo fragment (grey).

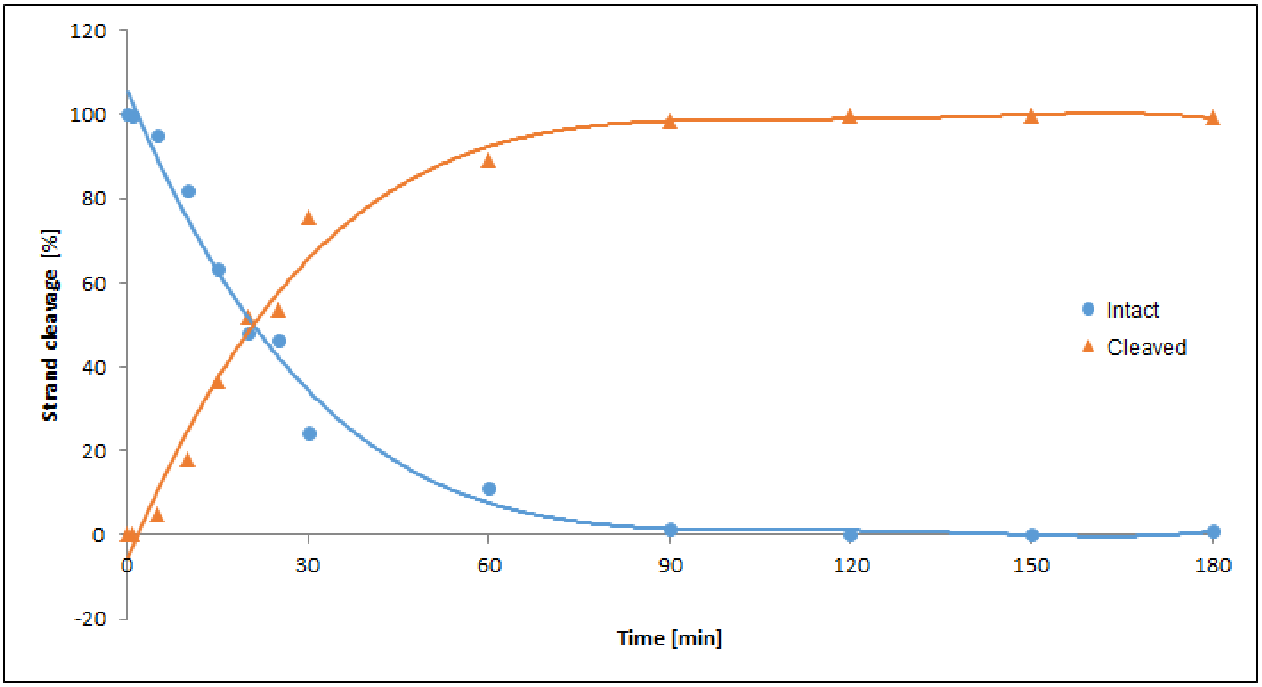

Figure S36. Cleavage assay of dU(-5)(+5)ScdG by 0.5U UDG and 0.02U hAPE1. The quantity loss of intact ssDNA (blue) and the quantity increase of SSB-DNA (orange).

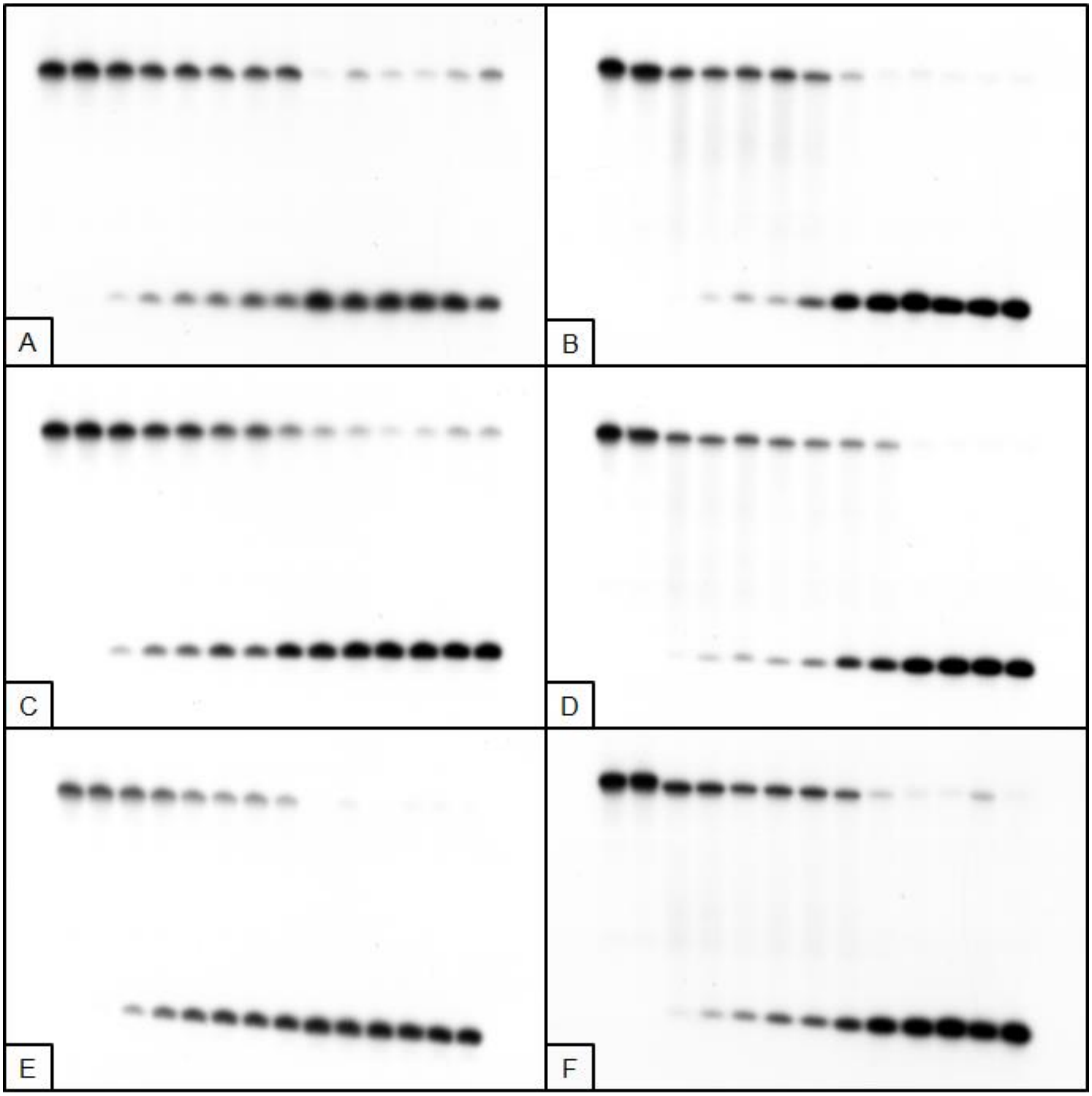

Figure S37. Cleavage assay of dU(-5)RcdG by 0.02U UDG and 0.5U hAPE1 (A,C,E) or 0.5U UDG and 0.02U hAPE1 (B,D,F). (A), (C) and (E) show 14 lanes each, which correspond to reaction times 0, 1, 5, 10, 15, 20, 25, 30, 35, 40, 45, 50, 55, and 60 min starting from the left. (B), (D) and (F) show 13 lanes each, which correspond to reaction times 0, 1, 5, 10, 15, 20, 25, 30, 60, 90, 120, 150 and 180 min starting from the left.

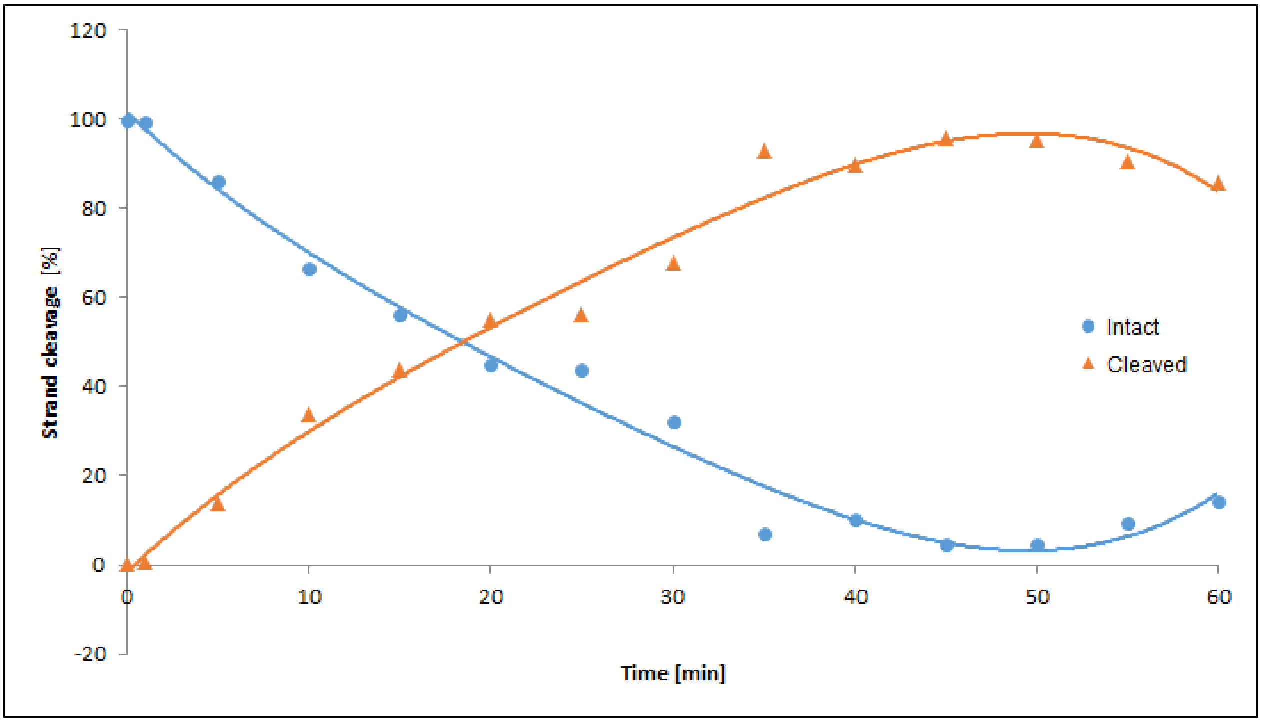

Figure S38. Cleavage assay of dU(-5)RcdG by 0.02U UDG and 0.5U hAPE1. The quantity loss of intact ssDNA (blue) and the quantity increase of SSB-DNA (orange).

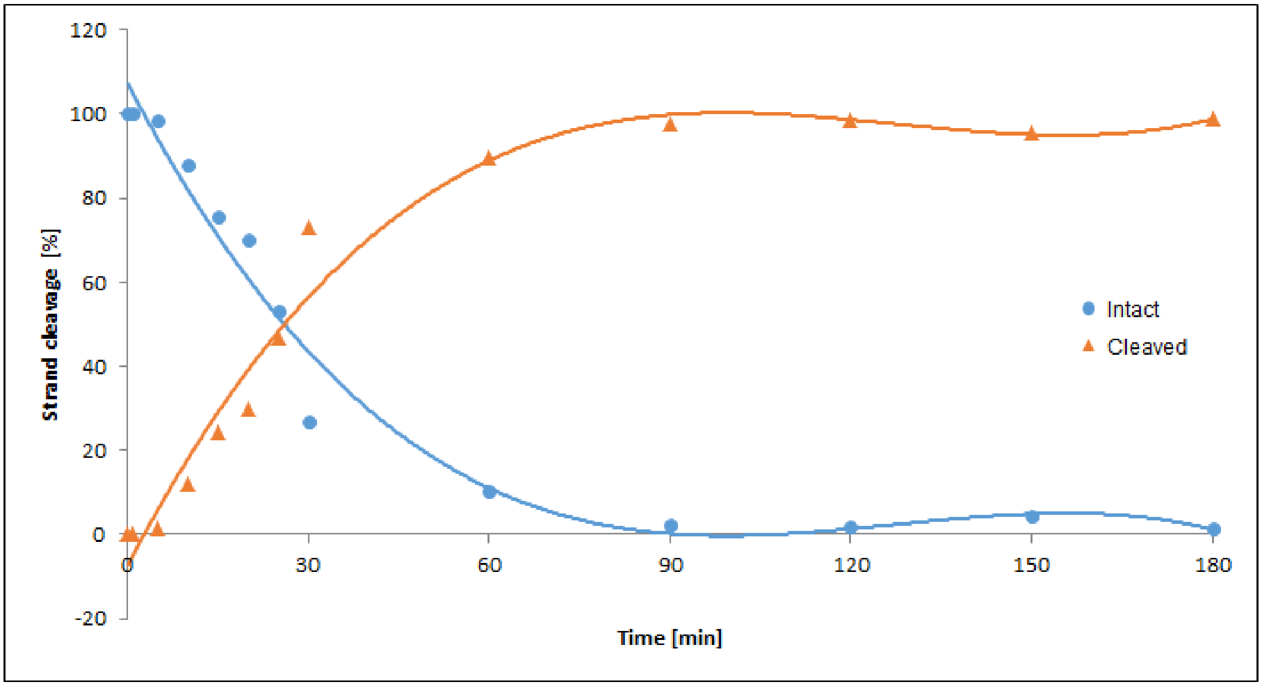

Figure S39. Cleavage assay of dU(-5)RcdG by 0.5U UDG and 0.02U hAPE1. The quantity loss of intact ssDNA (blue) and the quantity increase of SSB-DNA (orange).

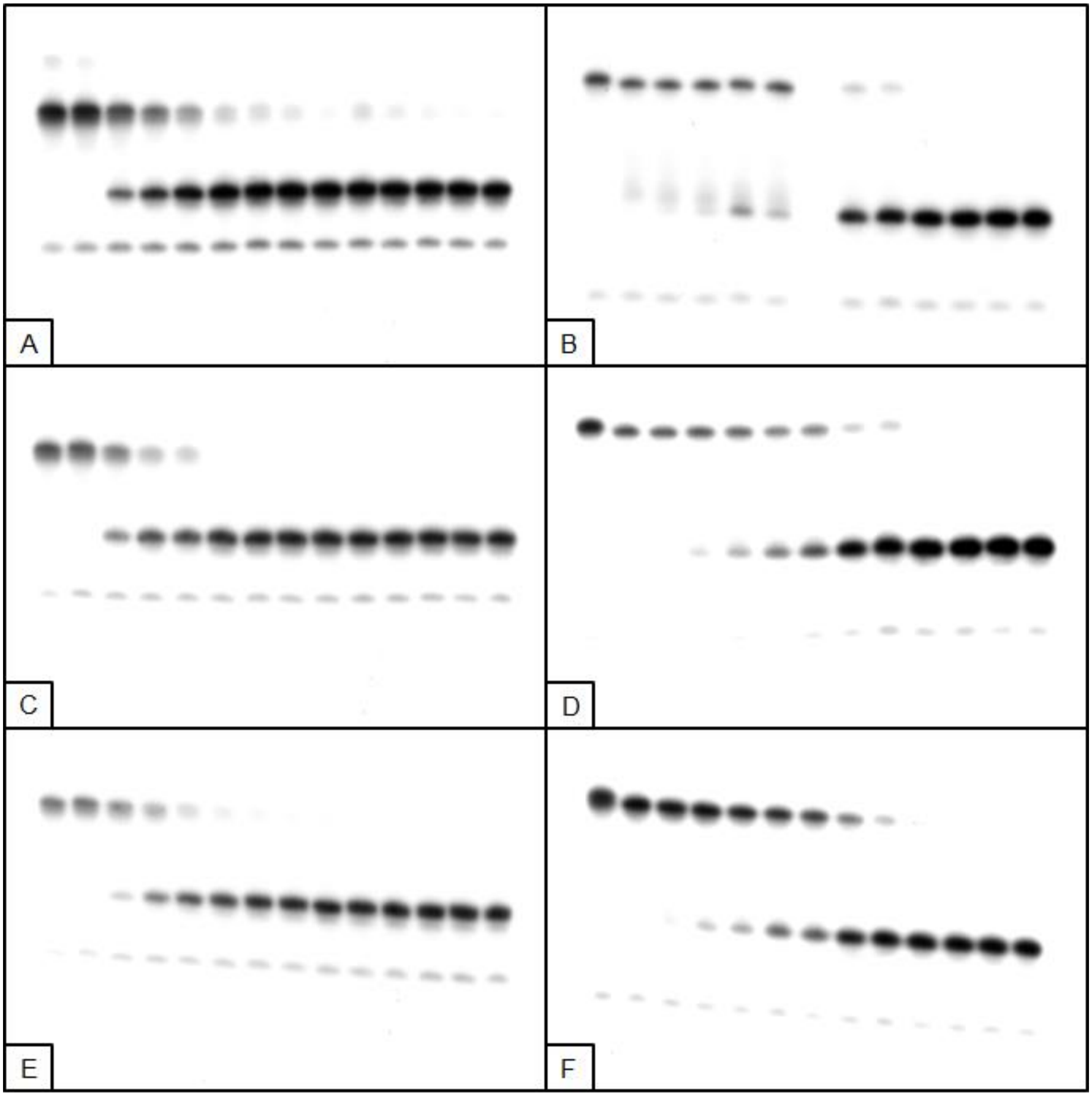

Figure S40. Cleavage assay of dU(+5)RcdG by 0.02U UDG and 0.5U hAPE1 (A,C,E) or 0.5U UDG and 0.02U hAPE1 (B,D,F). (A), (C) and (E) show 14 lanes each, which correspond to reaction times 0, 1, 5, 10, 15, 20, 25, 30, 35, 40, 45, 50, 55, and 60 min starting from the left. (B), (D) and (F) show 13 lanes each, which correspond to reaction times 0, 1, 5, 10, 15, 20, 25, 30, 60, 90, 120, 150 and 180 min starting from the left.

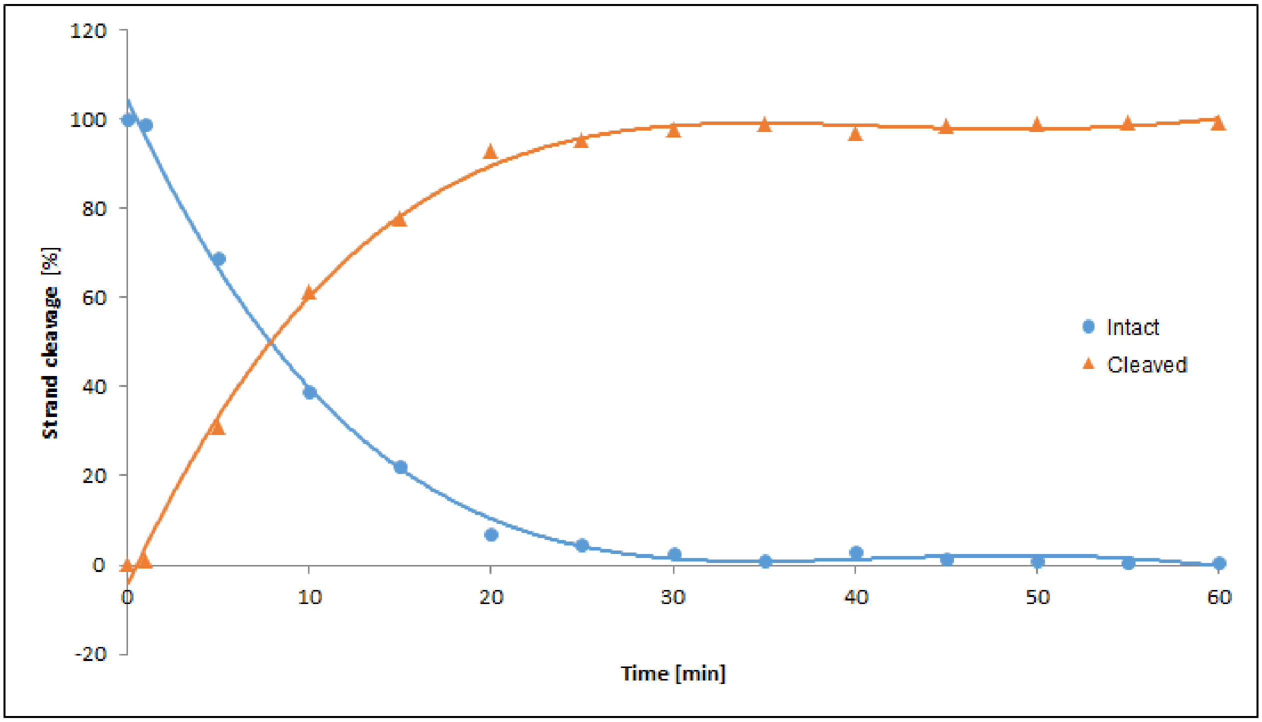

Figure S41. Cleavage assay of dU(+5)RcdG by 0.02U UDG and 0.5U hAPE1. The quantity loss of intact ssDNA (blue) and the quantity increase of SSB-DNA (orange).

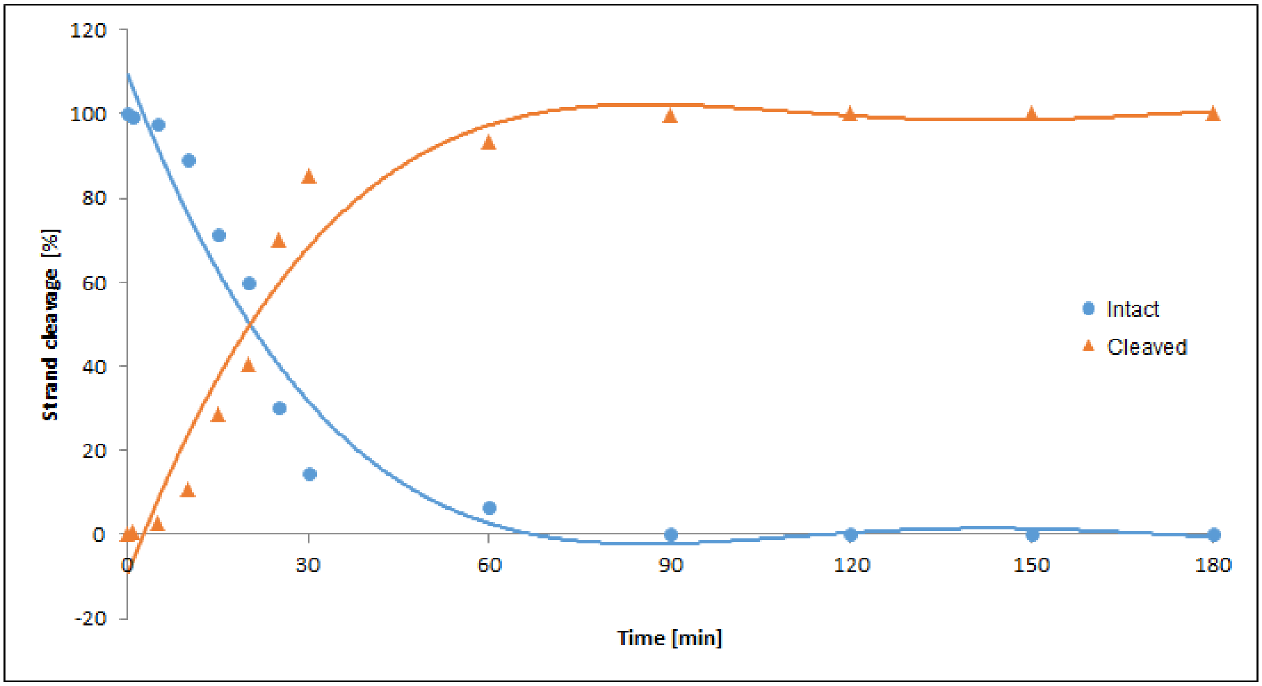

Figure S42. Cleavage assay of dU(+5)RcdG by 0.5U UDG and 0.02U hAPE1. The quantity loss of intact ssDNA (blue) and the quantity increase of SSB-DNA (orange).

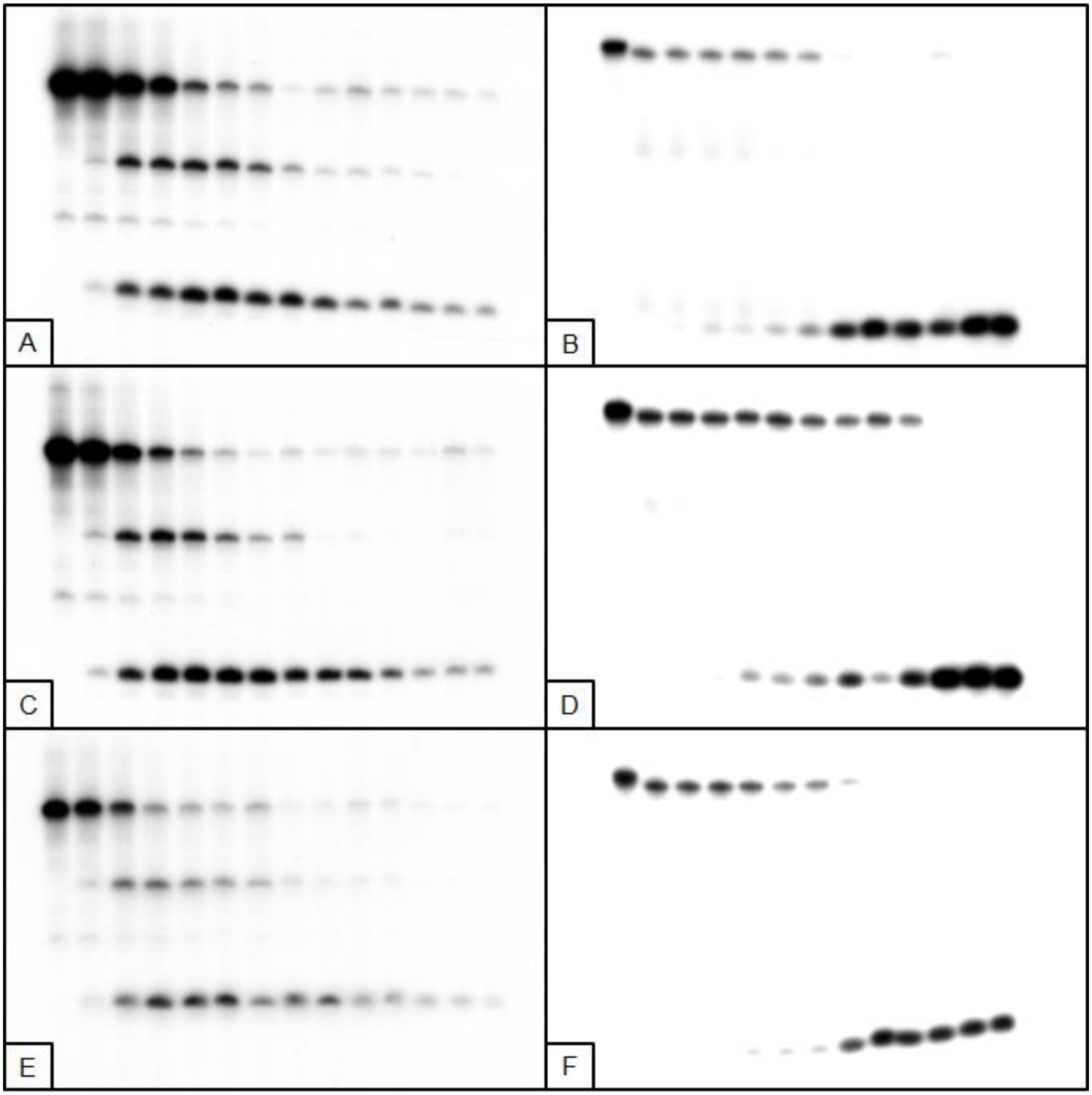

Figure S43. Cleavage assay of dU(-5)(+5)RcdG by 0.02U UDG and 0.5U hAPE1 (A,C,E) or 0.5U UDG and 0.02U hAPE1 (B,D,F) . (A), (C) and (E) show 14 lanes each, which correspond to reaction times 0, 1, 5, 10, 15, 20, 25, 30, 35, 40, 45, 50, 55, and 60 min starting from the left. (B), (D) and (F) show 13 lanes each, which correspond to reaction times 0, 1, 5, 10, 15, 20, 25, 30, 60, 90, 120, 150 and 180 min starting from the left.

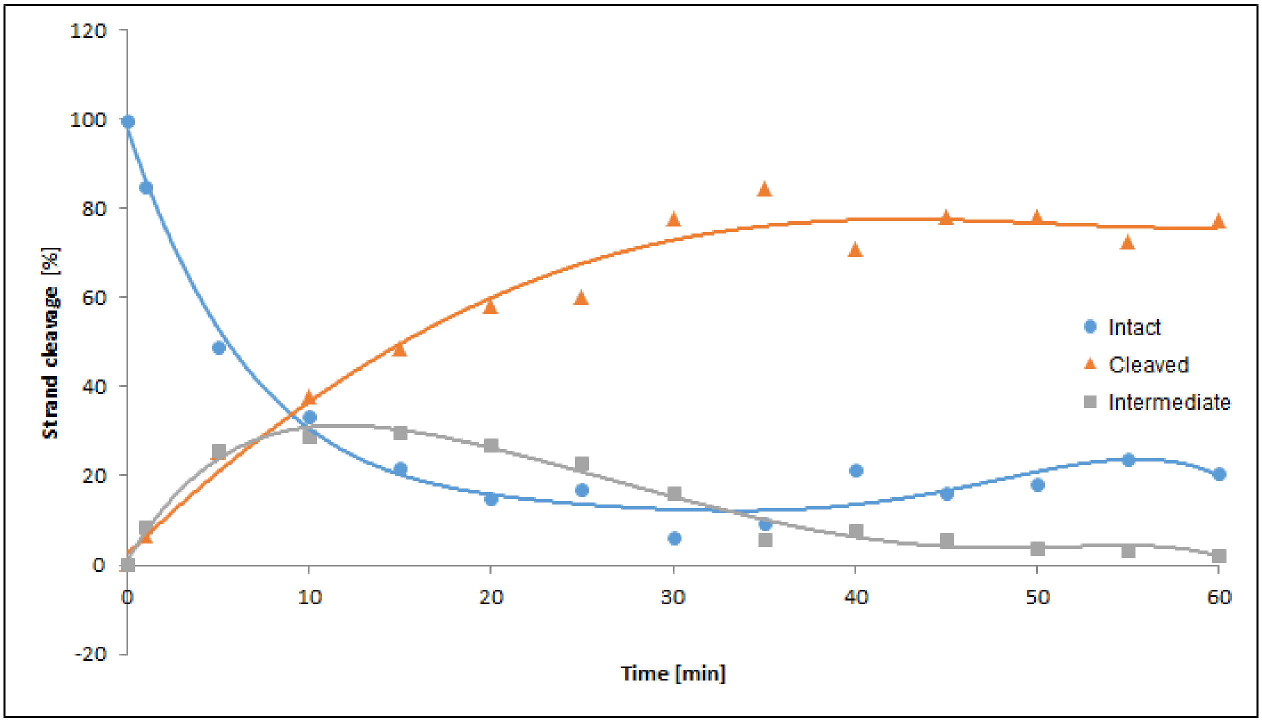

Figure S44. Cleavage assay of dU(-5)(+5)RcdG by 0.02U UDG and 0.5U hAPE1. The quantity loss of intact ssDNA (blue), the quantity increase of SSB-DNA (orange) and an intermediate oligo fragment (grey).

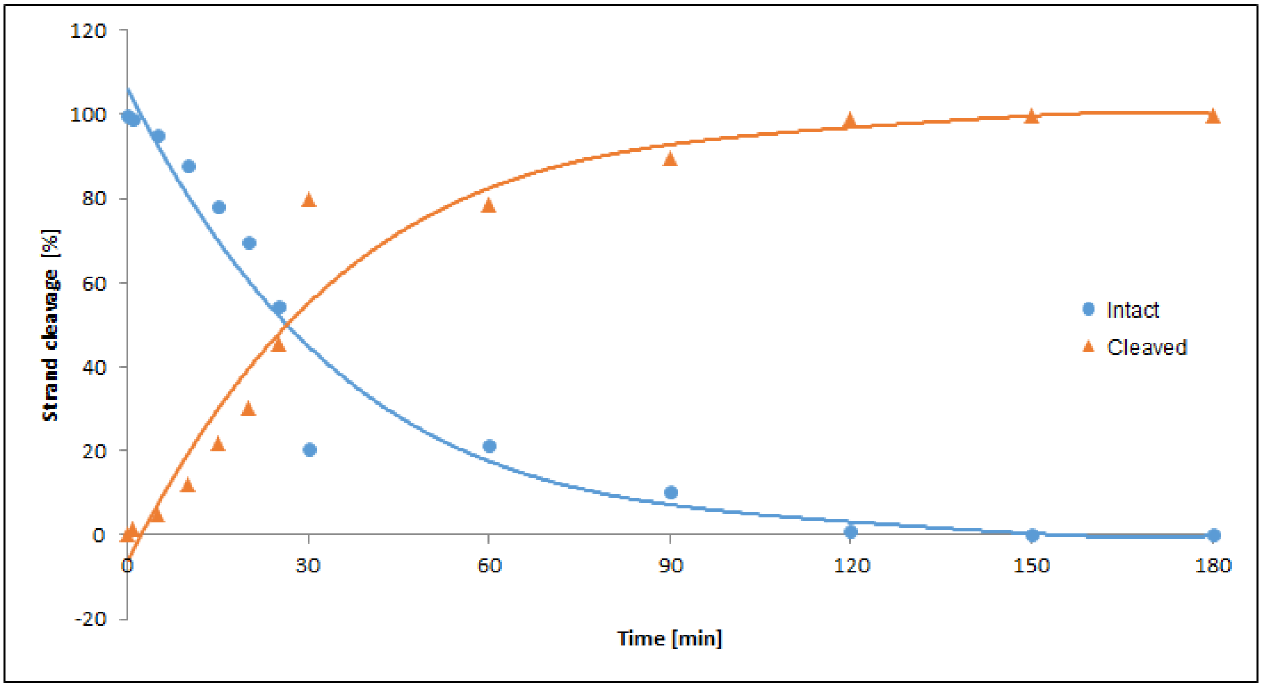

Figure S45. Cleavage assay of dU(-5)(+5)RcdG by 0.5U UDG and 0.02U hAPE1. The quantity loss of intact ssDNA (blue) and the quantity increase of SSB-DNA (orange).

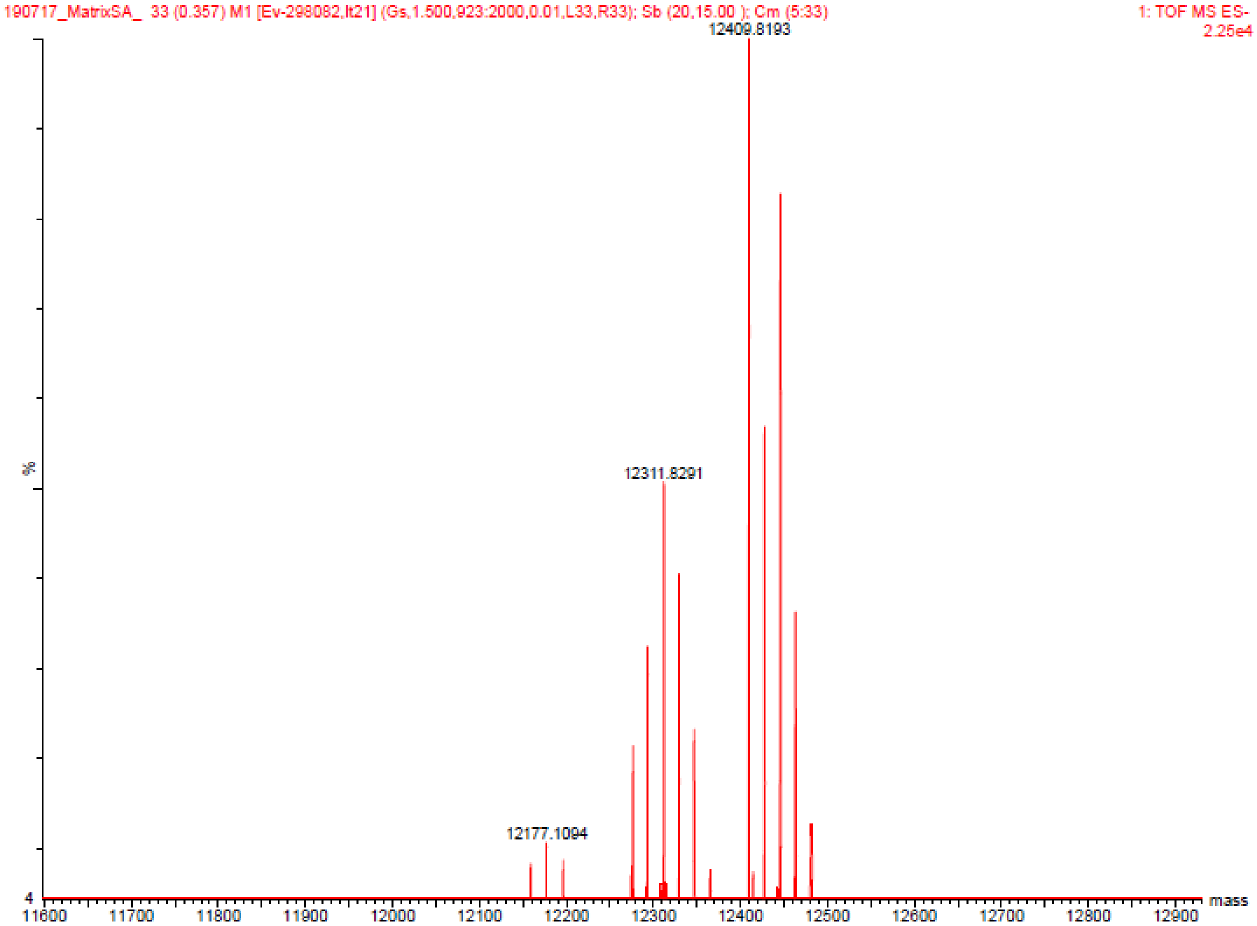

Figure S46. Mass spectrum of Matrix SA.

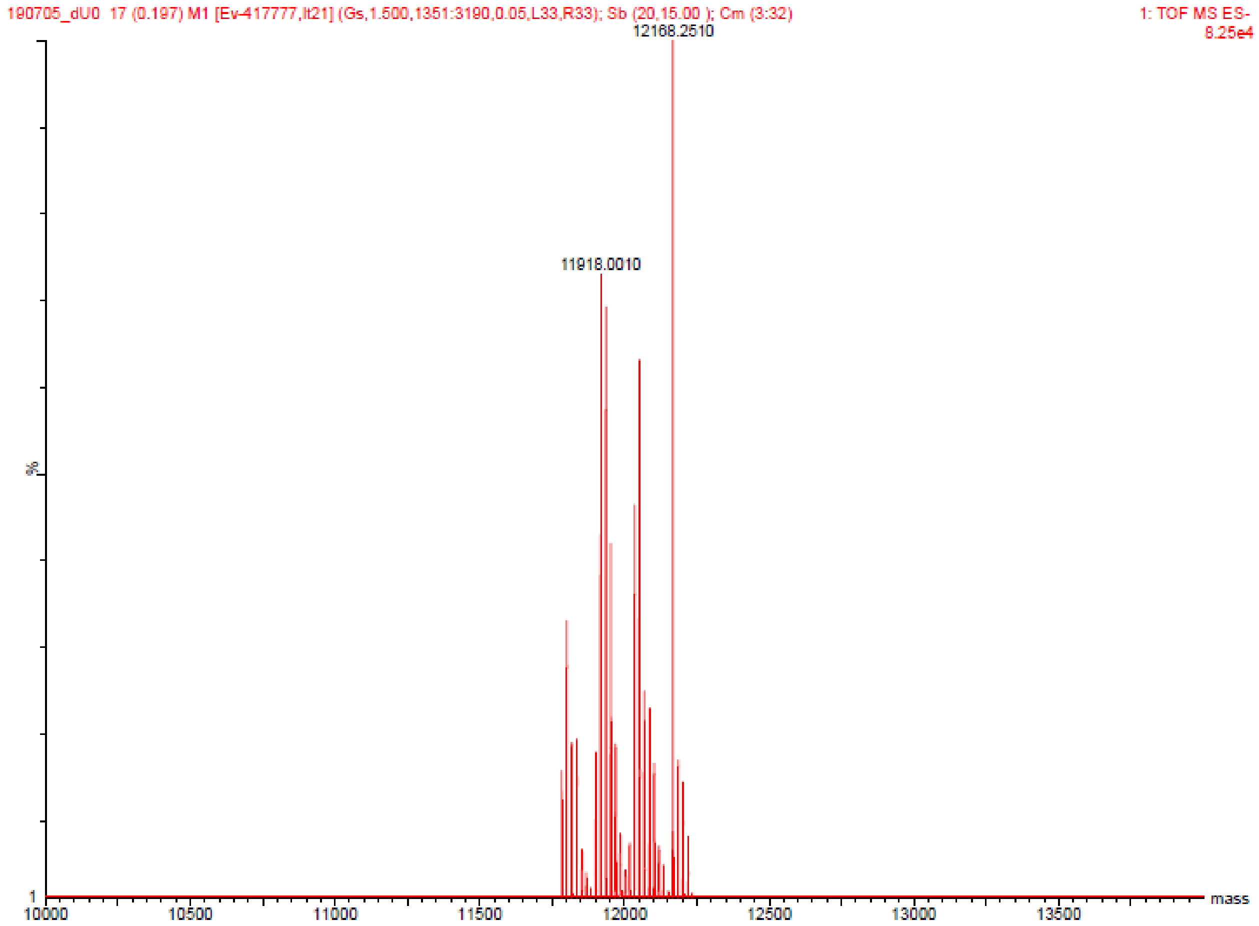

Figure S47. Mass spectrum of dU0.

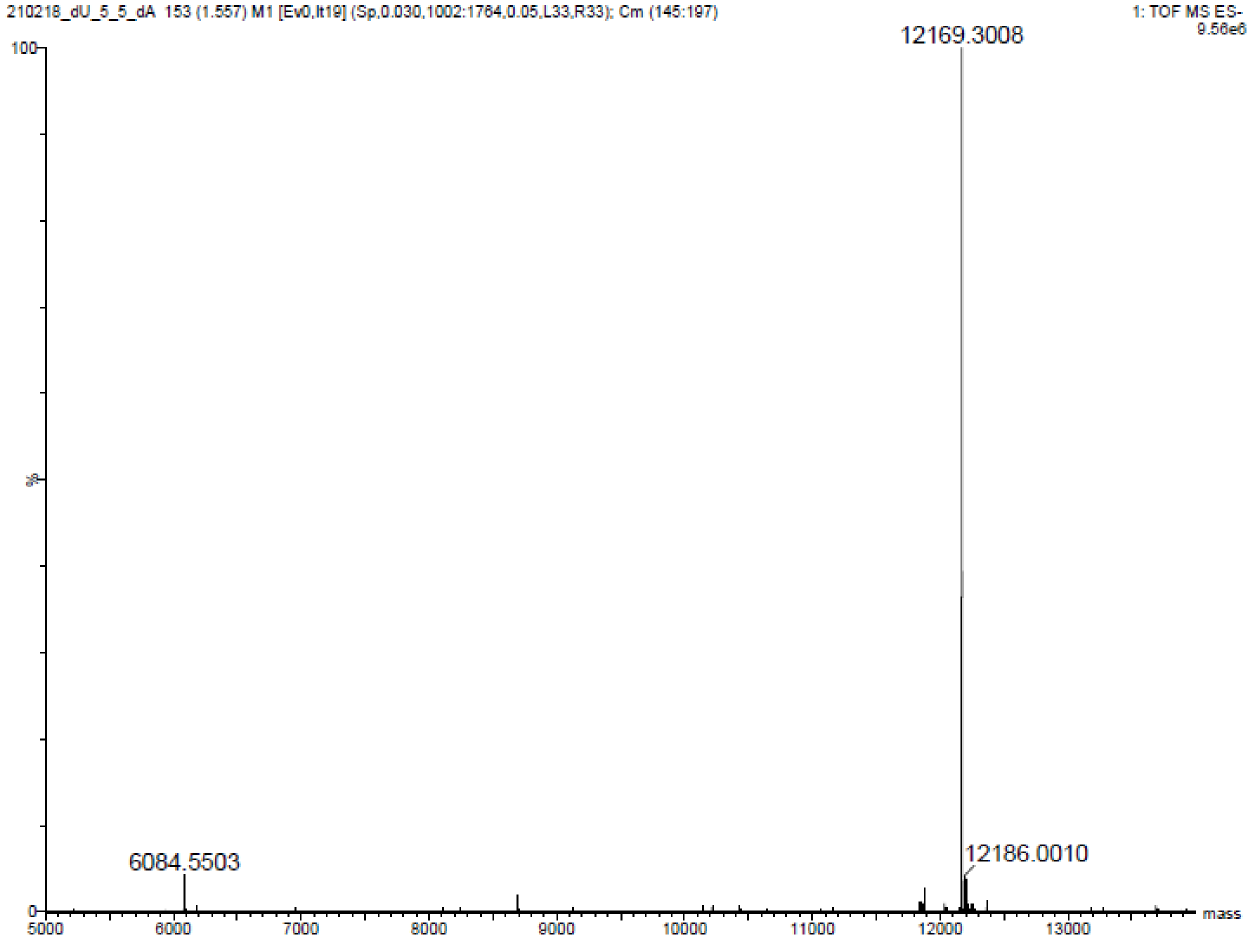

Figure S48. Mass spectrum of dU(-5)(+5)dA.

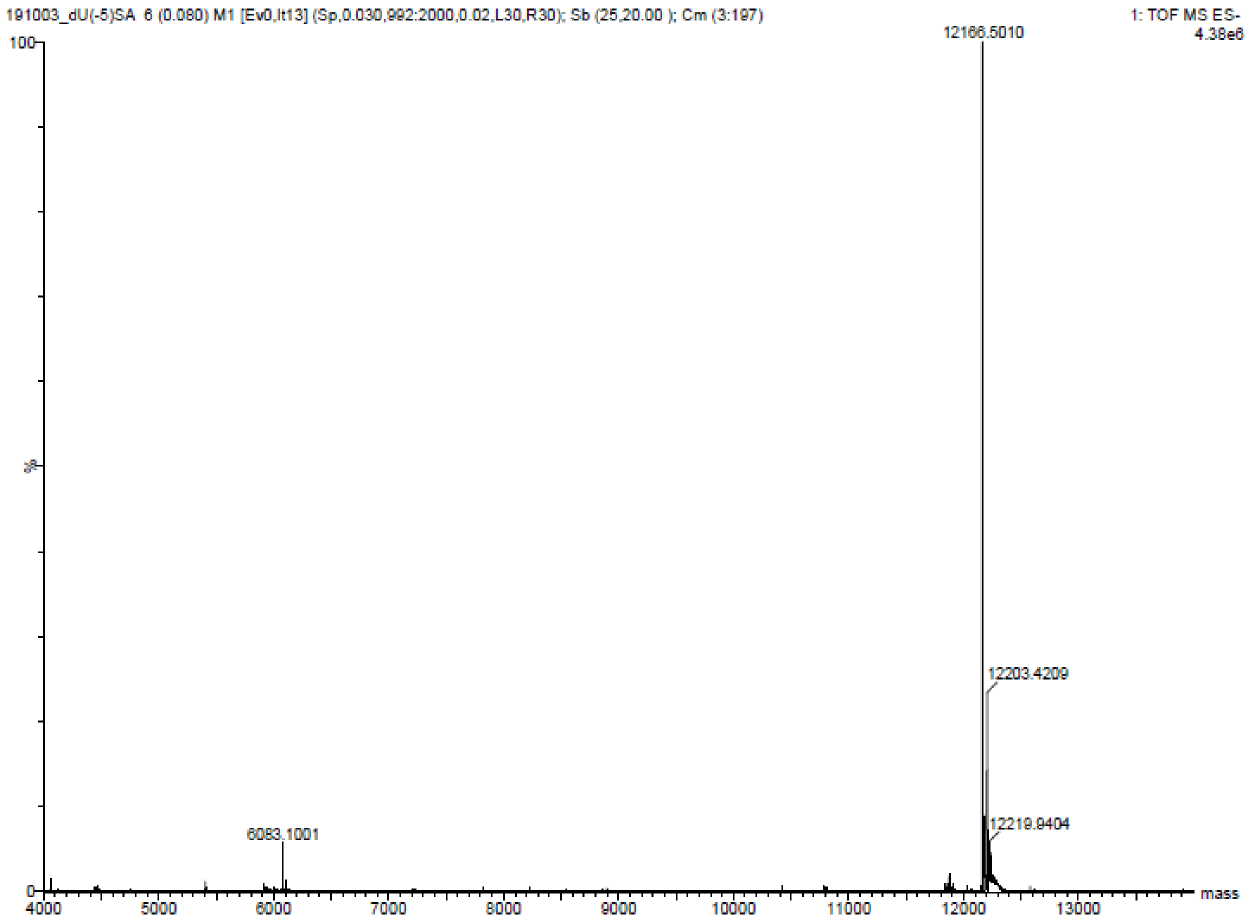

Figure S49. Mass spectrum of dU(-5)SA.

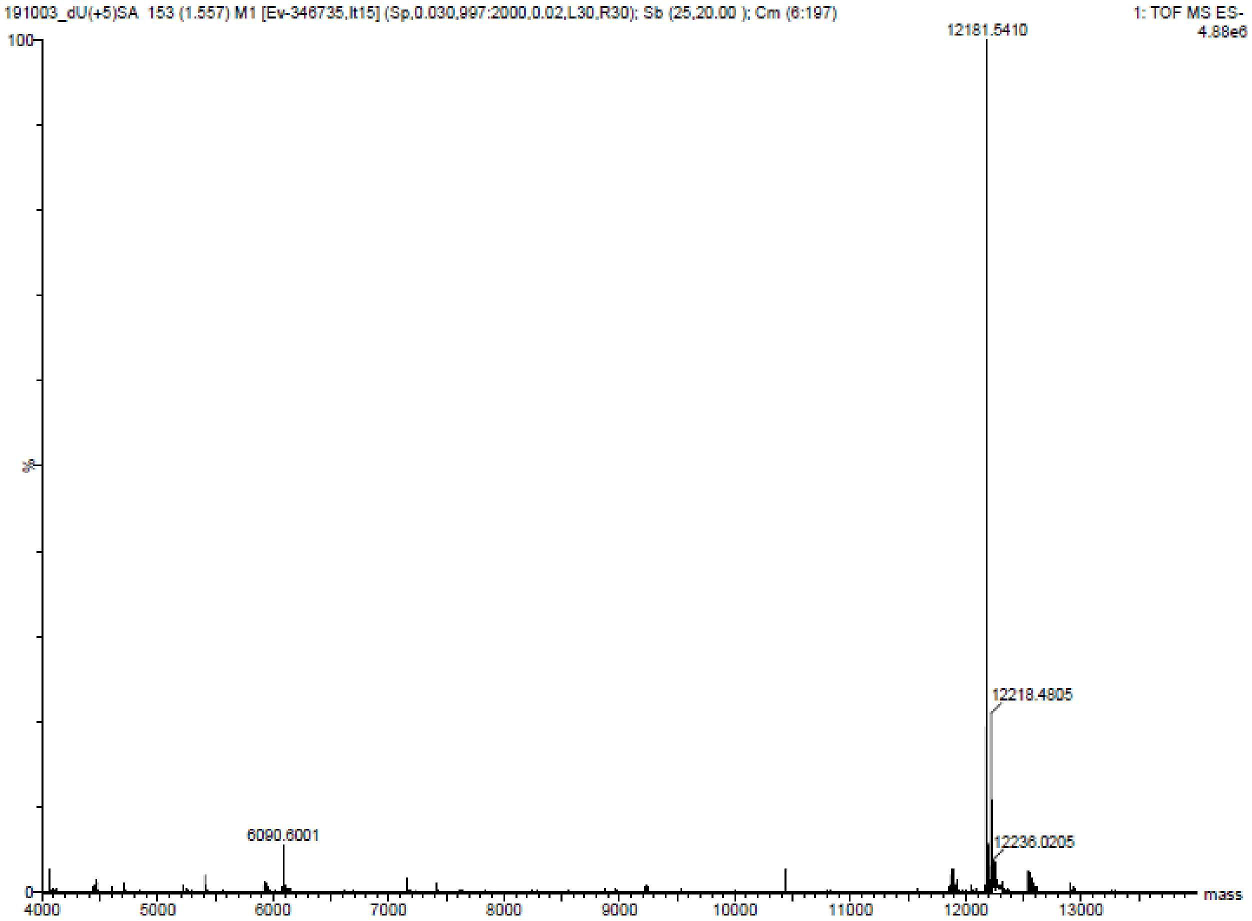

Figure S50. Mass spectrum of dU(+5)SA.

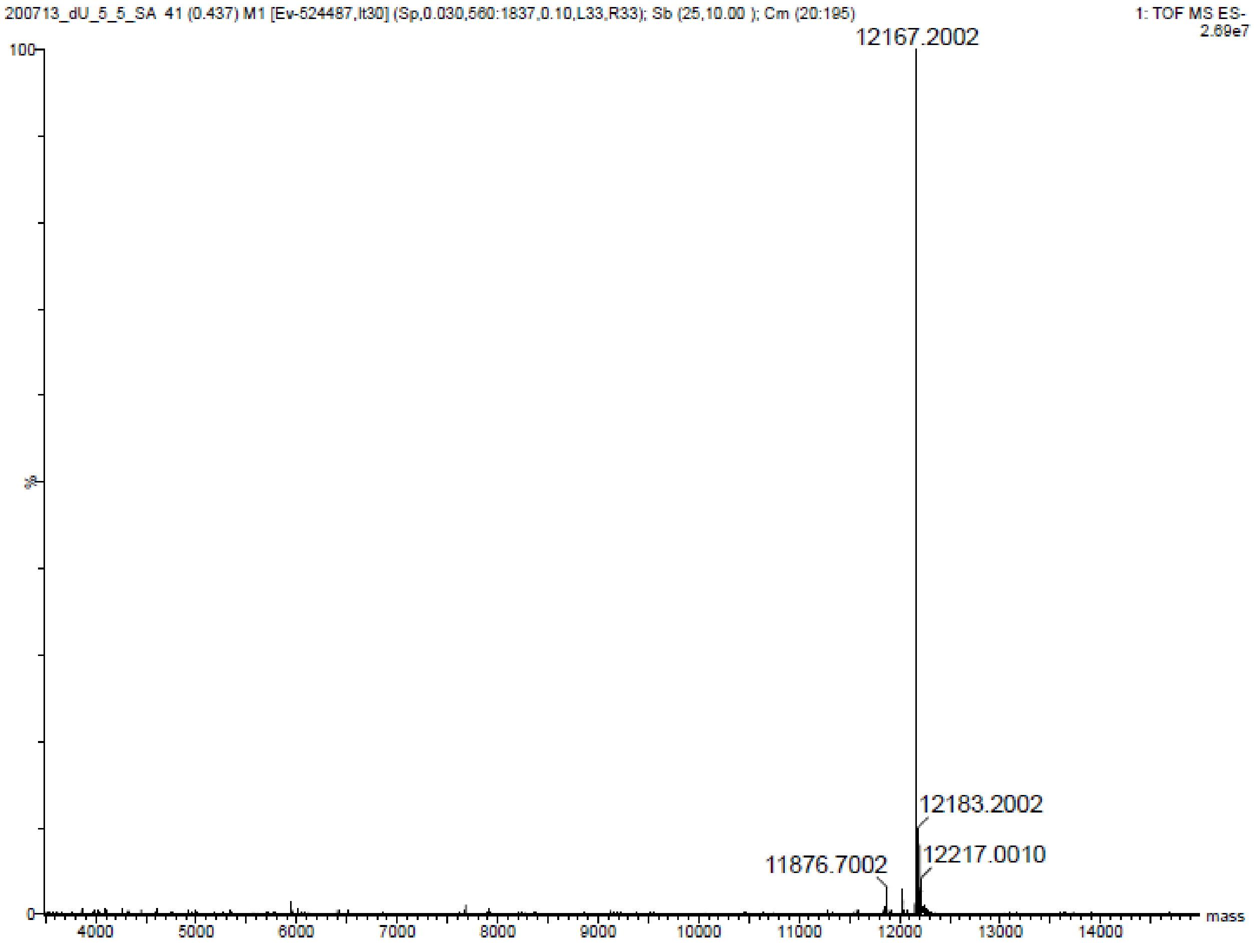

Figure S51. Mass spectrum of dU(-5)(+5)SA.

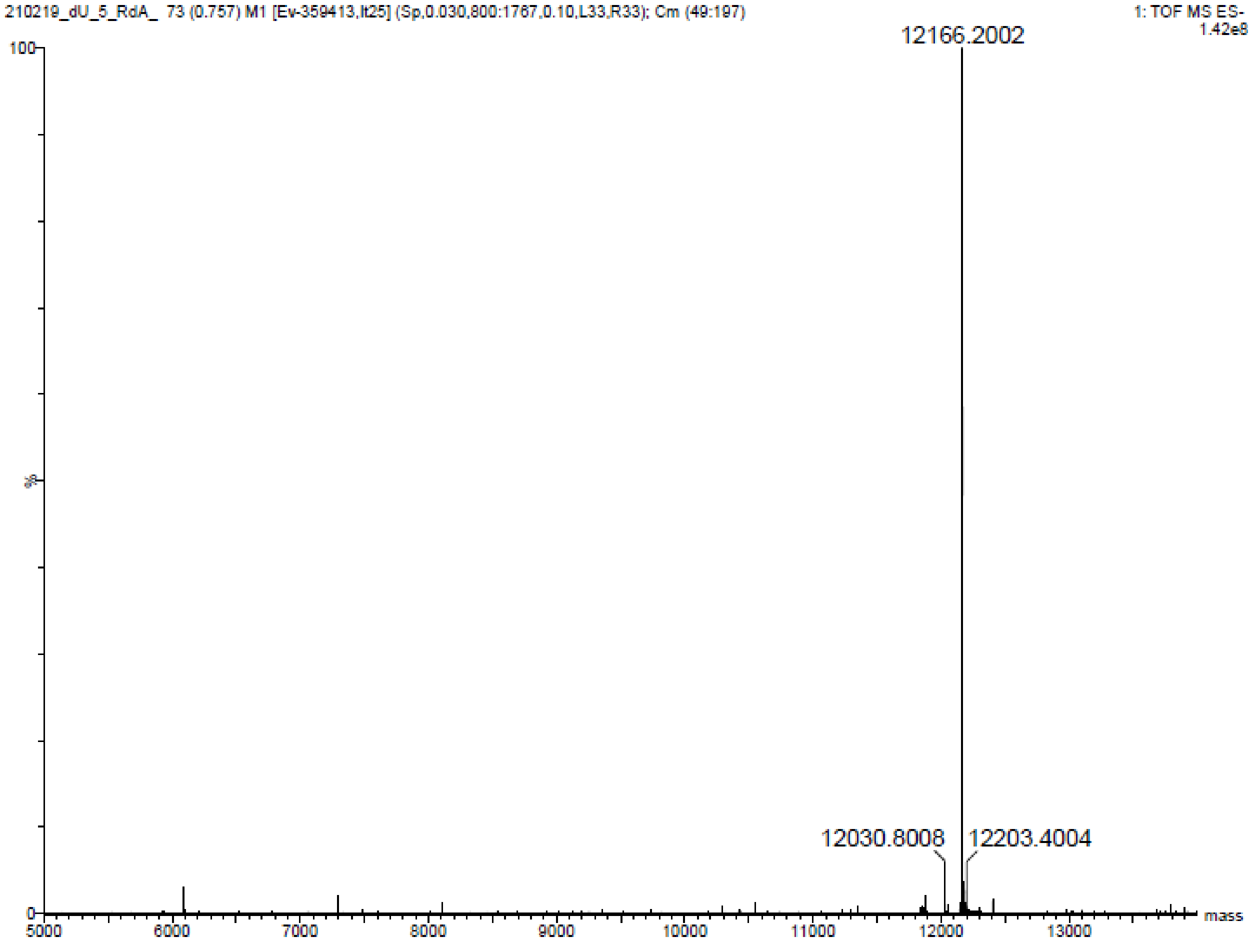

Figure S52. Mass spectrum of dU(-5)RA.

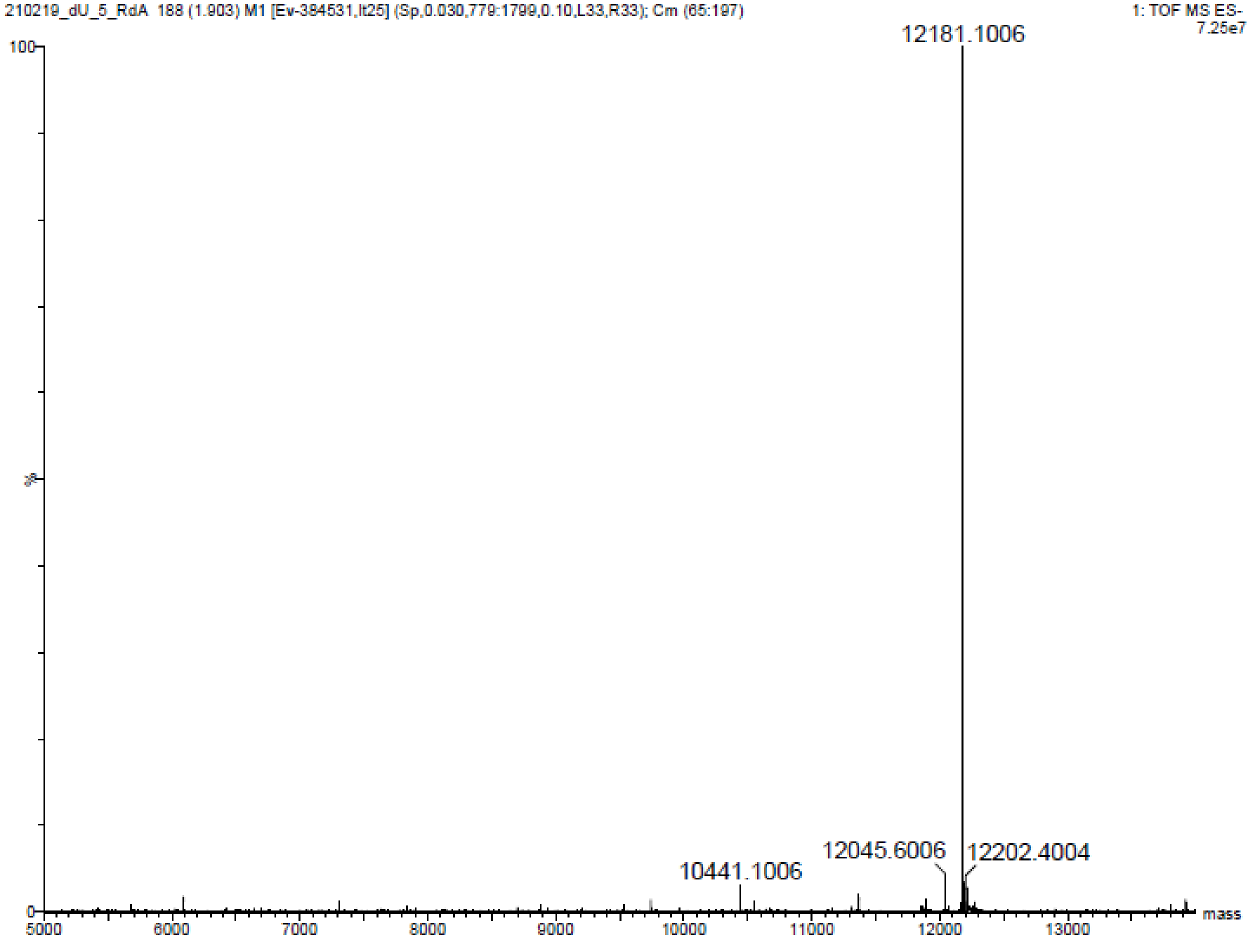

Figure S53. Mass spectrum of dU(+5)RA.

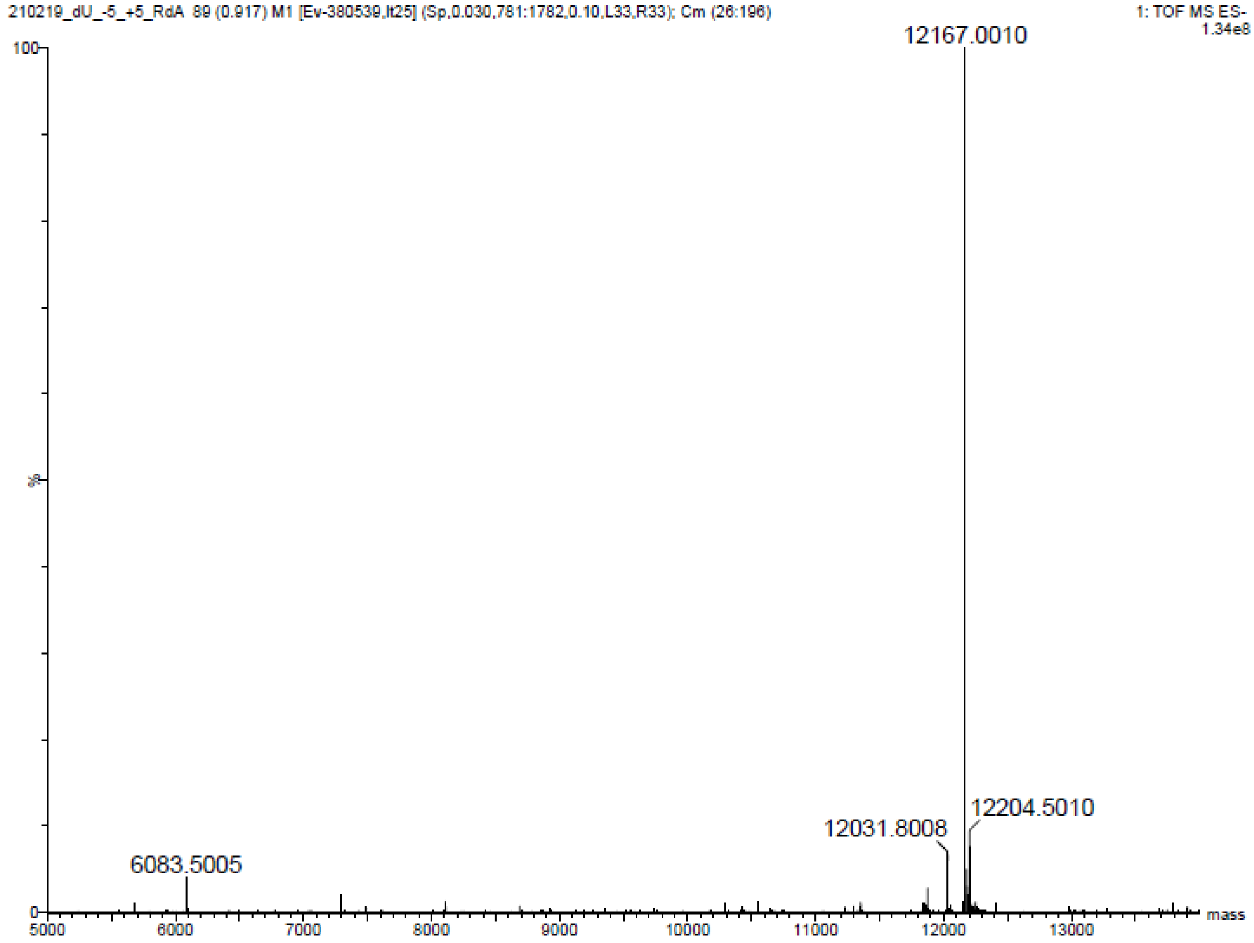

Figure S54. Mass spectrum of dU(-5)(+5)RA.

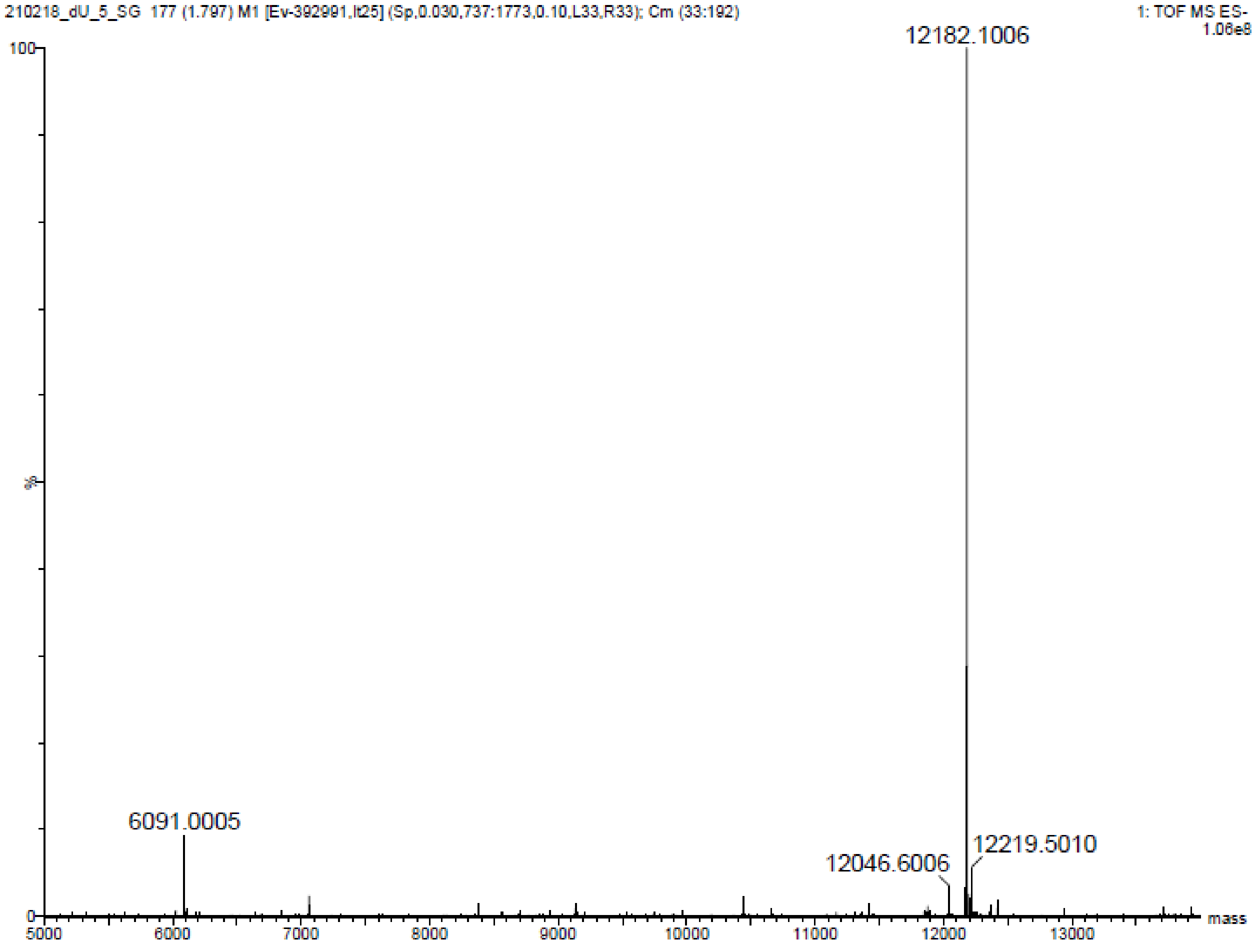

Figure S55. Mass spectrum of dU(-5)SG.

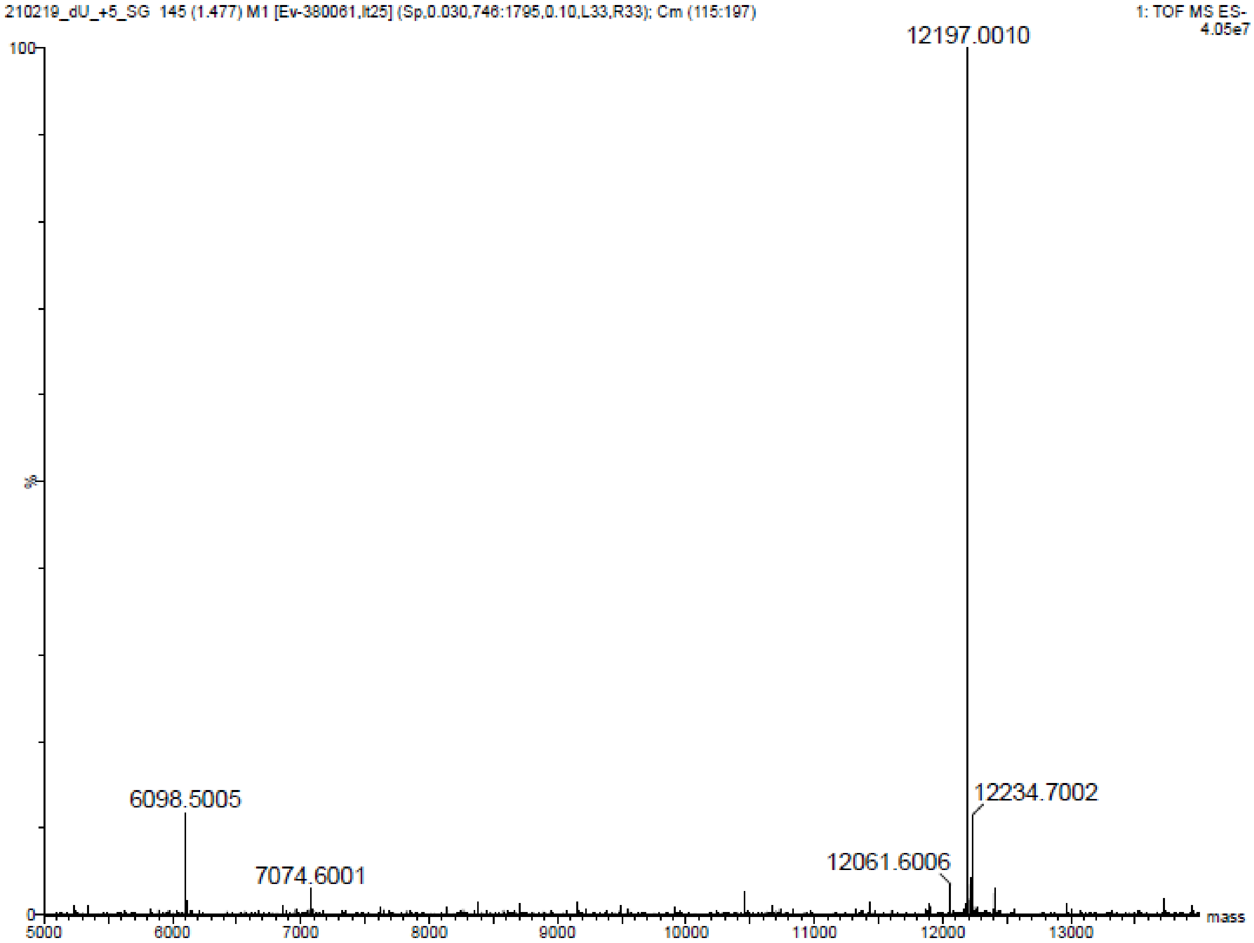

Figure S56. Mass spectrum of dU(+5)SG.

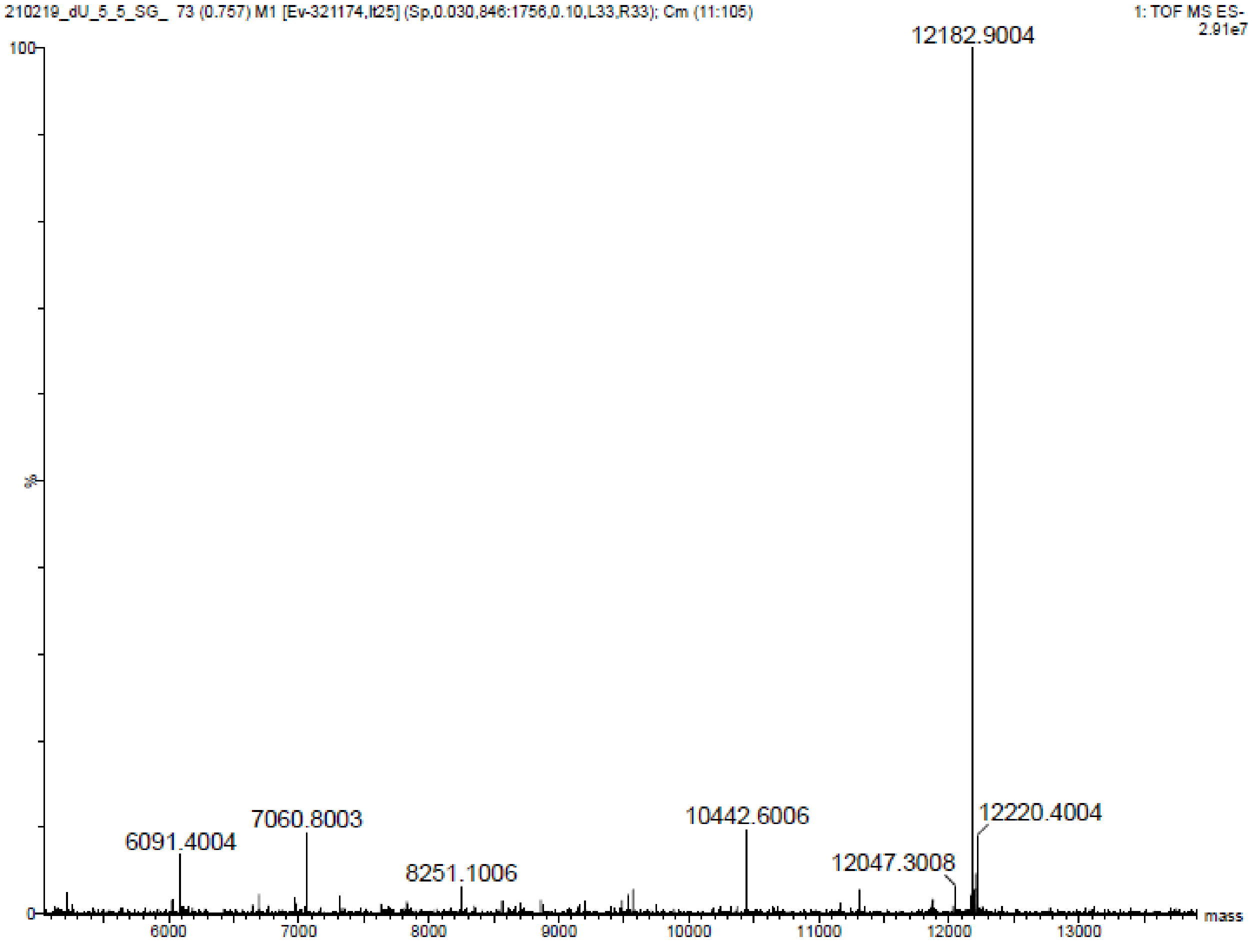

Figure S57. Mass spectrum of dU(-5)(+5)SG.

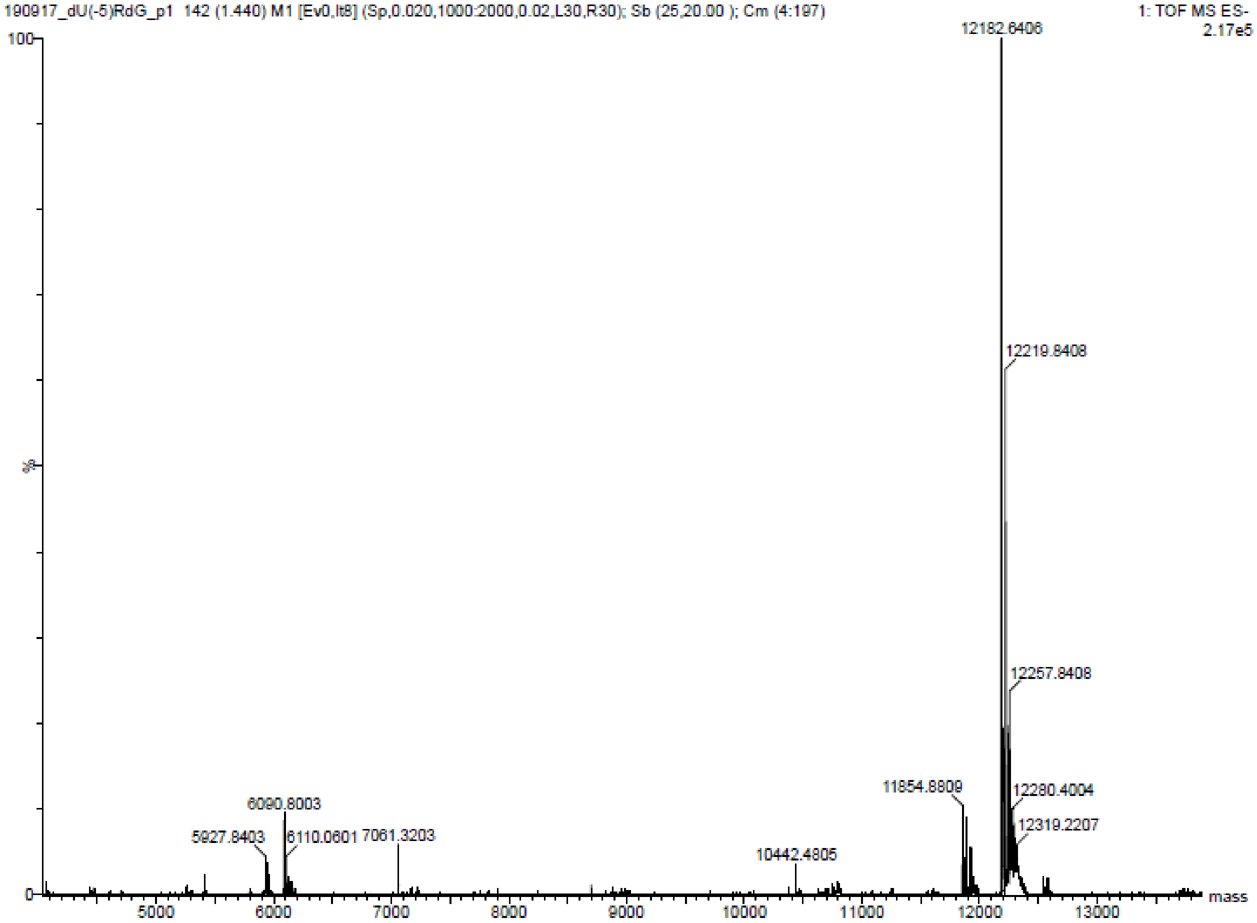

Figure S58. Mass spectrum of dU(-5)RG.

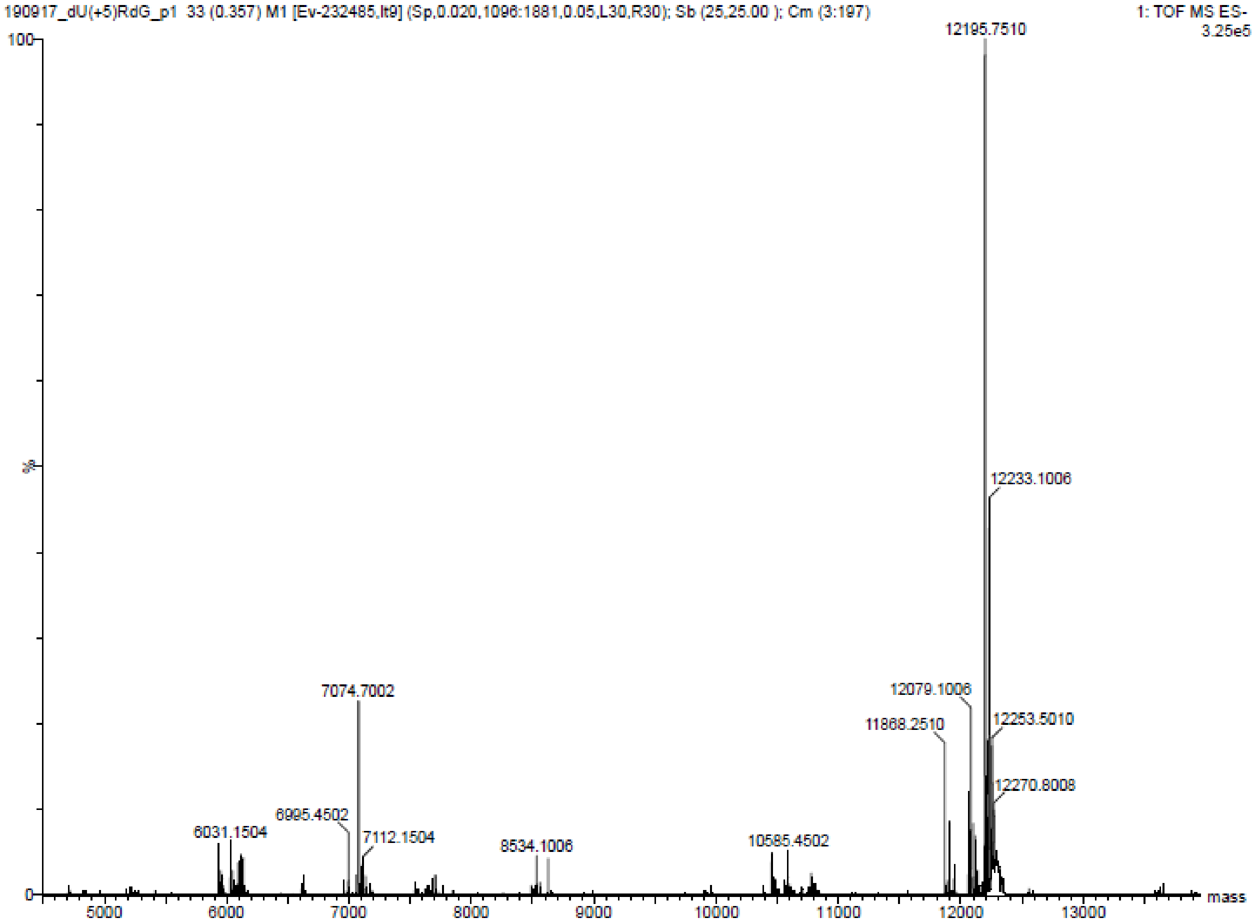

Figure S59. Mass spectrum of dU(+5)RG.

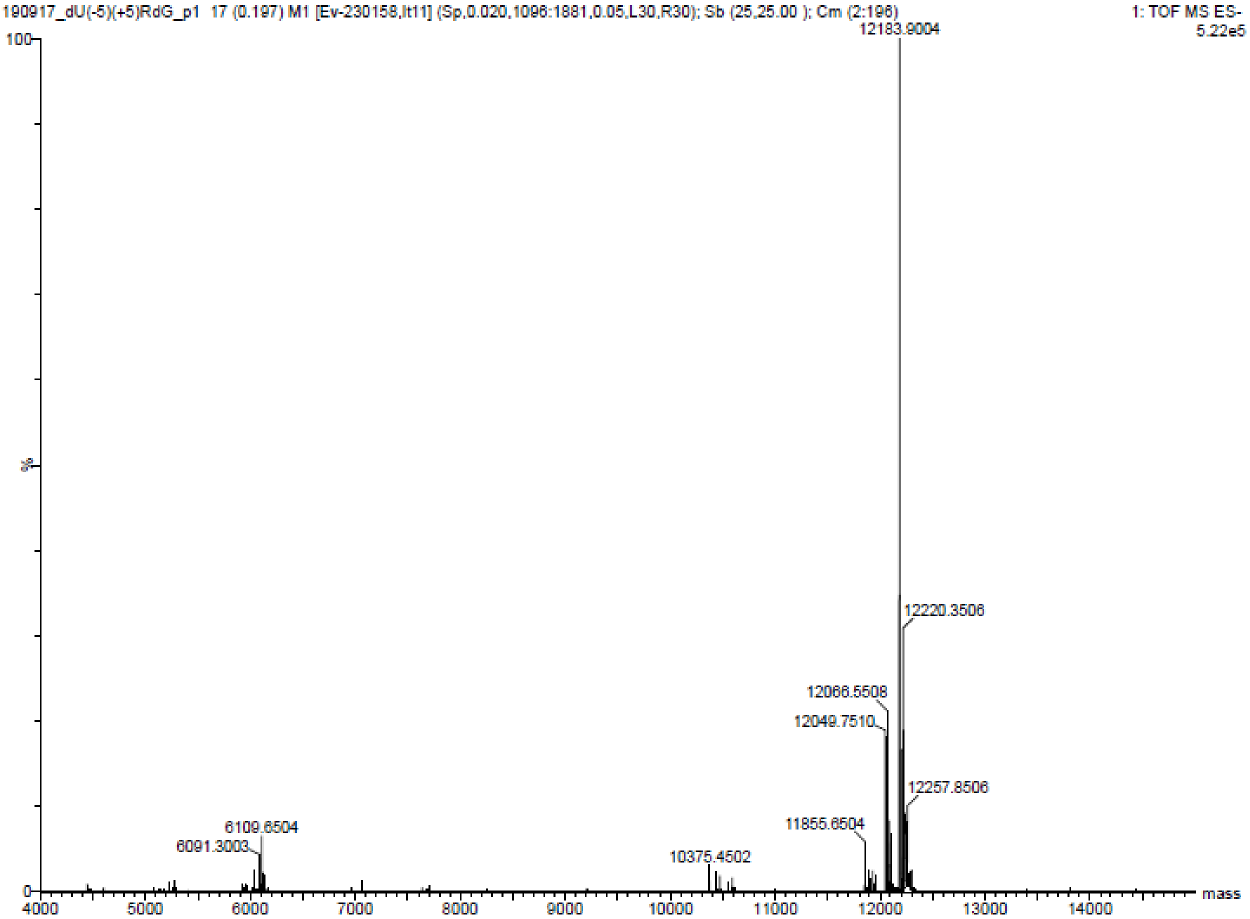

Figure S60. Mass spectrum of dU(-5)(+5)RG.

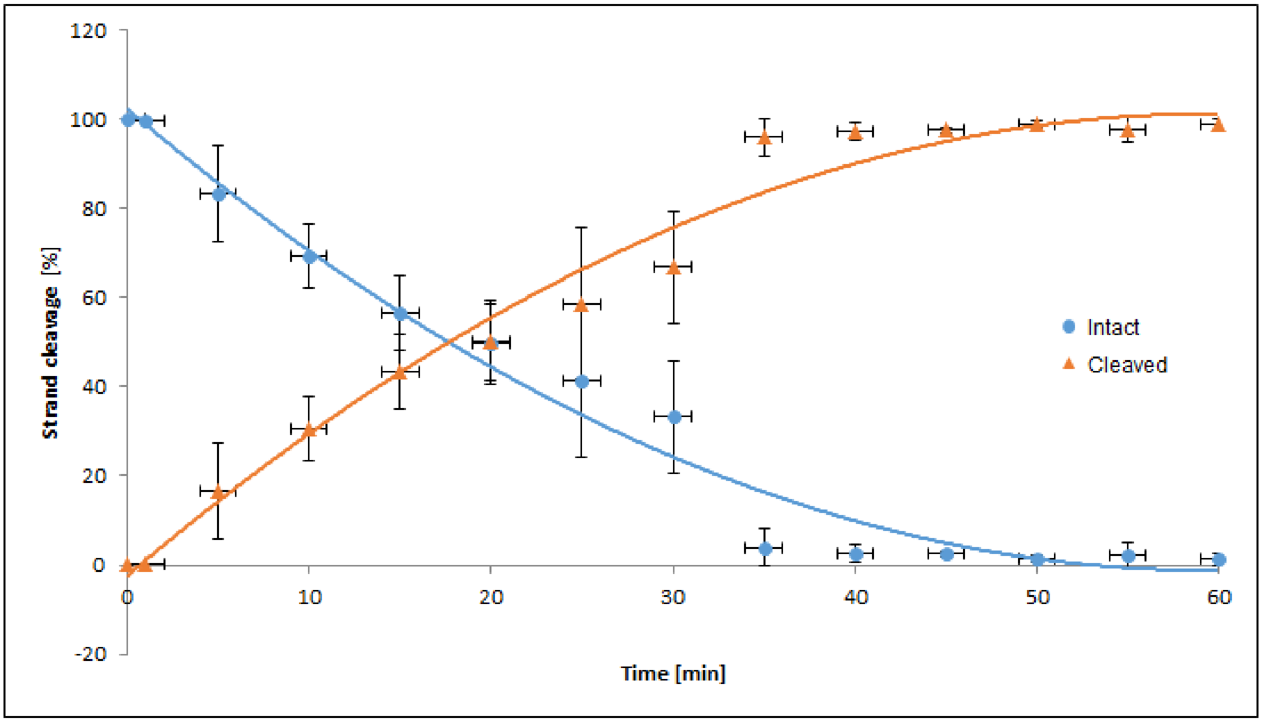

Figure S61. Cleavage assay of dU0 by 0.02U UDG and 0.5U hAPE1. The quantity loss of intact ssDNA (blue) and the quantity increase of SSB-DNA (orange). Error bars are shown.

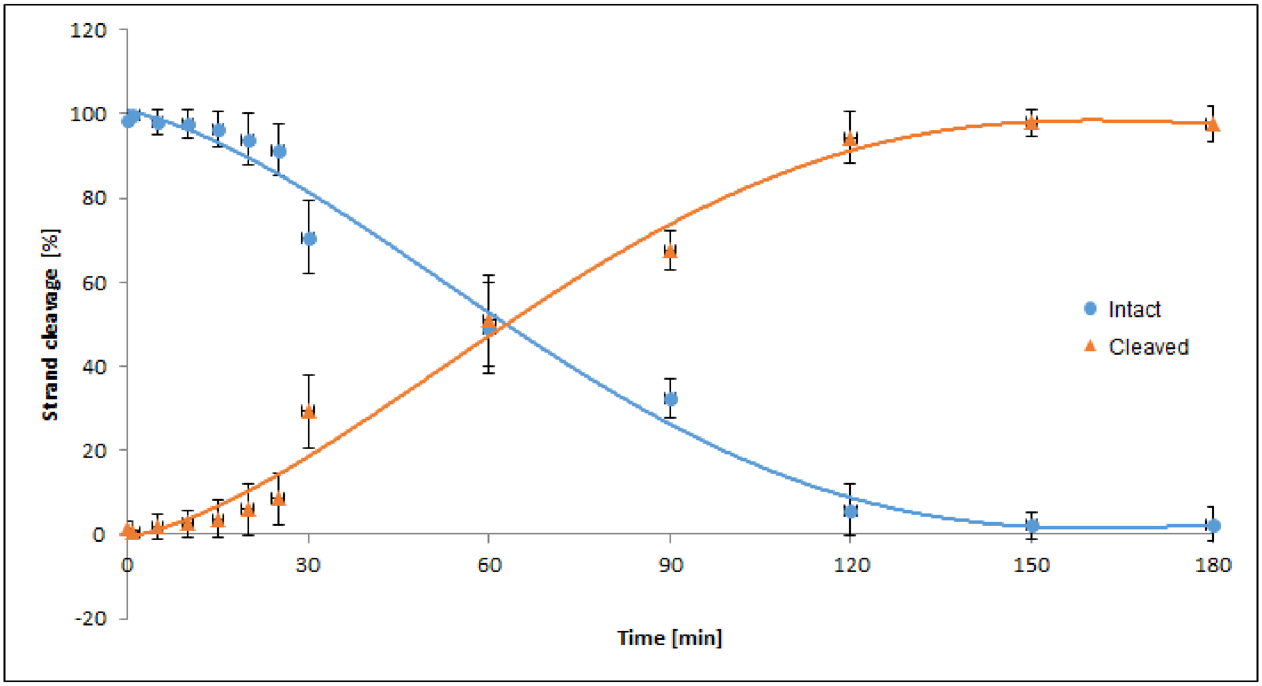

Figure S62. Cleavage assay of dU0 by 0.5U UDG and 0.02U hAPE1. The quantity loss of intact ssDNA (blue) and the quantity increase of SSB-DNA (orange). Error bars are shown.

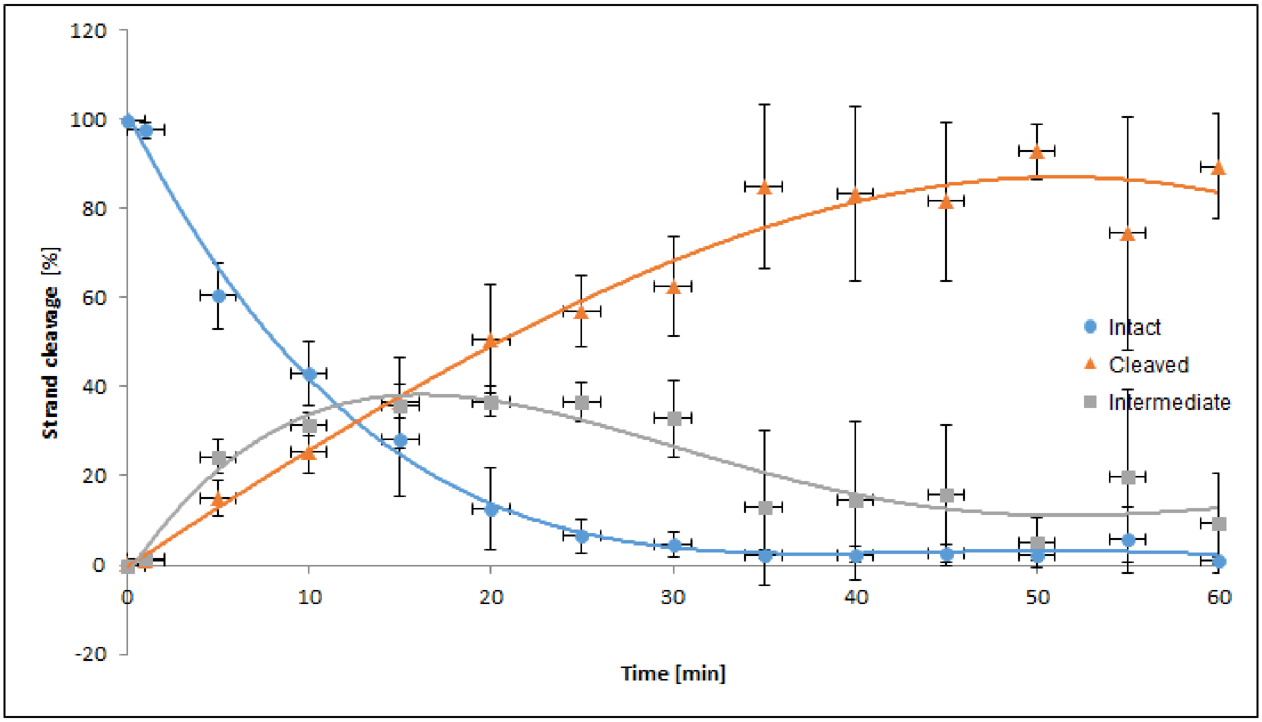

Figure S63. Cleavage assay of dU(-5)(+5)dA by 0.02U UDG and 0.5U hAPE1. The quantity loss of intact ssDNA (blue), the quantity increase of SSB-DNA (orange) and an intermediate oligo fragment (grey). Error bars are shown.

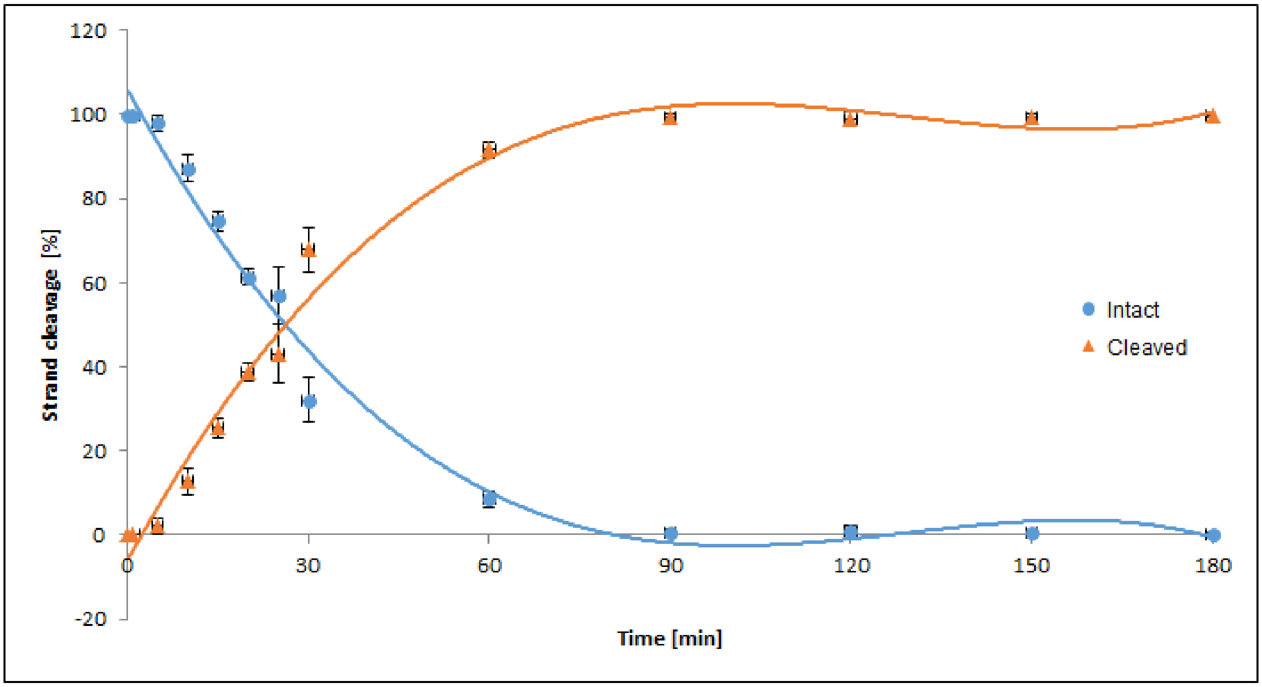

Figure S64. Cleavage assay of dU(-5)(+5)dA by 0.5U UDG and 0.02U hAPE1. The quantity loss of intact ssDNA (blue) and the quantity increase of SSB-DNA (orange). Error bars are shown.

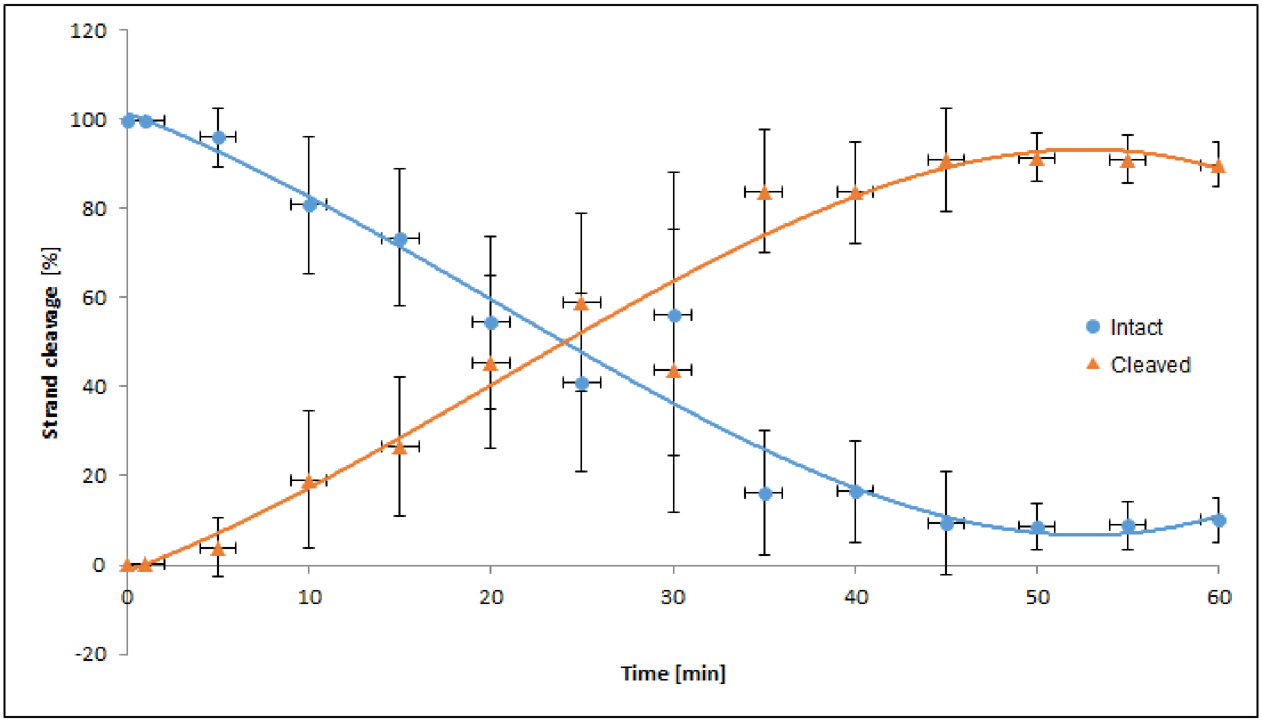

Figure S65. Cleavage assay of dU(-5)ScdA by 0.02U UDG and 0.5U hAPE1. The quantity loss of intact ssDNA (blue) and the quantity increase of SSB-DNA (orange). Error bars are shown.

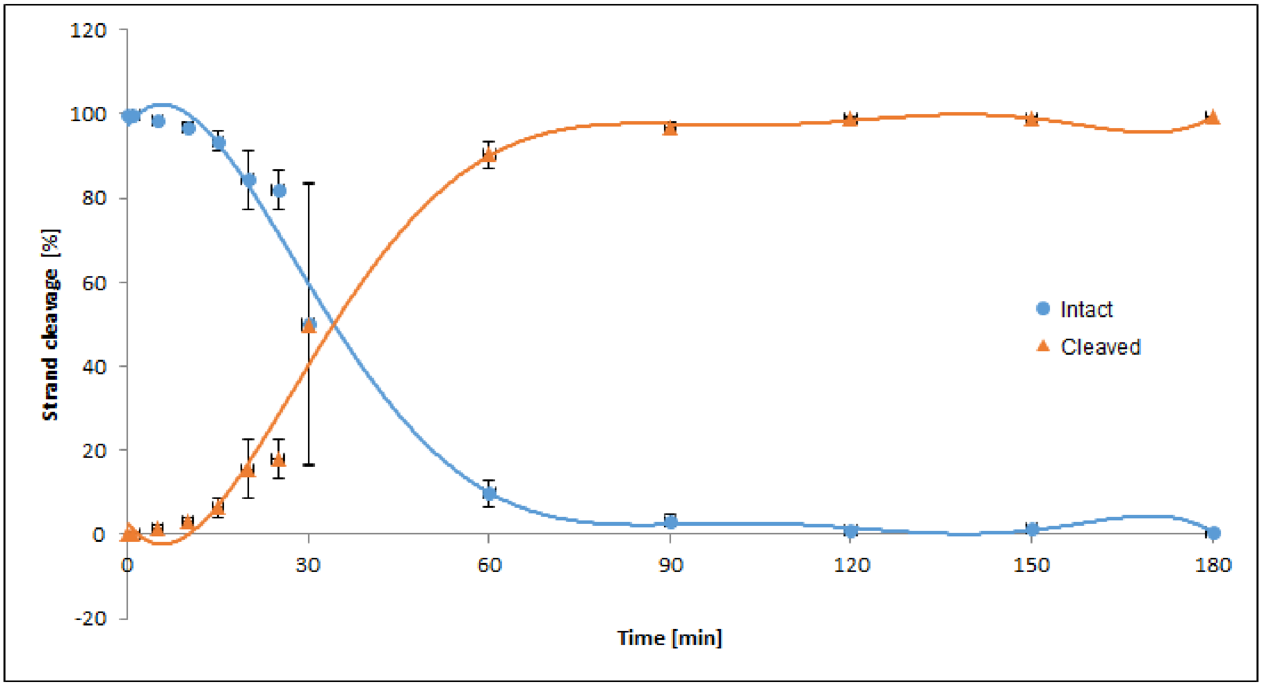

Figure S66. Cleavage assay of dU(-5)ScdA by 0.5U UDG and 0.02U hAPE1. The quantity loss of intact ssDNA (blue) and the quantity increase of SSB-DNA (orange). Error bars are shown.

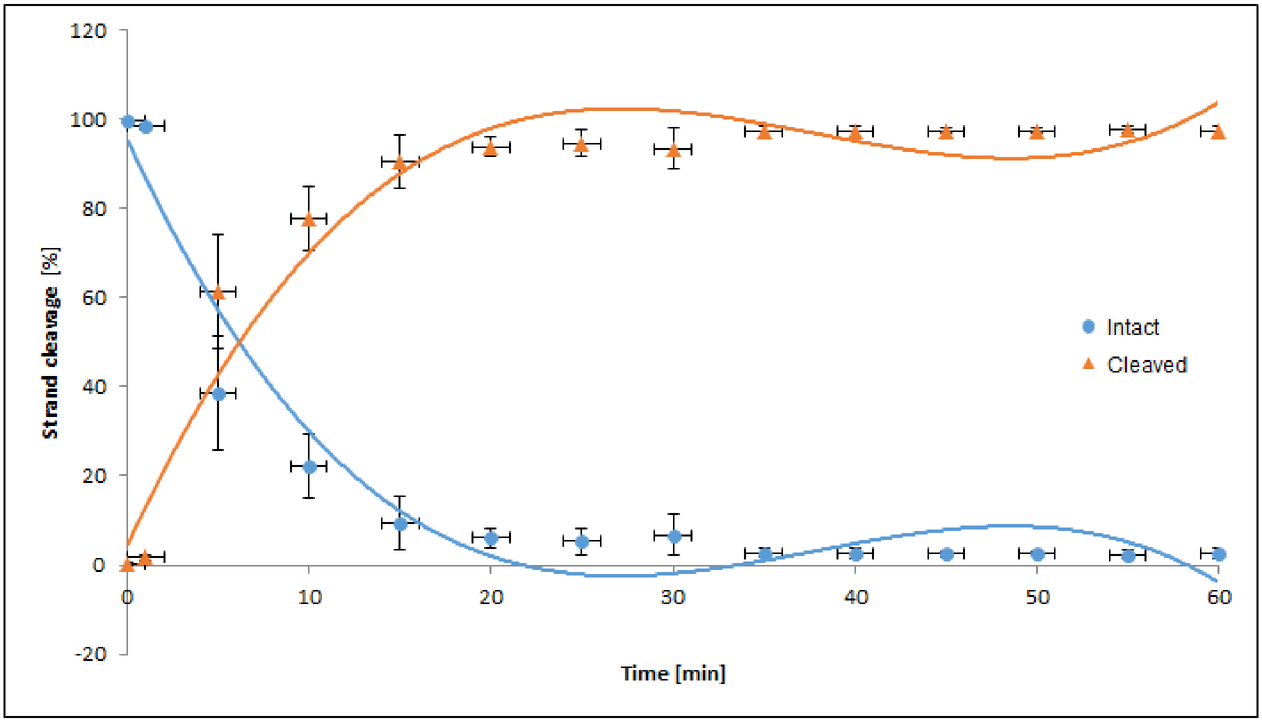

Figure S67. Cleavage assay of dU(+5)ScdA by 0.02U UDG and 0.5U hAPE1. The quantity loss of intact ssDNA (blue) and the quantity increase of SSB-DNA (orange). Error bars are shown.

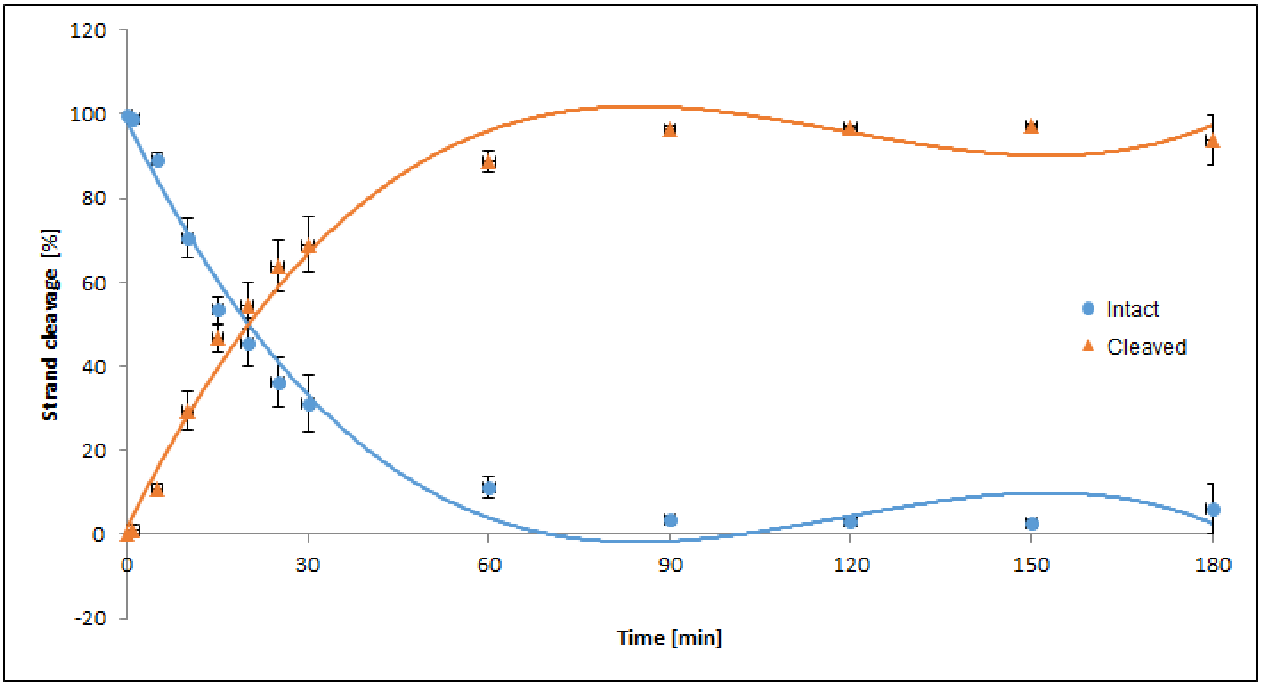

Figure S68. Cleavage assay of dU(+5)ScdA by 0.5U UDG and 0.02U hAPE1. The quantity loss of intact ssDNA (blue) and the quantity increase of SSB-DNA (orange). Error bars are shown.

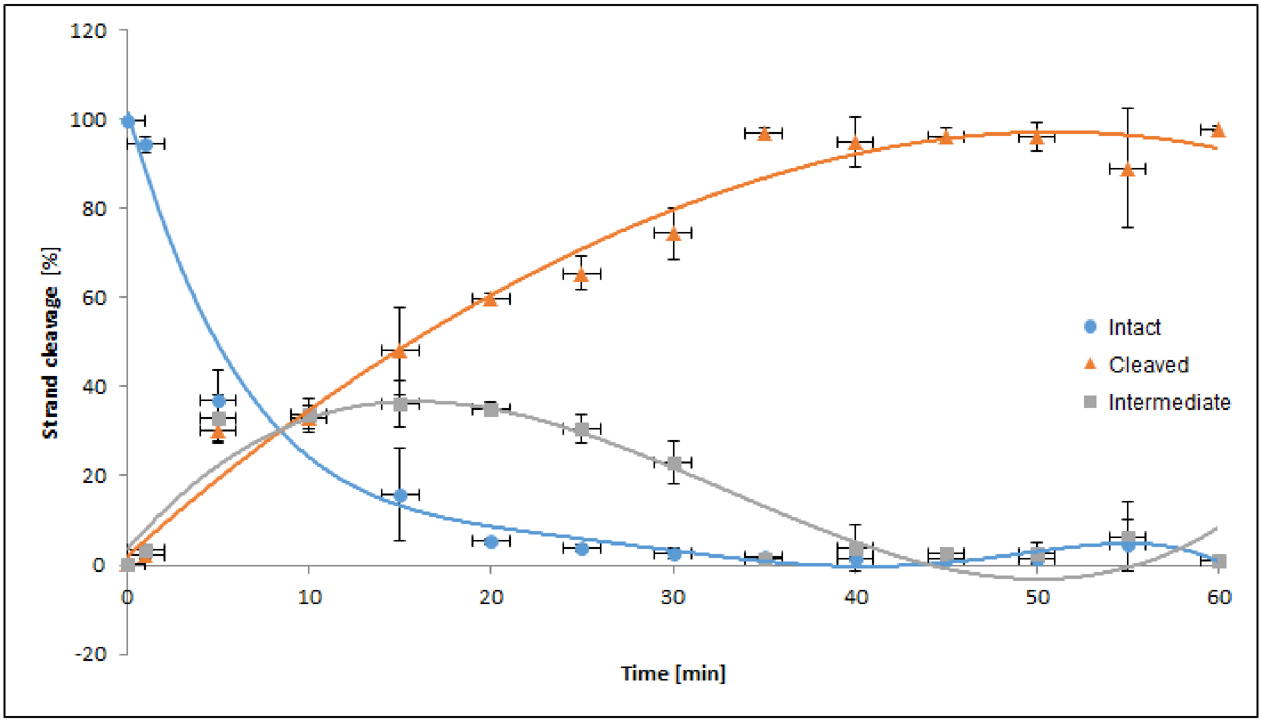

Figure S69. Cleavage assay of dU(-5)(+5)ScdA by 0.02U UDG and 0.5U hAPE1. The quantity loss of intact ssDNA (blue), the quantity increase of SSB-DNA (orange) and an intermediate oligo fragment (grey). Error bars are shown.

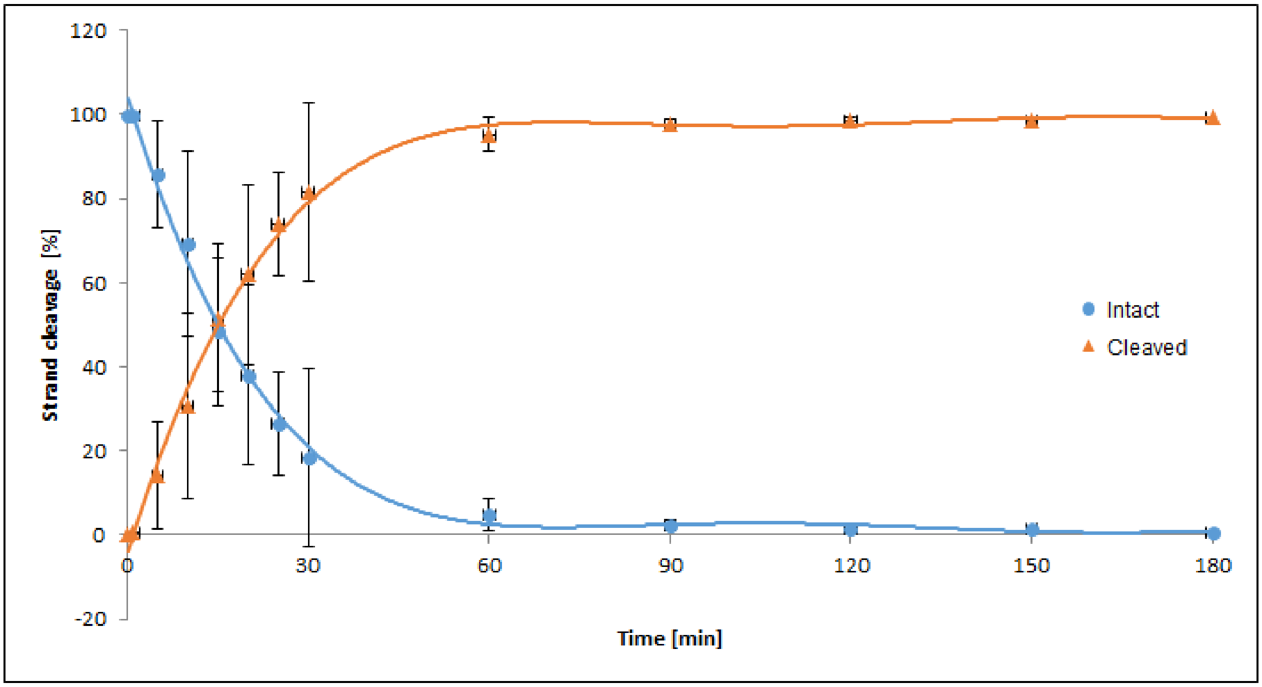

Figure S70. Cleavage assay of dU(-5)(+5)ScdA by 0.5U UDG and 0.02U hAPE1. The quantity loss of intact ssDNA (blue) and the quantity increase of SSB-DNA (orange). Error bars are shown.

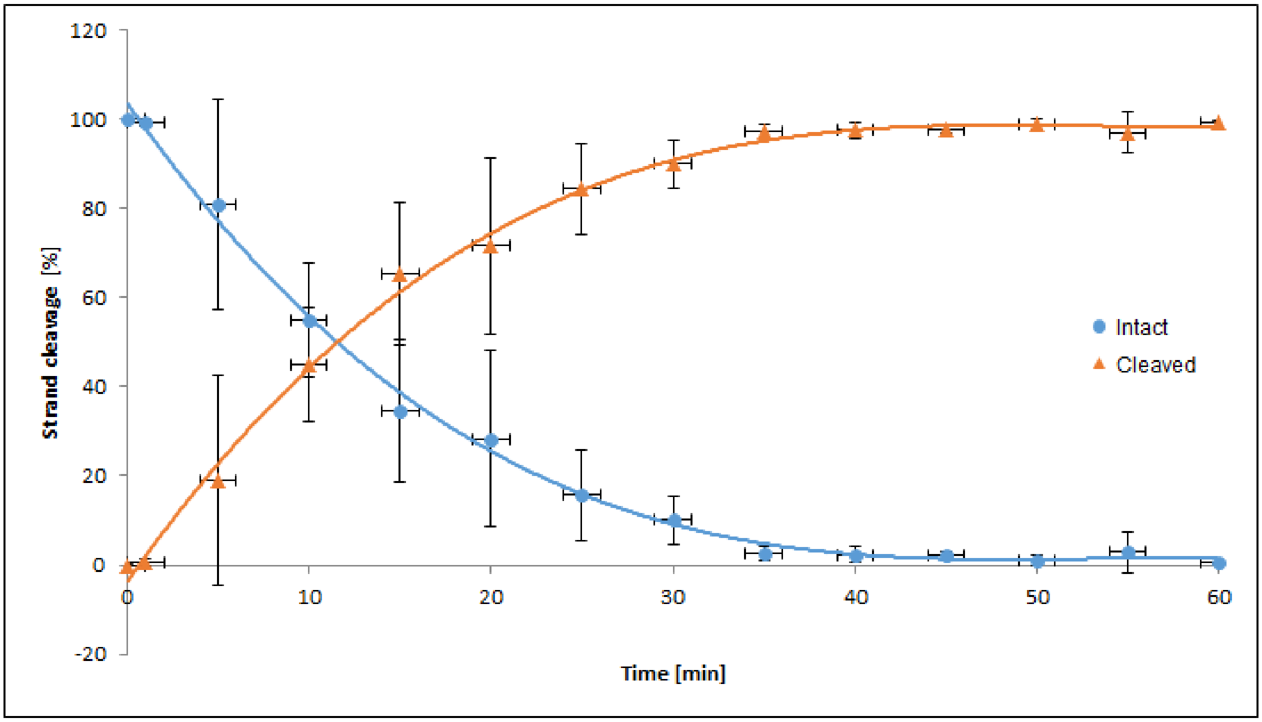

Figure S71. Cleavage assay of dU(-5)RcdA by 0.02U UDG and 0.5U hAPE1. The quantity loss of intact ssDNA (blue) and the quantity increase of SSB-DNA (orange). Error bars are shown.

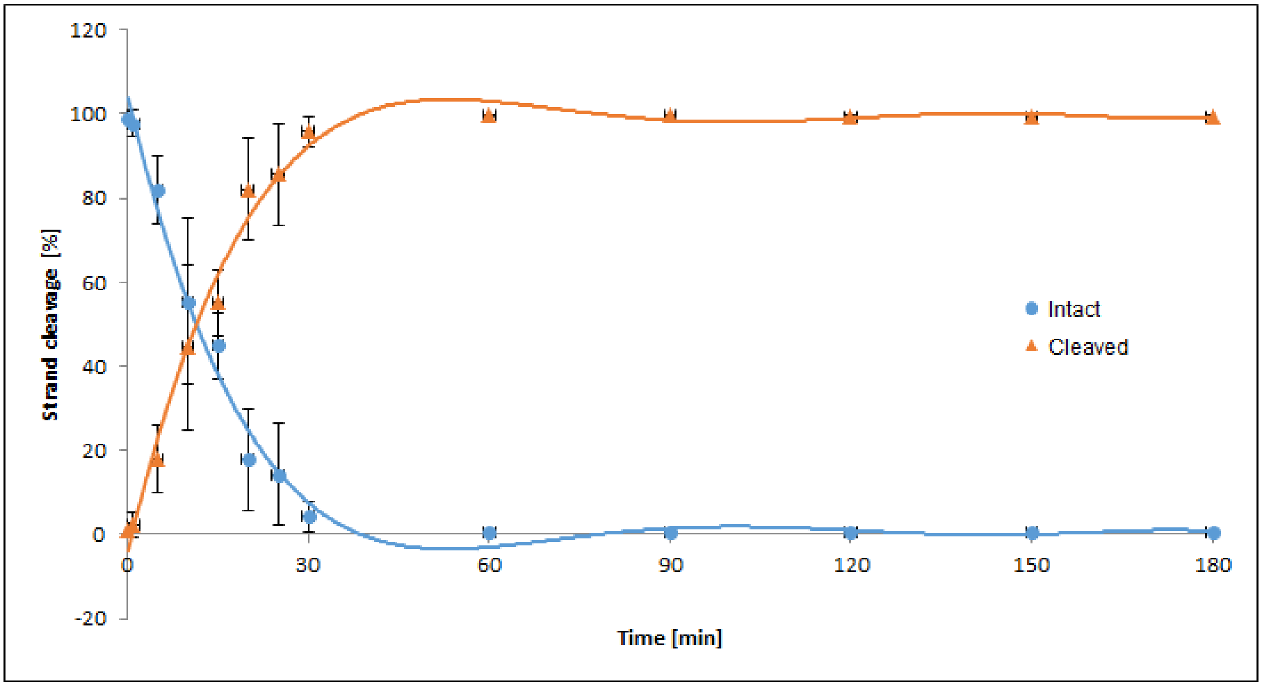

Figure S72. Cleavage assay of dU(-5)RcdA by 0.5U UDG and 0.02U hAPE1. The quantity loss of intact ssDNA (blue) and the quantity increase of SSB-DNA (orange). Error bars are shown.

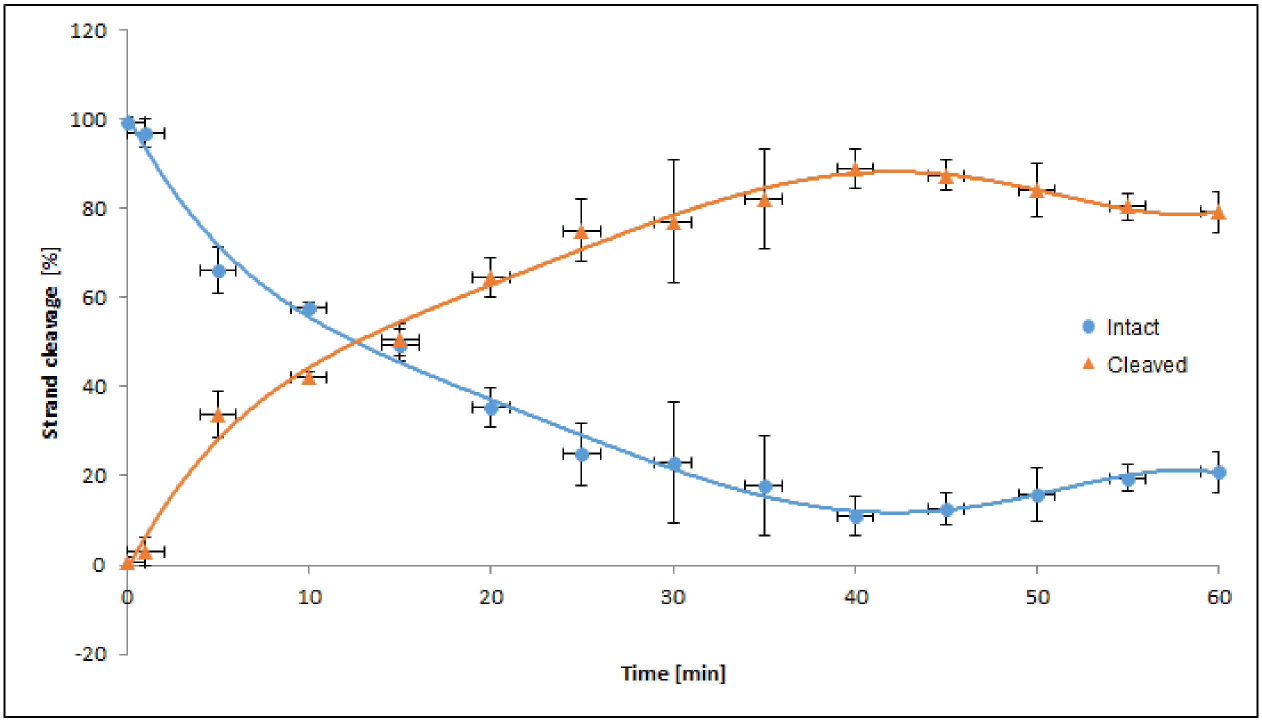

Figure S73. Cleavage assay of dU(+5)RcdA by 0.02U UDG and 0.5U hAPE1. The quantity loss of intact ssDNA (blue) and the quantity increase of SSB-DNA (orange). Error bars are shown.

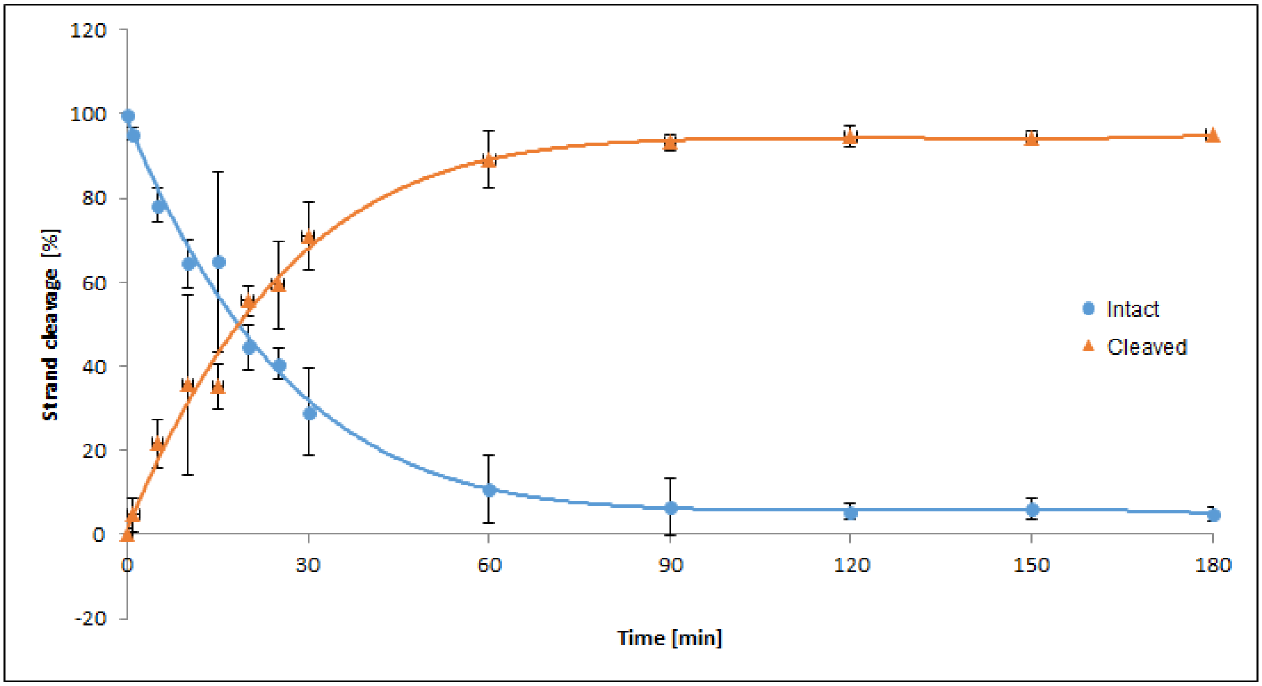

Figure S74. Cleavage assay of dU(+5)RcdA by 0.5U UDG and 0.02U hAPE1. The quantity loss of intact ssDNA (blue) and the quantity increase of SSB-DNA (orange). Error bars are shown.

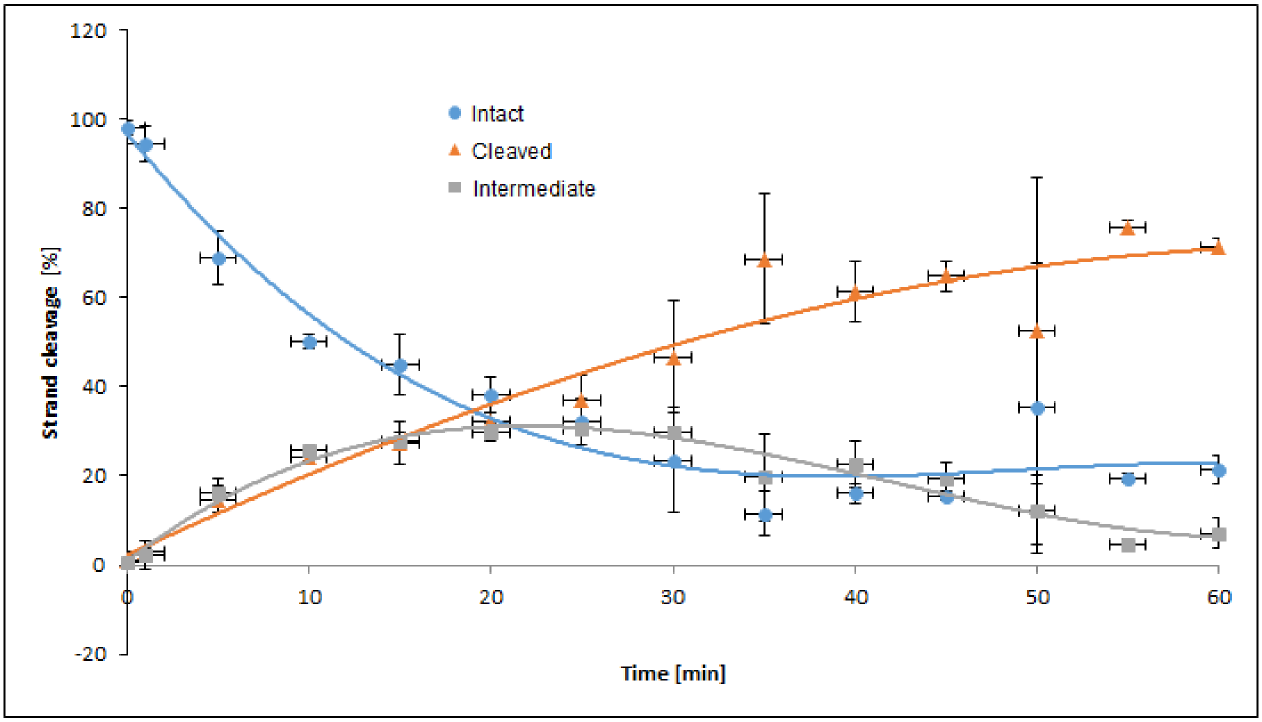

Figure S75. Cleavage assay of dU(-5)(+5)RcdA by 0.02U UDG and 0.5U hAPE1. The quantity loss of intact ssDNA (blue), the quantity increase of SSB-DNA (orange) and an intermediate oligo fragment (grey). Error bars are shown.

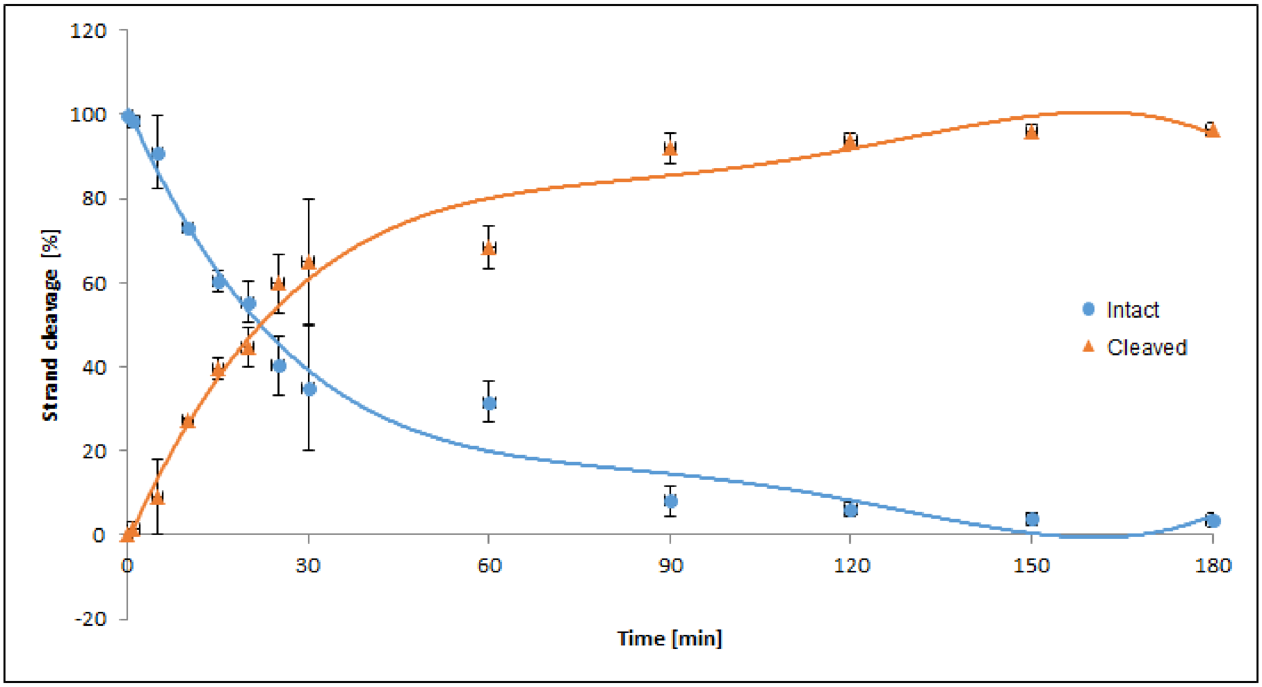

Figure S76. Cleavage assay of dU(-5)(+5)RcdA by 0.5U UDG and 0.02U hAPE1. The quantity loss of intact ssDNA (blue) and the quantity increase of SSB-DNA (orange). Error bars are shown.

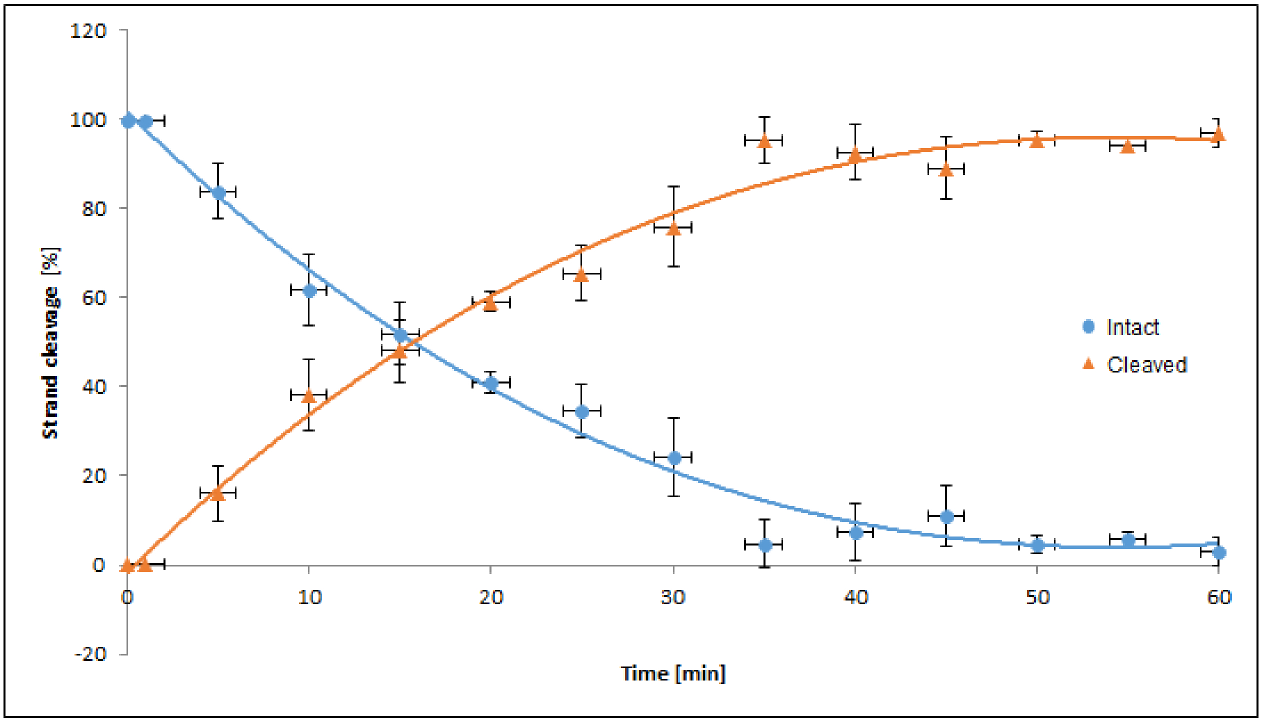

Figure S77. Cleavage assay of dU(-5)ScdG by 0.02U UDG and 0.5U hAPE1. The quantity loss of intact ssDNA (blue) and the quantity increase of SSB-DNA (orange). Error bars are shown.

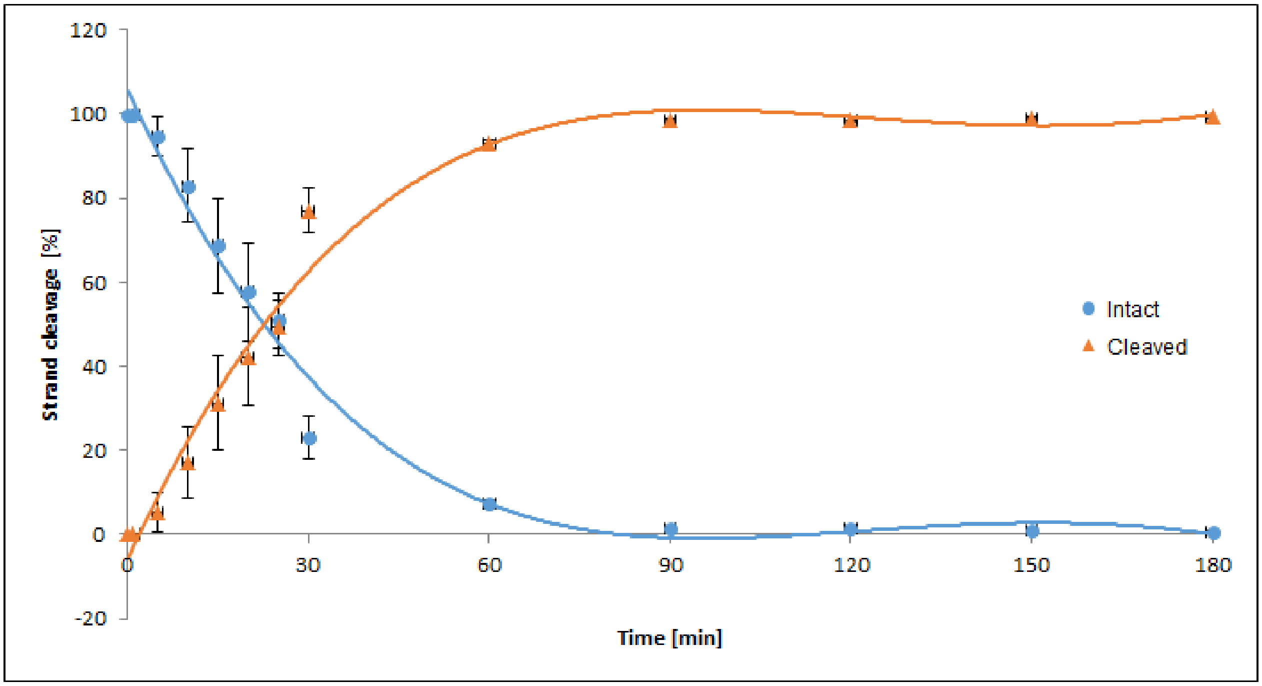

Figure S78. Cleavage assay of dU(-5)ScdG by 0.5U UDG and 0.02U hAPE1. The quantity loss of intact ssDNA (blue) and the quantity increase of SSB-DNA (orange). Error bars are shown.

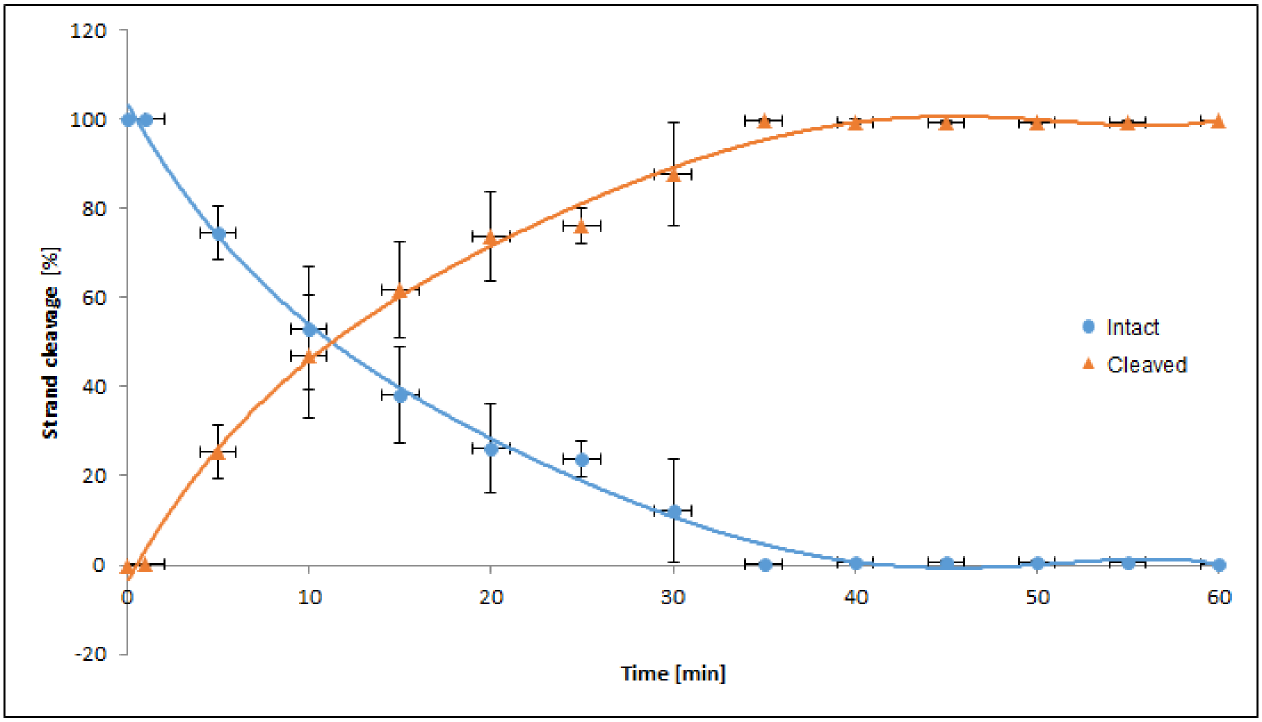

Figure S79. Cleavage assay of dU(+5)ScdG by 0.02U UDG and 0.5U hAPE1. The quantity loss of intact ssDNA (blue) and the quantity increase of SSB-DNA (orange). Error bars are shown.

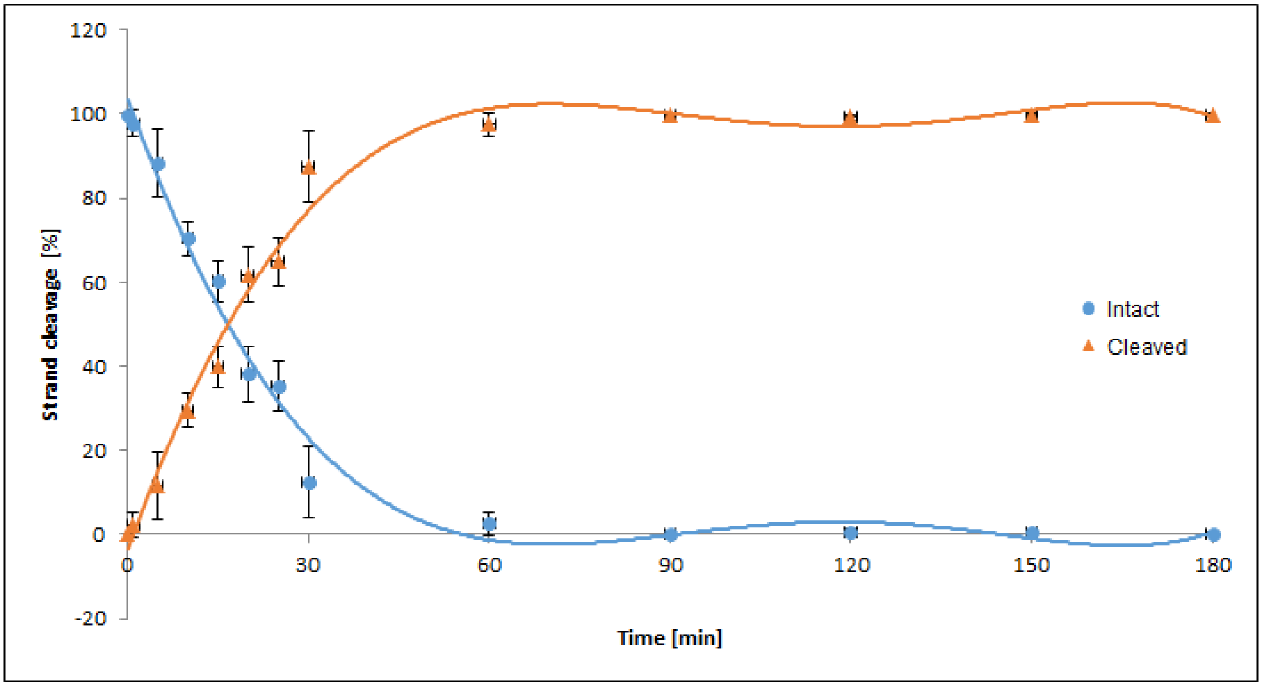

Figure S80. Cleavage assay of dU(+5)ScdG by 0.5U UDG and 0.02U hAPE1. The quantity loss of intact ssDNA (blue) and the quantity increase of SSB-DNA (orange). Error bars are shown.

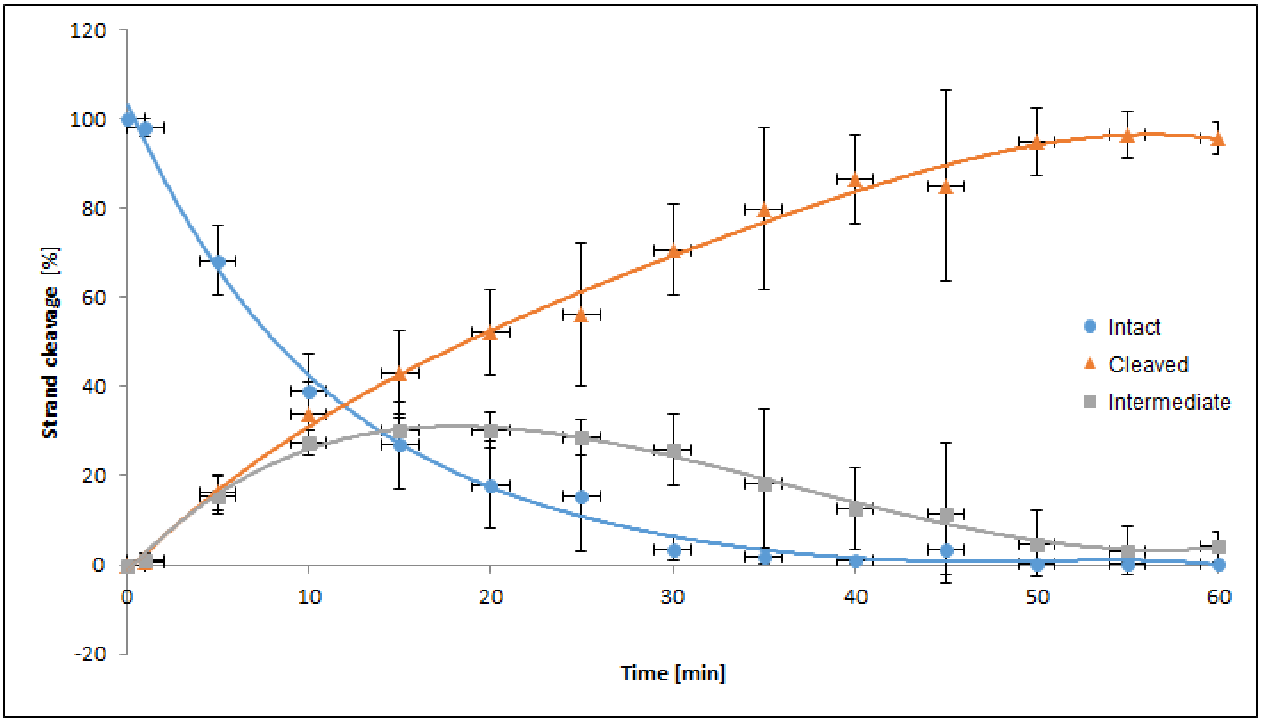

Figure S81. Cleavage assay of dU(-5)(+5)ScdG by 0.02U UDG and 0.5U hAPE1. The quantity loss of intact ssDNA (blue), the quantity increase of SSB-DNA (orange) and an intermediate oligo fragment (grey). Error bars are shown.

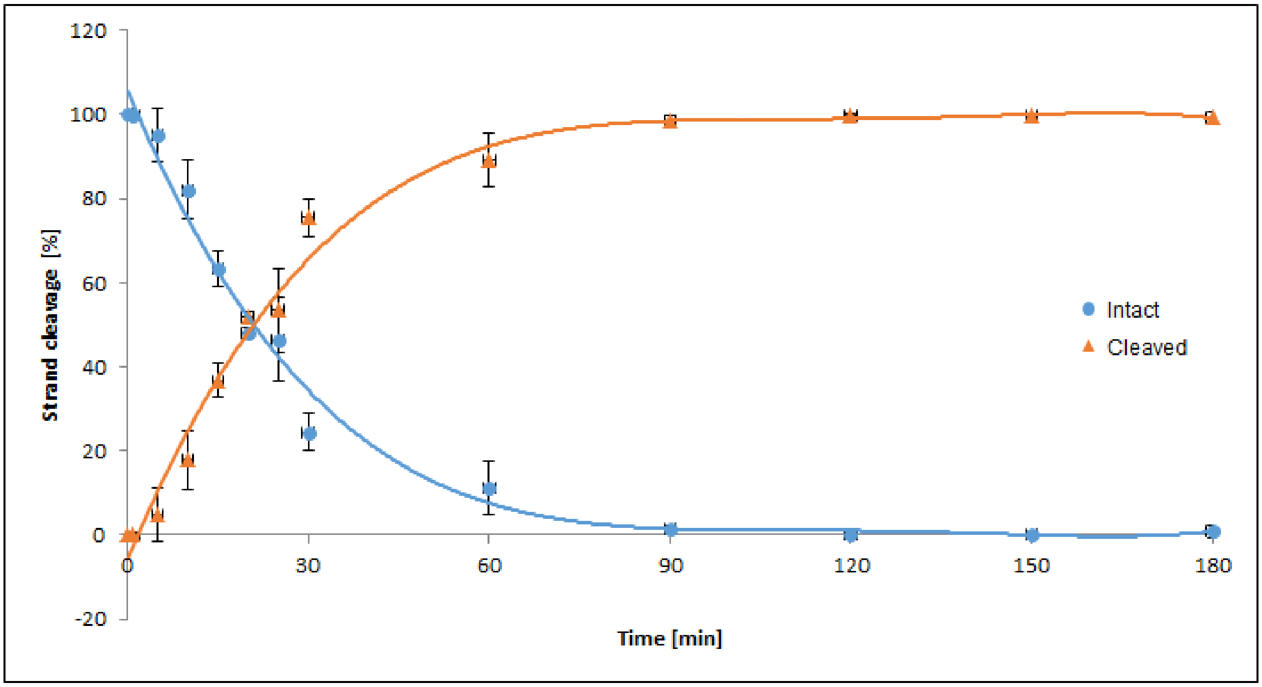

Figure S82. Cleavage assay of dU(-5)(+5)ScdG by 0.5U UDG and 0.02U hAPE1. The quantity loss of intact ssDNA (blue) and the quantity increase of SSB-DNA (orange). Error bars are shown.

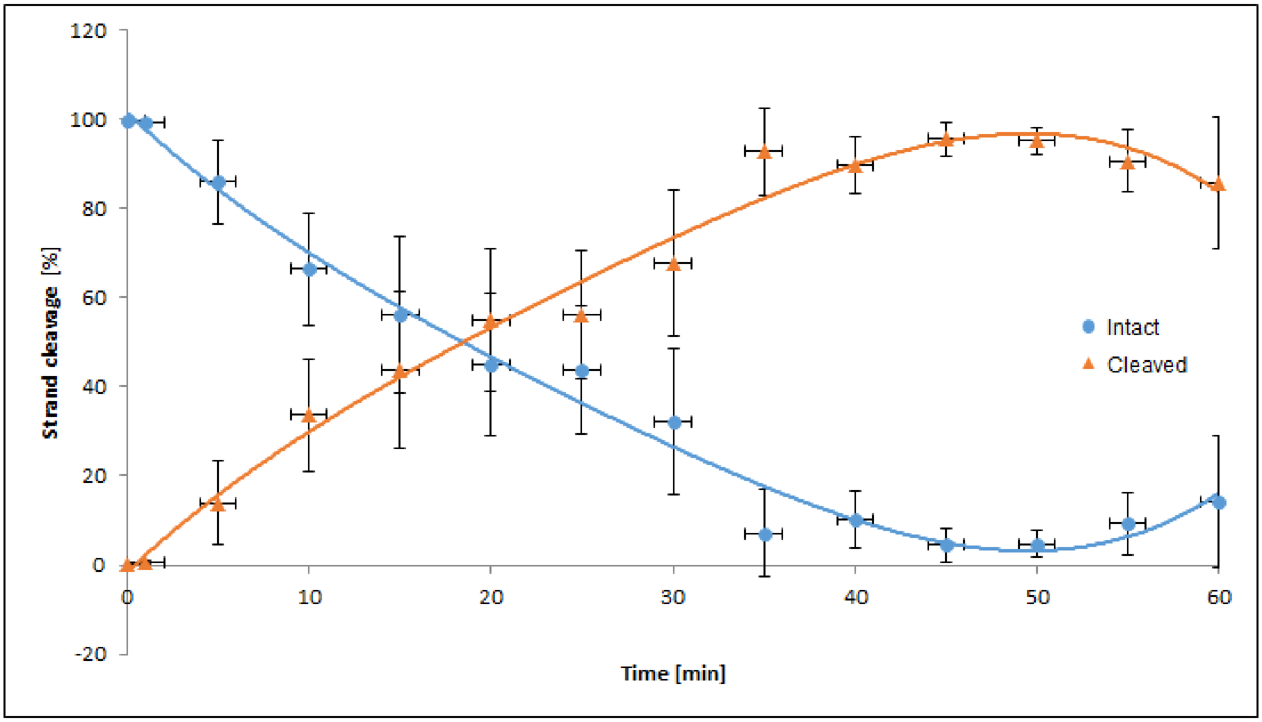

Figure S83. Cleavage assay of dU(-5)RcdG by 0.02U UDG and 0.5U hAPE1. The quantity loss of intact ssDNA (blue) and the quantity increase of SSB-DNA (orange). Error bars are shown.

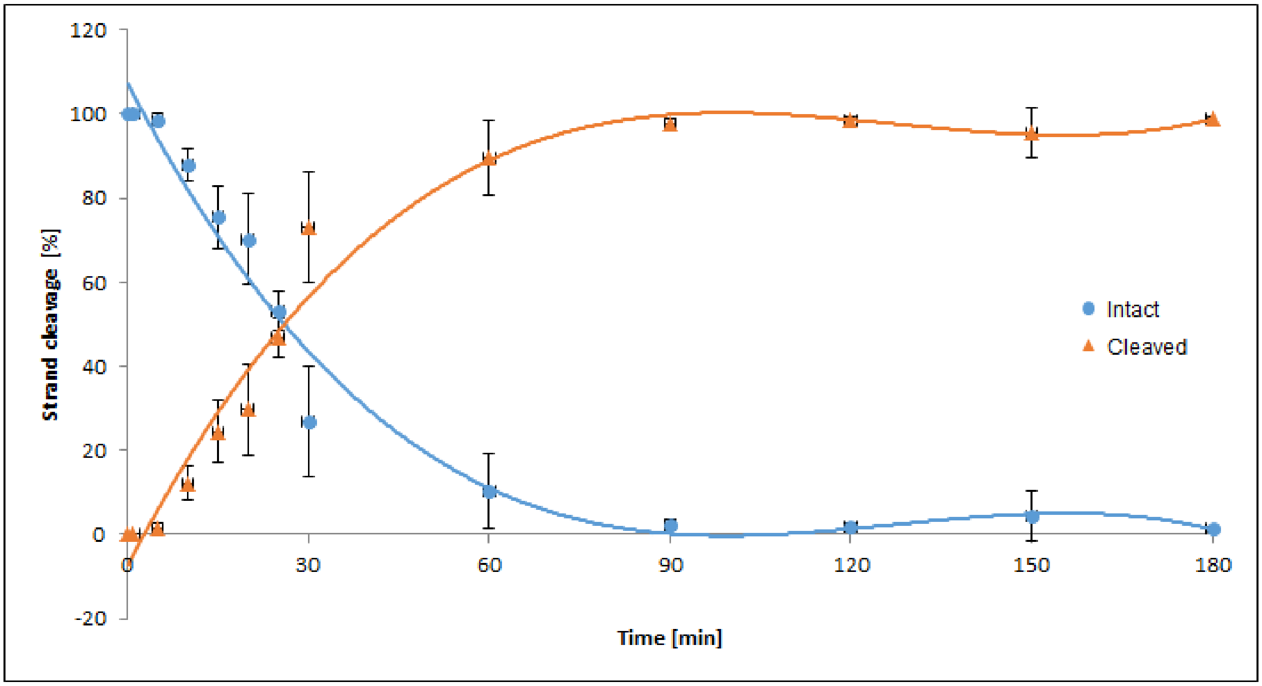

Figure S84. Cleavage assay of dU(-5) RcdG by 0.5U UDG and 0.02U hAPE1. The quantity loss of intact ssDNA (blue) and the quantity increase of SSB-DNA (orange). Error bars are shown.

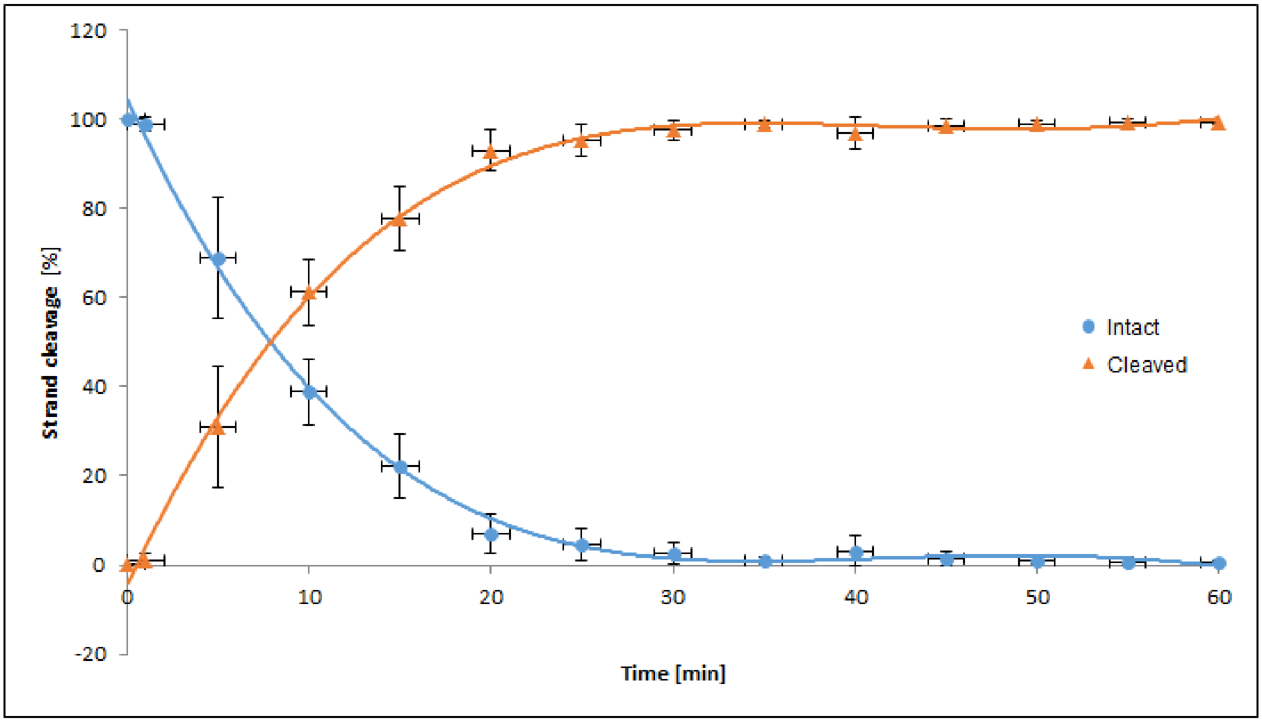

Figure S85. Cleavage assay of dU(+5) RcdG by 0.02U UDG and 0.5U hAPE1. The quantity loss of intact ssDNA (blue) and the quantity increase of SSB-DNA (orange). Error bars are shown.

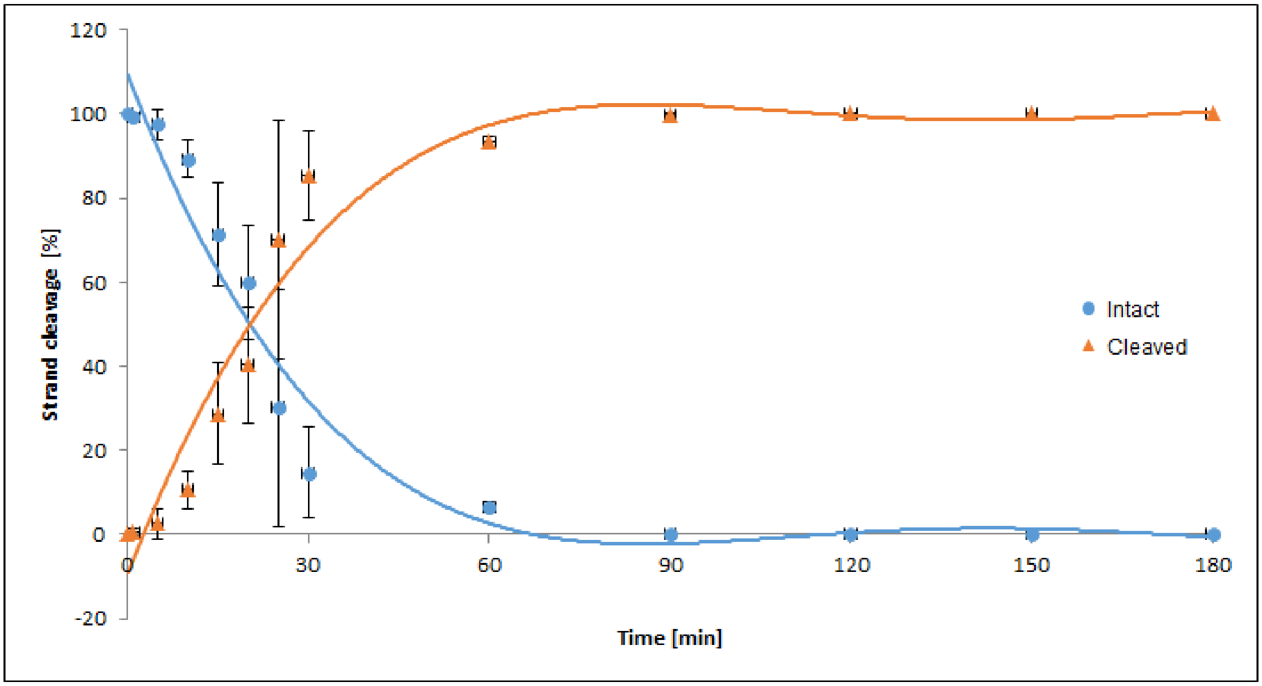

Figure S86. Cleavage assay of dU(+5) RcdG by 0.5U UDG and 0.02U hAPE1. The quantity loss of intact ssDNA (blue) and the quantity increase of SSB-DNA (orange). Error bars are shown.

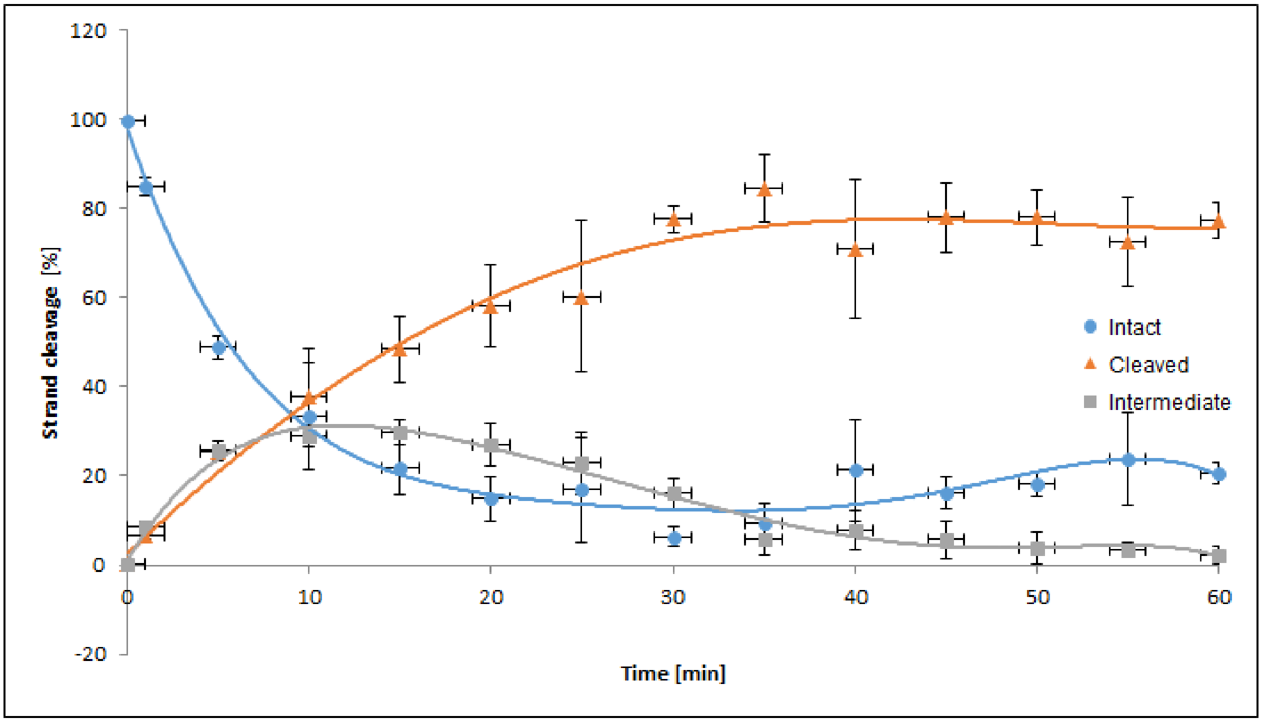

Figure S87. Cleavage assay of dU(-5)(+5) RcdG by 0.02U UDG and 0.5U hAPE1. The quantity loss of intact ssDNA (blue), the quantity increase of SSB-DNA (orange) and an intermediate oligo fragment (grey). Error bars are shown.

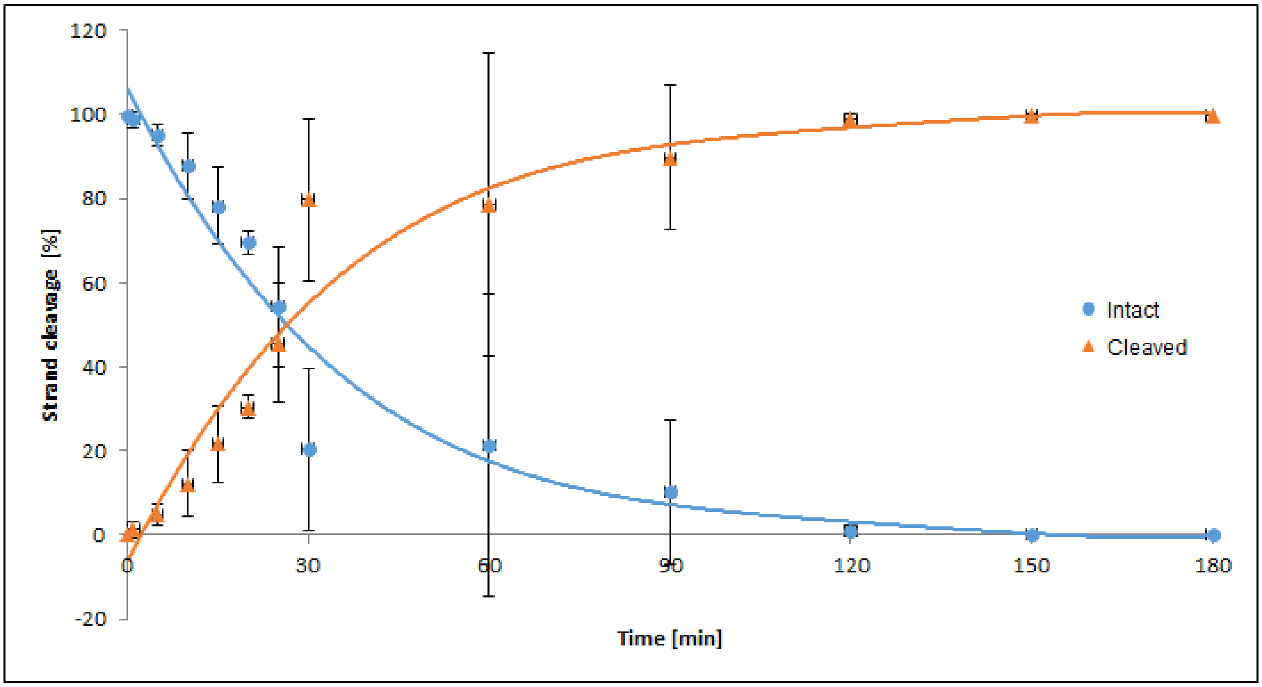

Figure S88. Cleavage assay of dU(-5)(+5) RcdG by 0.5U UDG and 0.02U hAPE1. The quantity loss of intact ssDNA (blue) and the quantity increase of SSB-DNA (orange). Error bars are shown.

Table S2. Raw numerical data of UDG cleavage assays obtained from Quantity One software.

Oligo	Time [min]	Intact oligonucleotide [%]					Cleaved strand [%]					Intermediate fragment [%]				
		1	2	3	Avg	SD	1	2	3	Avg	SD	1	2	3	Avg	SD
Native control oligonucleotides																
dU0	0	100,00	100,00	99,93	99,98	0,04	0,00	0,00	0,07	0,02	0,04					
	1	100,00	99,75	99,69	99,81	0,17	0,00	0,25	0,31	0,19	0,17					
	5	95,16	80,34	74,26	83,25	10,75	4,84	19,66	25,74	16,75	10,75					
	10	77,13	67,90	62,99	69,34	7,18	22,87	32,10	37,01	30,66	7,18					
	15	66,07	53,91	49,78	56,59	8,47	33,93	46,09	50,22	43,41	8,47					
	20	59,68	46,89	42,35	49,64	8,99	40,32	53,11	57,65	50,36	8,99					
	25	60,31	36,45	27,19	41,32	17,09	39,69	63,55	72,81	58,68	17,09					
	30	47,44	28,22	23,99	33,22	12,50	52,56	71,78	76,01	66,78	12,50					
	35	8,89	1,95	1,14	3,99	4,26	91,11	98,05	98,86	96,01	4,26					
	40	1,96	1,11	4,85	2,64	1,96	98,04	98,89	95,15	97,36	1,96					
	45	2,43	1,91	2,91	2,41	0,50	97,57	98,09	97,09	97,59	0,50					
	50	1,77	0,35	1,59	1,24	0,78	98,23	99,65	98,41	98,76	0,78					
	55	5,60	0,00	1,10	2,23	2,97	94,40	100,00	98,90	97,77	2,97					
	60	2,78	0,16	0,81	1,25	1,37	97,22	99,84	99,19	98,75	1,37					
dU(-5) (+5)dA	0	100,00	99,78	99,75	99,84	0,14	0,00	0,22	0,25	0,16	0,14	0,00	0,00	0,00	0,00	0,00
	1	99,21	97,65	95,76	97,54	1,73	0,40	1,17	1,92	1,16	0,76	0,39	1,18	2,32	1,30	0,97
	5	65,08	51,87	64,52	60,49	7,47	11,82	19,59	14,02	15,15	4,01	23,11	28,54	21,46	24,37	3,70
	10	47,80	46,66	34,77	43,08	7,22	21,53	23,94	30,89	25,45	4,86	30,67	29,40	34,34	31,47	2,57
	15	41,20	26,99	15,87	28,02	12,70	25,90	36,84	46,44	36,39	10,28	32,90	36,18	37,70	35,59	2,45
	20	21,33	13,86	2,91	12,70	9,26	40,85	46,75	64,31	50,64	12,20	37,82	39,38	32,78	36,66	3,45
	25	7,08	9,90	2,39	6,46	3,79	54,49	50,38	65,95	56,94	8,07	38,43	39,72	31,66	36,60	4,33
	30	7,16	5,19	1,60	4,65	2,82	54,68	57,38	75,59	62,55	11,37	38,16	37,43	22,81	32,80	8,66
	35	1,84	3,39	1,49	2,24	1,01	94,17	63,76	96,88	84,94	18,39	3,99	32,85	1,64	12,82	17,38
	40	2,03	4,22	0,67	2,31	1,79	89,76	61,27	98,66	83,23	19,53	8,21	34,51	0,67	14,46	17,77
	45	1,73	4,80	1,06	2,53	2,00	89,75	61,32	94,10	81,72	17,80	8,51	33,88	4,84	15,74	15,81
	50	2,65	3,28	0,70	2,21	1,35	85,87	94,44	97,98	92,76	6,22	11,48	2,28	1,33	5,03	5,61
	55	14,17	2,15	0,68	5,67	7,40	46,93	77,23	99,22	74,46	26,25	38,89	20,62	0,10	19,87	19,40
	60	1,02	1,91	0,37	1,10	0,77	92,92	76,24	99,13	89,43	11,84	6,06	21,85	0,50	9,47	11,08
ScdA																
dU(-5)ScdA	0	99,99	99,99	99,77	99,92	0,12	0,01	0,01	0,23	0,08	0,12					
	1	99,96	100,00	99,70	99,88	0,16	0,04	0,00	0,30	0,12	0,16					
	5	99,69	99,91	88,48	96,03	6,54	0,31	0,09	11,52	3,97	6,54					
	10	95,54	82,16	64,86	80,85	15,38	4,46	17,84	35,14	19,15	15,38					
	15	87,94	75,66	57,04	73,54	15,56	12,06	24,34	42,96	26,46	15,56					
	20	70,53	59,95	33,00	54,49	19,35	29,47	40,05	67,00	45,51	19,35					
	25	60,73	41,06	20,77	40,85	19,98	39,27	58,94	79,23	59,15	19,98					
	30	56,82	88,05	24,24	56,37	31,91	43,18	11,95	75,76	43,63	31,91					

	35	3,16	14,25	30,85	16,09	13,94	96,84	85,75	69,15	83,91	13,94					
	40	20,20	25,36	3,67	16,41	11,33	79,80	74,64	96,33	83,59	11,33					
	45	22,61	3,08	1,97	9,22	11,61	77,39	96,92	98,03	90,78	11,61					
	50	14,48	5,66	5,26	8,47	5,21	85,52	94,34	94,74	91,53	5,21					
	55	15,14	6,05	5,41	8,87	5,44	84,86	93,95	94,59	91,13	5,44					
	60	11,03	14,64	4,79	10,16	4,99	88,97	85,36	95,21	89,84	4,99					
dU(+5)ScdA	0	99,53	99,84	100,00	99,79	0,24	0,47	0,16	0,00	0,21	0,24					
	1	97,73	98,36	99,04	98,37	0,66	2,27	1,64	0,96	1,63	0,66					
	5	47,91	44,18	24,11	38,73	12,80	52,09	55,82	75,89	61,27	12,80					
	10	27,41	25,20	13,99	22,20	7,19	72,59	74,80	86,01	77,80	7,19					
	15	15,83	8,11	3,97	9,30	6,02	84,17	91,89	96,03	90,70	6,02					
	20	7,72	7,11	3,69	6,17	2,17	92,28	92,89	96,31	93,83	2,17					
	25	8,46	4,89	2,50	5,28	3,00	91,54	95,11	97,50	94,72	3,00					
	30	5,33	2,83	11,76	6,64	4,61	94,67	97,17	88,24	93,36	4,61					
	35	4,06	1,98	2,30	2,78	1,12	95,94	98,02	97,70	97,22	1,12					
	40	4,00	1,89	1,86	2,58	1,23	96,00	98,11	98,14	97,42	1,23					
	45	3,16	2,00	2,53	2,56	0,58	96,84	98,00	97,47	97,44	0,58					
	50	3,10	2,12	2,39	2,54	0,51	96,90	97,88	97,61	97,46	0,51					
	55	3,29	1,59	2,30	2,39	0,86	96,71	98,41	97,70	97,61	0,86					
	60	3,95	1,82	1,78	2,52	1,24	96,05	98,18	98,22	97,48	1,24					
dU(-5) (+5)ScdA	0	99,88	99,35	99,69	99,64	0,27	0,10	0,00	0,18	0,10	0,09	0,02	0,65	0,13	0,26	0,34
	1	96,43	94,16	92,95	94,51	1,77	1,62	1,70	2,81	2,04	0,66	1,95	4,14	4,25	3,44	1,30
	5	44,86	32,84	33,11	36,93	6,86	27,72	32,83	29,42	29,99	2,60	27,42	34,34	37,47	33,08	5,14
	10	35,54	30,60	33,36	33,17	2,47	34,32	34,69	29,55	32,85	2,87	30,14	34,71	37,09	33,98	3,53
	15	27,50	12,45	7,33	15,76	10,48	39,94	45,31	58,95	48,07	9,80	32,56	42,24	33,72	36,17	5,29
	20	5,90	4,75	5,47	5,38	0,58	60,58	58,38	60,13	59,70	1,16	33,52	36,87	34,40	34,93	1,74
	25	4,81	3,37	3,21	3,80	0,88	62,26	64,55	69,85	65,56	3,89	32,93	32,08	26,94	30,65	3,24
	30	4,10	2,11	1,66	2,63	1,30	67,78	79,07	76,32	74,39	5,89	28,12	18,82	22,02	22,98	4,73
	35	1,61	2,04	1,23	1,63	0,40	97,45	95,79	97,77	97,00	1,06	0,94	2,17	0,99	1,36	0,69
	40	1,43	1,79	1,08	1,43	0,35	97,93	88,60	98,24	94,92	5,48	0,63	9,62	0,67	3,64	5,17
	45	1,62	1,19	0,95	1,26	0,34	94,99	95,51	98,23	96,24	1,74	3,39	3,30	0,82	2,50	1,46
	50	2,32	1,10	0,97	1,46	0,75	92,31	97,55	98,11	95,99	3,20	5,37	1,35	0,92	2,54	2,45
	55	11,07	1,42	0,97	4,49	5,70	73,60	96,71	97,02	89,11	13,43	15,33	1,87	2,02	6,40	7,73
	60	1,75	0,86	0,58	1,06	0,61	97,31	97,68	98,47	97,82	0,59	0,94	1,47	0,95	1,12	0,30
RcdA																
dU(-5)RcdA	0	100,00	100,00	99,97	99,99	0,02	0,00	0,00	0,03	0,01	0,02					
	1	99,93	99,91	98,58	99,47	0,77	0,07	0,09	1,42	0,53	0,77					
	5	93,37	95,63	53,90	80,97	23,47	6,63	4,37	46,10	19,03	23,47					
	10	63,87	60,52	40,45	54,95	12,67	36,13	39,48	59,55	45,05	12,67					
	15	45,57	42,05	16,38	34,67	15,93	54,43	57,95	83,62	65,33	15,93					
	20	45,60	32,78	6,76	28,38	19,79	54,40	67,22	93,24	71,62	19,79					

	25	15,70	25,84	5,33	15,62	10,26	84,30	74,16	94,67	84,38	10,26					
	30	7,32	16,19	6,60	10,04	5,34	92,68	83,81	93,40	89,96	5,34					
	35	4,37	2,76	1,09	2,74	1,64	95,63	97,24	98,91	97,26	1,64					
	40	4,31	2,10	0,75	2,39	1,80	95,69	97,90	99,25	97,61	1,80					
	45	3,19	1,48	1,60	2,09	0,95	96,81	98,52	98,40	97,91	0,95					
	50	0,66	0,20	2,23	1,03	1,06	99,34	99,80	97,77	98,97	1,06					
	55	8,25	0,23	0,30	2,93	4,61	91,75	99,77	99,70	97,07	4,61					
	60	0,40	0,54	1,40	0,78	0,54	99,60	99,46	98,60	99,22	0,54					
dU(+5)RcdA	0	98,00	100,00	99,96	99,32	1,14	2,00	0,00	0,04	0,68	1,14					
	1	93,74	100,00	97,15	96,96	3,13	6,26	0,00	2,85	3,04	3,13					
	5	60,28	69,96	68,17	66,14	5,15	39,72	30,04	31,83	33,86	5,15					
	10	58,21	56,61	58,63	57,82	1,07	41,79	43,39	41,37	42,18	1,07					
	15	47,38	53,60	47,52	49,50	3,55	52,62	46,40	52,48	50,50	3,55					
	20	37,08	38,67	30,48	35,41	4,34	62,92	61,33	69,52	64,59	4,34					
	25	29,29	16,94	28,51	24,91	6,92	70,71	83,06	71,49	75,09	6,92					
	30	31,07	30,72	7,10	22,96	13,74	68,93	69,28	92,90	77,04	13,74					
	35	18,54	28,67	6,11	17,78	11,30	81,46	71,33	93,89	82,22	11,30					
	40	15,70	9,85	7,17	10,91	4,36	84,30	90,15	92,83	89,09	4,36					
	45	16,57	10,67	10,43	12,56	3,48	83,43	89,33	89,57	87,44	3,48					
	50	16,43	21,69	9,58	15,90	6,07	83,57	78,31	90,42	84,10	6,07					
	55	19,99	22,33	16,44	19,59	2,97	80,01	77,67	83,56	80,41	2,97					
	60	17,38	26,08	19,06	20,84	4,61	82,62	73,92	80,94	79,16	4,61					
dU(-5) (+5)RcdA	0	96,30	98,74	99,39	98,14	1,63	1,32	1,26	0,61	1,06	0,40	2,39	0,00	0,00	0,80	1,38
	1	90,05	96,04	97,61	94,57	3,99	3,95	3,18	2,39	3,17	0,78	6,00	0,77	0,00	2,26	3,26
	5	62,61	69,34	74,71	68,89	6,06	17,39	15,42	11,34	14,72	3,09	19,99	15,25	13,95	16,40	3,18
	10	51,42	50,37	48,27	50,02	1,60	23,19	24,24	25,08	24,17	0,95	25,38	25,39	26,65	25,81	0,73
	15	46,64	37,28	50,58	44,83	6,83	25,81	32,79	23,43	27,34	4,86	27,55	29,93	25,99	27,82	1,99
	20	38,20	42,14	34,23	38,19	3,96	31,09	30,18	34,70	31,99	2,39	30,71	27,68	31,07	29,82	1,87
	25	38,02	28,14	30,51	32,22	5,16	31,19	42,33	37,87	37,13	5,61	30,79	29,53	31,62	30,65	1,05
	30	37,01	16,83	16,79	23,55	11,66	32,06	55,08	53,09	46,75	12,75	30,92	28,08	30,12	29,71	1,46
	35	11,81	16,26	6,47	11,51	4,90	64,33	56,96	84,92	68,74	14,49	23,86	26,78	8,61	19,75	9,76
	40	15,19	18,51	14,51	16,07	2,14	59,12	56,14	68,96	61,41	6,71	25,70	25,35	16,52	22,52	5,20
	45	15,30	16,52	14,89	15,57	0,85	61,20	65,60	67,82	64,87	3,37	23,50	17,87	17,29	19,55	3,43
	50	20,33	72,72	12,86	35,30	32,62	59,59	15,05	82,73	52,46	34,39	20,08	12,23	4,42	12,24	7,83
	55	18,47	20,24	19,75	19,48	0,91	77,66	74,81	74,69	75,72	1,68	3,87	4,96	5,56	4,80	0,86
	60	18,91	20,22	24,97	21,36	3,19	70,21	73,77	70,65	71,54	1,94	10,88	6,02	4,38	7,09	3,38
ScdG																
dU(-5)ScdG	0	99,67	100,00	100,00	99,89	0,19	0,33	0,00	0,00	0,11	0,19					
	1	99,62	99,86	99,86	99,78	0,14	0,38	0,14	0,14	0,22	0,14					
	5	90,11	83,76	77,97	83,95	6,07	9,89	16,24	22,03	16,05	6,07					
	10	68,94	63,08	53,22	61,75	7,94	31,06	36,92	46,78	38,25	7,94					

	15	59,41	50,49	45,70	51,87	6,96	40,59	49,51	54,30	48,13	6,96					
	20	43,19	40,98	38,52	40,90	2,33	56,81	59,02	61,48	59,10	2,33					
	25	35,71	39,87	27,87	34,49	6,09	64,29	60,13	72,13	65,51	6,09					
	30	27,86	30,42	13,96	24,08	8,86	72,14	69,58	86,04	75,92	8,86					
	35	10,74	0,85	2,69	4,76	5,26	89,26	99,15	97,31	95,24	5,26					
	40	11,84	0,26	10,09	7,39	6,24	88,16	99,74	89,91	92,61	6,24					
	45	17,18	3,47	12,34	11,00	6,95	82,82	96,53	87,66	89,00	6,95					
	50	6,59	3,02	4,19	4,60	1,82	93,41	96,98	95,81	95,40	1,82					
	55	7,45	5,34	4,59	5,79	1,48	92,55	94,66	95,41	94,21	1,48					
	60	1,84	6,83	0,74	3,14	3,25	98,16	93,17	99,26	96,86	3,25					
dU(+5)ScdG	0	100,00	100,00	100,00	100,00	0,00	0,00	0,00	0,00	0,00	0,00					
	1	100,00	99,81	100,00	99,94	0,11	0,00	0,19	0,00	0,06	0,11					
	5	77,55	78,42	67,68	74,55	5,96	22,45	21,58	32,32	25,45	5,96					
	10	63,30	58,53	37,58	53,14	13,68	36,70	41,47	62,42	46,86	13,68					
	15	49,94	36,41	28,40	38,25	10,88	50,06	63,59	71,60	61,75	10,88					
	20	34,68	28,64	15,19	26,17	9,98	65,32	71,36	84,81	73,83	9,98					
	25	27,97	23,31	19,94	23,74	4,03	72,03	76,69	80,06	76,26	4,03					
	30	24,12	11,10	1,12	12,11	11,54	75,88	88,90	98,88	87,89	11,54					
	35	0,24	0,85	0,08	0,39	0,40	99,76	99,15	99,92	99,61	0,40					
	40	0,41	0,99	0,12	0,51	0,44	99,59	99,01	99,88	99,49	0,44					
	45	0,74	1,05	0,16	0,65	0,45	99,26	98,95	99,84	99,35	0,45					
	50	1,12	0,75	0,25	0,70	0,44	98,88	99,25	99,75	99,30	0,44					
	55	0,36	0,88	0,25	0,50	0,34	99,64	99,12	99,75	99,50	0,34					
	60	0,28	0,43	0,15	0,29	0,14	99,72	99,57	99,85	99,71	0,14					
dU(-5)(+5)ScdG	0	100,00	99,98	100,00	99,99	0,01	0,00	0,02	0,00	0,01	0,01	0,00	0,00	0,00	0,00	0,00
	1	99,99	98,02	96,03	98,02	1,98	0,01	1,04	1,34	0,79	0,70	0,00	0,95	2,63	1,19	1,33
	5	75,09	70,15	59,62	68,29	7,90	12,19	16,35	19,99	16,18	3,90	12,72	13,49	20,38	15,53	4,22
	10	48,10	31,26	37,12	38,82	8,55	27,40	41,64	32,57	33,87	7,21	24,50	27,10	30,32	27,31	2,91
	15	36,61	16,85	27,00	26,82	9,89	35,84	53,74	39,72	43,10	9,42	27,55	29,42	33,28	30,08	2,92
	20	28,65	9,62	15,37	17,88	9,76	43,76	62,52	49,99	52,09	9,55	27,58	27,86	34,64	30,03	4,00
	25	15,57	2,98	27,08	15,21	12,05	55,43	72,70	40,73	56,29	16,01	29,00	24,31	32,19	28,50	3,96
	30	5,18	0,59	4,73	3,50	2,53	69,08	81,65	61,27	70,67	10,28	25,73	17,76	34,00	25,83	8,12
	35	0,19	1,57	3,75	1,84	1,80	99,43	77,03	63,20	79,89	18,28	0,38	21,39	33,05	18,27	16,56
	40	0,77	0,12	1,86	0,92	0,88	89,18	94,83	75,32	86,44	10,04	10,05	5,05	22,82	12,64	9,16
	45	10,05	0,19	0,41	3,55	5,63	60,47	95,08	99,51	85,02	21,38	29,48	4,73	0,08	11,43	15,81
	50	0,51	0,01	0,43	0,32	0,27	86,30	99,01	99,53	94,95	7,49	13,19	0,97	0,04	4,74	7,34
	55	0,12	0,02	0,72	0,29	0,38	90,66	99,98	98,92	96,52	5,10	9,23	0,00	0,36	3,20	5,22
	60	0,29	0,04	0,40	0,24	0,18	93,15	99,79	94,23	95,72	3,56	6,56	0,17	5,37	4,03	3,40
RcdG																
dU(-5)RcdG	0	99,50	99,98	100,00	99,83	0,28	0,50	0,02	0,00	0,17	0,28					
	1	99,30	99,94	99,36	99,53	0,35	0,70	0,06	0,64	0,47	0,35					

	5	92,74	90,06	75,41	86,07	9,33	7,26	9,94	24,59	13,93	9,33					
	10	76,26	70,53	52,30	66,36	12,51	23,74	29,47	47,70	33,64	12,51					
	15	67,65	65,08	35,81	56,18	17,69	32,35	34,92	64,19	43,82	17,69					
	20	58,52	49,25	27,19	44,98	16,10	41,48	50,75	72,81	55,02	16,10					
	25	50,10	54,18	27,26	43,85	14,51	49,90	45,82	72,74	56,15	14,51					
	30	49,96	28,87	17,63	32,15	16,41	50,04	71,13	82,37	67,85	16,41					
	35	2,46	18,35	0,44	7,08	9,81	97,54	81,65	99,56	92,92	9,81					
	40	14,94	12,85	2,81	10,20	6,49	85,06	87,15	97,19	89,80	6,49					
	45	7,44	5,68	0,25	4,46	3,75	92,56	94,32	99,75	95,54	3,75					
	50	5,95	6,94	1,33	4,74	2,99	94,05	93,06	98,67	95,26	2,99					
	55	13,31	13,21	1,28	9,27	6,92	86,69	86,79	98,72	90,73	6,92					
	60	29,95	12,32	0,64	14,31	14,75	70,05	87,68	99,36	85,69	14,75					
dU(+5)RcdG	0	100,00	99,84	100,00	99,95	0,09	0,00	0,16	0,00	0,05	0,09					
	1	99,91	96,95	100,00	98,95	1,73	0,09	3,05	0,00	1,05	1,73					
	5	63,89	58,79	84,52	69,06	13,62	36,11	41,21	15,48	30,94	13,62					
	10	44,58	30,67	41,16	38,80	7,25	55,42	69,33	58,84	61,20	7,25					
	15	28,67	23,51	14,28	22,15	7,29	71,33	76,49	85,72	77,85	7,29					
	20	12,14	5,01	3,72	6,95	4,53	87,86	94,99	96,28	93,05	4,53					
	25	8,46	4,31	1,03	4,60	3,73	91,54	95,69	98,97	95,40	3,73					
	30	4,98	2,10	0,31	2,46	2,35	95,02	97,90	99,69	97,54	2,35					
	35	0,94	1,99	0,19	1,04	0,90	99,06	98,01	99,81	98,96	0,90					
	40	7,00	2,49	0,02	3,17	3,54	93,00	97,51	99,98	96,83	3,54					
	45	3,22	1,14	0,01	1,46	1,63	96,78	98,86	99,99	98,54	1,63					
	50	1,42	1,54	0,03	0,99	0,84	98,58	98,46	99,97	99,01	0,84					
	55	0,40	1,87	0,10	0,79	0,95	99,60	98,13	99,90	99,21	0,95					
	60	0,38	1,20	0,01	0,53	0,61	99,62	98,80	99,99	99,47	0,61					
dU(-5)(+5)RcdG	0	99,71	99,79	99,86	99,79	0,08	0,12	0,00	0,03	0,05	0,07	0,16	0,21	0,11	0,16	0,05
	1	85,07	82,78	86,66	84,84	1,95	6,12	7,44	5,93	6,49	0,82	8,82	9,78	7,41	8,67	1,19
	5	51,45	46,45	48,63	48,84	2,51	22,95	26,88	26,86	25,56	2,27	25,60	26,67	24,51	25,59	1,08
	10	46,45	30,40	22,90	33,25	12,03	26,97	38,34	48,29	37,87	10,67	26,58	31,26	28,81	28,88	2,34
	15	28,93	17,64	18,96	21,84	6,17	39,92	50,62	54,59	48,38	7,59	31,15	31,74	26,45	29,78	2,90
	20	19,99	10,09	14,55	14,87	4,96	47,82	64,54	62,52	58,29	9,13	32,19	25,37	22,93	26,83	4,80
	25	17,60	4,64	28,39	16,87	11,89	52,92	79,70	48,10	60,24	17,02	29,48	15,67	23,51	22,88	6,93
	30	3,98	8,59	6,57	6,38	2,31	77,03	74,69	80,98	77,57	3,18	18,99	16,71	12,45	16,05	3,32
	35	14,14	5,83	8,26	9,41	4,28	77,05	92,26	84,83	84,71	7,60	8,80	1,91	6,91	5,88	3,56
	40	32,14	9,71	21,77	21,21	11,22	56,58	87,56	69,15	71,10	15,58	11,28	2,73	9,08	7,70	4,44
	45	18,96	12,18	17,79	16,31	3,63	72,47	86,88	74,65	78,00	7,77	8,57	0,94	7,56	5,69	4,14
	50	21,46	17,18	15,97	18,20	2,88	70,88	82,18	81,11	78,06	6,24	7,66	0,64	2,91	3,74	3,58
	55	24,19	34,08	13,04	23,77	10,53	73,71	62,25	82,16	72,71	9,99	2,10	3,67	4,80	3,52	1,36
	60	19,73	23,10	18,49	20,44	2,38	80,27	72,66	79,10	77,34	4,10	0,00	4,25	2,40	2,22	2,13

Table S3. Raw numerical data of hAPE1 cleavage assays obtained from Quantity One software.

Oligo	Time [min]	1	2	3	Avg	SD	1	2	3	Avg	SD
		Intact oligonucleotide [%]					Cleaved strand [%]				
Native control oligonucleotides											
dU0	0	99,29	96,84	100,00	98,71	1,66	0,71	3,16	0,00	1,29	1,66
	1	100,00	98,74	100,00	99,58	0,73	0,00	1,26	0,00	0,42	0,73
	5	99,40	94,82	100,00	98,07	2,83	0,60	5,18	0,00	1,93	2,83
	10	98,97	93,65	99,88	97,50	3,37	1,03	6,35	0,12	2,50	3,37
	15	97,98	91,36	99,54	96,29	4,35	2,02	8,64	0,46	3,71	4,35
	20	95,94	87,18	98,85	93,99	6,07	4,06	12,82	1,15	6,01	6,07
	25	92,19	84,92	97,08	91,40	6,12	7,81	15,08	2,92	8,60	6,12
	30	79,20	61,86	70,79	70,62	8,67	20,80	38,14	29,21	29,38	8,67
	60	57,27	36,75	53,17	49,06	10,86	42,73	63,25	46,83	50,94	10,86
	90	33,05	27,38	36,75	32,39	4,72	66,95	72,62	63,25	67,61	4,72
	120	12,49	3,99	0,56	5,68	6,14	87,51	96,01	99,44	94,32	6,14
	150	5,95	0,00	0,32	2,09	3,35	94,05	100,00	99,68	97,91	3,35
	180	7,23	0,00	0,10	2,44	4,15	92,77	100,00	99,90	97,56	4,15
dU(-5) (+5)dA	0	99,93	99,99	99,80	99,91	0,10	0,07	0,01	0,20	0,09	0,10
	1	100,00	100,00	99,92	99,97	0,04	0,00	0,00	0,08	0,03	0,04
	5	99,84	97,84	96,09	97,92	1,87	0,16	2,16	3,91	2,08	1,87
	10	83,76	90,22	87,68	87,22	3,25	16,24	9,78	12,32	12,78	3,25
	15	73,53	77,18	73,06	74,59	2,26	26,47	22,82	26,94	25,41	2,26
	20	59,71	60,79	63,61	61,37	2,02	40,29	39,21	36,39	38,63	2,02
	25	55,73	50,66	64,42	56,94	6,96	44,27	49,34	35,58	43,06	6,96
	30	34,06	26,12	36,18	32,12	5,30	65,94	73,88	63,82	67,88	5,30
	60	8,14	6,86	10,33	8,44	1,75	91,86	93,14	89,67	91,56	1,75
	90	0,18	1,45	0,00	0,54	0,79	99,82	98,55	100,00	99,46	0,79
	120	0,02	2,71	0,00	0,91	1,56	99,98	97,29	100,00	99,09	1,56
	150	0,36	1,20	0,00	0,52	0,62	99,64	98,80	100,00	99,48	0,62
	180	0,06	0,67	0,00	0,24	0,37	99,94	99,33	100,00	99,76	0,37
ScdA											
dU(-5)ScdA	0	99,54	99,81	99,79	99,71	0,15	0,46	0,19	0,21	0,29	0,15
	1	99,97	99,64	99,76	99,79	0,17	0,03	0,36	0,24	0,21	0,17
	5	98,01	98,31	99,15	98,49	0,59	1,99	1,69	0,85	1,51	0,59
	10	96,38	96,36	98,04	96,93	0,96	3,62	3,64	1,96	3,07	0,96
	15	93,24	91,40	96,01	93,55	2,32	6,76	8,60	3,99	6,45	2,32
	20	78,57	82,29	92,32	84,39	7,11	21,43	17,71	7,68	15,61	7,11
	25	79,68	79,18	87,31	82,06	4,56	20,32	20,82	12,69	17,94	4,56
	30	14,22	56,13	80,56	50,30	33,55	85,78	43,87	19,44	49,70	33,55
	60	13,09	6,79	9,32	9,73	3,17	86,91	93,21	90,68	90,27	3,17
	90	2,01	2,88	4,83	3,24	1,44	97,99	97,12	95,17	96,76	1,44

	120	1,41	0,78	0,63	0,94	0,41	98,59	99,22	99,37	99,06	0,41
	150	0,71	0,90	2,06	1,22	0,73	99,29	99,10	97,94	98,78	0,73
	180	0,67	0,67	0,42	0,59	0,15	99,33	99,33	99,58	99,41	0,15
dU(+5)ScdA	0	100,00	100,00	99,84	99,95	0,09	0,00	0,00	0,16	0,05	0,09
	1	99,89	97,76	98,85	98,83	1,07	0,11	2,24	1,15	1,17	1,07
	5	88,41	88,56	90,93	89,30	1,41	11,59	11,44	9,07	10,70	1,41
	10	75,35	65,81	70,51	70,56	4,77	24,65	34,19	29,49	29,44	4,77
	15	57,09	51,77	51,38	53,41	3,18	42,91	48,23	48,62	46,59	3,18
	20	52,08	41,53	43,19	45,60	5,67	47,92	58,47	56,81	54,40	5,67
	25	40,78	29,27	38,55	36,20	6,10	59,22	70,73	61,45	63,80	6,10
	30	38,40	25,29	29,24	30,97	6,72	61,60	74,71	70,76	69,03	6,72
	60	11,34	13,75	8,50	11,20	2,63	88,66	86,25	91,50	88,80	2,63
	90	2,96	3,24	4,68	3,63	0,92	97,04	96,76	95,32	96,37	0,92
	120	2,69	3,43	3,46	3,19	0,44	97,31	96,57	96,54	96,81	0,44
	150	2,14	2,82	3,46	2,81	0,66	97,86	97,18	96,54	97,19	0,66
	180	1,91	3,57	13,00	6,16	5,98	98,09	96,43	87,00	93,84	5,98
dU(-5)(+5)ScdA	0	100,00	99,48	99,49	99,66	0,30	0,00	0,52	0,51	0,34	0,30
	1	99,92	99,24	99,66	99,60	0,34	0,08	0,76	0,34	0,40	0,34
	5	89,17	71,69	96,49	85,78	12,74	10,83	28,31	3,51	14,22	12,74
	10	71,05	46,53	90,37	69,32	21,97	28,95	53,47	9,63	30,68	21,97
	15	56,28	28,22	60,58	48,36	17,57	43,72	71,78	39,42	51,64	17,57
	20	35,53	18,09	60,63	38,09	21,39	64,47	81,91	39,37	61,91	21,39
	25	31,34	12,25	35,22	26,27	12,29	68,66	87,75	64,78	73,73	12,29
	30	42,70	2,66	10,04	18,47	21,31	57,30	97,34	89,96	81,53	21,31
	60	9,32	1,89	3,23	4,81	3,96	90,68	98,11	96,77	95,19	3,96
	90	3,67	1,12	1,88	2,22	1,31	96,33	98,88	98,12	97,78	1,31
	120	2,38	0,62	1,72	1,57	0,89	97,62	99,38	98,28	98,43	0,89
	150	1,73	1,31	1,19	1,41	0,29	98,27	98,69	98,81	98,59	0,29
	180	0,87	0,48	0,87	0,74	0,23	99,13	99,52	99,13	99,26	0,23
RcdA											
dU(-5)RcdA	0	99,89	99,85	97,23	98,99	1,52	0,11	0,15	2,77	1,01	1,52
	1	99,79	99,34	94,37	97,83	3,01	0,21	0,66	5,63	2,17	3,01
	5	91,32	76,60	78,02	81,98	8,12	8,68	23,40	21,98	18,02	8,12
	10	76,77	51,80	37,80	55,46	19,74	23,23	48,20	62,20	44,54	19,74
	15	50,25	35,84	48,58	44,89	7,88	49,75	64,16	51,42	55,11	7,88
	20	31,83	11,18	10,53	17,85	12,12	68,17	88,82	89,47	82,15	12,12
	25	27,93	10,01	4,91	14,28	12,09	72,07	89,99	95,09	85,72	12,09
	30	8,27	3,20	1,25	4,24	3,63	91,73	96,80	98,75	95,76	3,63
	60	0,10	0,68	0,40	0,39	0,29	99,90	99,32	99,60	99,61	0,29
	90	0,14	0,76	0,36	0,42	0,31	99,86	99,24	99,64	99,58	0,31
	120	0,30	0,90	0,20	0,47	0,38	99,70	99,10	99,80	99,53	0,38

	150	0,33	0,87	0,23	0,48	0,34	99,67	99,13	99,77	99,52	0,34
	180	0,59	1,13	0,05	0,59	0,54	99,41	98,87	99,95	99,41	0,54
dU(+5)RcdA	0	99,98	99,91	99,83	99,90	0,08	0,02	0,09	0,17	0,10	0,08
	1	97,00	94,31	94,40	95,24	1,53	3,00	5,69	5,60	4,76	1,53
	5	81,86	79,20	73,89	78,32	4,06	18,14	20,80	26,11	21,68	4,06
	10	70,97	62,27	60,04	64,43	5,78	29,03	37,73	39,96	35,57	5,78
	15	62,09	87,61	44,78	64,83	21,55	37,91	12,39	55,22	35,17	21,55
	20	50,36	41,04	41,99	44,46	5,13	49,64	58,96	58,01	55,54	5,13
	25	44,15	40,79	36,96	40,63	3,60	55,85	59,21	63,04	59,37	3,60
	30	41,05	22,36	24,16	29,19	10,31	58,95	77,64	75,84	70,81	10,31
	60	19,78	4,25	8,49	10,84	8,03	80,22	95,75	91,51	89,16	8,03
	90	14,51	2,19	3,22	6,64	6,84	85,49	97,81	96,78	93,36	6,84
	120	6,74	3,18	6,28	5,40	1,93	93,26	96,82	93,72	94,60	1,93
	150	5,56	3,56	8,63	5,91	2,56	94,44	96,44	91,37	94,09	2,56
	180	6,51	3,15	5,13	4,93	1,69	93,49	96,85	94,87	95,07	1,69
dU(-5) (+5)RcdA	0	99,94	99,87	100,00	99,94	0,07	0,06	0,13	0,00	0,06	0,07
	1	97,43	97,75	100,00	98,39	1,40	2,57	2,25	0,00	1,61	1,40
	5	90,46	82,48	100,00	90,98	8,77	9,54	17,52	0,00	9,02	8,77
	10	73,49	72,85	72,40	72,92	0,55	26,51	27,15	27,60	27,08	0,55
	15	61,51	62,10	57,42	60,34	2,55	38,49	37,90	42,58	39,66	2,55
	20	59,55	50,29	56,59	55,47	4,73	40,45	49,71	43,41	44,53	4,73
	25	44,39	32,23	44,05	40,22	6,93	55,61	67,77	55,95	59,78	6,93
	30	50,30	20,77	34,06	35,04	14,79	49,70	79,23	65,94	64,96	14,79
	60	37,27	29,28	28,30	31,62	4,92	62,73	70,72	71,70	68,38	4,92
	90	11,30	3,92	8,82	8,02	3,76	88,70	96,08	91,18	91,98	3,76
	120	4,71	6,04	7,93	6,22	1,62	95,29	93,96	92,07	93,78	1,62
	150	4,19	2,36	5,23	3,93	1,45	95,81	97,64	94,77	96,07	1,45
	180	5,42	1,93	3,50	3,62	1,75	94,58	98,07	96,50	96,38	1,75
ScdG											
dU(-5)ScdG	0	99,87	100,00	99,77	99,88	0,11	0,13	0,00	0,23	0,12	0,11
	1	100,00	100,00	99,69	99,90	0,18	0,00	0,00	0,31	0,10	0,18
	5	89,73	98,93	95,28	94,65	4,63	10,27	1,07	4,72	5,35	4,63
	10	74,02	91,22	83,61	82,95	8,62	25,98	8,78	16,39	17,05	8,62
	15	57,17	79,51	69,58	68,75	11,19	42,83	20,49	30,42	31,25	11,19
	20	44,84	67,24	61,06	57,71	11,57	55,16	32,76	38,94	42,29	11,57
	25	44,13	56,96	51,53	50,87	6,44	55,87	43,04	48,47	49,13	6,44
	30	28,53	18,13	22,33	23,00	5,23	71,47	81,87	77,67	77,00	5,23
	60	6,22	6,95	8,31	7,16	1,06	93,78	93,05	91,69	92,84	1,06
	90	1,42	0,98	2,06	1,48	0,54	98,58	99,02	97,94	98,52	0,54
	120	0,84	0,94	2,08	1,29	0,69	99,16	99,06	97,92	98,71	0,69
	150	0,66	1,03	1,40	1,03	0,37	99,34	98,97	98,60	98,97	0,37

	180	0,27	0,59	1,20	0,68	0,47	99,73	99,41	98,80	99,32	0,47
dU(+5)ScdG	0	99,82	100,00	99,97	99,93	0,10	0,18	0,00	0,03	0,07	0,10
	1	94,37	100,00	99,17	97,85	3,04	5,63	0,00	0,83	2,15	3,04
	5	81,24	97,00	86,87	88,37	7,99	18,76	3,00	13,13	11,63	7,99
	10	69,39	74,92	67,07	70,46	4,03	30,61	25,08	32,93	29,54	4,03
	15	54,72	61,49	64,31	60,17	4,93	45,28	38,51	35,69	39,83	4,93
	20	43,52	30,93	40,15	38,20	6,52	56,48	69,07	59,85	61,80	6,52
	25	40,68	28,94	35,90	35,17	5,91	59,32	71,06	64,10	64,83	5,91
	30	22,03	5,98	9,56	12,52	8,42	77,97	94,02	90,44	87,48	8,42
	60	5,41	0,00	2,23	2,55	2,72	94,59	100,00	97,77	97,45	2,72
	90	0,75	0,00	0,33	0,36	0,37	99,25	100,00	99,67	99,64	0,37
	120	0,75	0,00	1,35	0,70	0,68	99,25	100,00	98,65	99,30	0,68
	150	0,90	0,00	0,23	0,38	0,47	99,10	100,00	99,77	99,62	0,47
	180	0,62	0,00	0,29	0,30	0,31	99,38	100,00	99,71	99,70	0,31
dU(-5)(+5)ScdG	0	99,99	100,00	100,00	100,00	0,00	0,01	0,00	0,00	0,00	0,00
	1	99,57	100,00	99,98	99,85	0,24	0,43	0,00	0,02	0,15	0,24
	5	87,73	99,95	97,74	95,14	6,51	12,27	0,05	2,26	4,86	6,51
	10	74,06	86,44	86,05	82,18	7,03	25,94	13,56	13,95	17,82	7,03
	15	60,77	68,02	61,17	63,32	4,07	39,23	31,98	38,83	36,68	4,07
	20	47,50	47,50	49,65	48,21	1,24	52,50	52,50	50,35	51,79	1,24
	25	38,59	57,63	43,18	46,47	9,94	61,41	42,37	56,82	53,53	9,94
	30	29,64	21,44	22,35	24,48	4,49	70,36	78,56	77,65	75,52	4,49
	60	10,72	4,81	17,49	11,01	6,35	89,28	95,19	82,51	88,99	6,35
	90	2,57	0,75	0,77	1,36	1,05	97,43	99,25	99,23	98,64	1,05
	120	0,85	0,01	0,17	0,34	0,44	99,15	99,99	99,83	99,66	0,44
	150	0,59	0,00	0,01	0,20	0,34	99,41	100,00	99,99	99,80	0,34
	180	2,43	0,11	0,00	0,85	1,37	97,57	99,89	100,00	99,15	1,37
RcdG											
dU(-5)RcdG	0	100,00	100,00	100,00	100,00	0,00	0,00	0,00	0,00	0,00	0,00
	1	100,00	100,00	100,00	100,00	0,00	0,00	0,00	0,00	0,00	0,00
	5	99,84	98,93	96,98	98,58	1,46	0,16	1,07	3,02	1,42	1,46
	10	91,84	87,39	84,14	87,79	3,87	8,16	12,61	15,86	12,21	3,87
	15	79,42	80,11	66,97	75,50	7,40	20,58	19,89	33,03	24,50	7,40
	20	79,27	73,43	58,18	70,29	10,89	20,73	26,57	41,82	29,71	10,89
	25	47,85	56,14	55,39	53,13	4,59	52,15	43,86	44,61	46,87	4,59
	30	12,29	30,89	37,77	26,98	13,18	87,71	69,11	62,23	73,02	13,18
	60	1,91	19,81	9,29	10,34	9,00	98,09	80,19	90,71	89,66	9,00
	90	2,34	1,05	3,28	2,22	1,12	97,66	98,95	96,72	97,78	1,12
	120	1,89	0,59	2,61	1,70	1,02	98,11	99,41	97,39	98,30	1,02
	150	1,23	0,58	11,22	4,34	5,96	98,77	99,42	88,78	95,66	5,96
	180	1,43	0,52	1,82	1,26	0,67	98,57	99,48	98,18	98,74	0,67

dU(+5)RcdG	0	100,00	100,00	100,00	100,00	0,00	0,00	0,00	0,00	0,00	0,00
	1	98,38	100,00	100,00	99,46	0,93	1,62	0,00	0,00	0,54	0,93
	5	93,27	99,98	99,18	97,48	3,66	6,73	0,02	0,82	2,52	3,66
	10	84,33	91,38	92,38	89,36	4,39	15,67	8,62	7,62	10,64	4,39
	15	59,69	70,30	83,95	71,31	12,16	40,31	29,70	16,05	28,69	12,16
	20	70,80	44,57	63,97	59,78	13,61	29,20	55,43	36,03	40,22	13,61
	25	0,00	33,83	56,36	30,06	28,37	100,00	66,17	43,64	69,94	28,37
	30	11,31	6,06	26,67	14,68	10,71	88,69	93,94	73,33	85,32	10,71
	60	6,54	5,12	7,70	6,45	1,29	93,46	94,88	92,30	93,55	1,29
	90	0,11	0,00	0,35	0,15	0,18	99,89	100,00	99,65	99,85	0,18
	120	0,01	0,00	0,00	0,00	0,01	99,99	100,00	100,00	100,00	0,01
	150	0,01	0,00	0,00	0,00	0,01	99,99	100,00	100,00	100,00	0,01
	180	0,00	0,00	0,00	0,00	0,00	100,00	100,00	100,00	100,00	0,00
dU(-5)(+5)RcdG	0	100,00	99,96	99,55	99,84	0,25	0,00	0,04	0,45	0,16	0,25
	1	99,90	96,73	99,61	98,75	1,75	0,10	3,27	0,39	1,25	1,75
	5	97,26	92,30	96,16	95,24	2,61	2,74	7,70	3,84	4,76	2,61
	10	82,94	83,41	96,96	87,77	7,97	17,06	16,59	3,04	12,23	7,97
	15	85,43	68,08	81,35	78,29	9,07	14,57	31,92	18,65	21,71	9,07
	20	68,14	68,01	72,83	69,66	2,75	31,86	31,99	27,17	30,34	2,75
	25	39,90	54,89	68,07	54,29	14,10	60,10	45,11	31,93	45,71	14,10
	30	0,72	39,54	20,70	20,32	19,41	99,28	60,46	79,30	79,68	19,41
	60	0,01	62,91	1,19	21,37	35,98	99,99	37,09	98,81	78,63	35,98
	90	0,00	30,08	0,81	10,30	17,14	100,00	69,92	99,19	89,70	17,14
	120	2,41	0,33	0,27	1,01	1,22	97,59	99,67	99,73	98,99	1,22
	150	0,00	0,59	0,04	0,21	0,33	100,00	99,41	99,96	99,79	0,33
	180	0,00	0,21	0,00	0,07	0,12	100,00	99,79	100,00	99,93	0,12

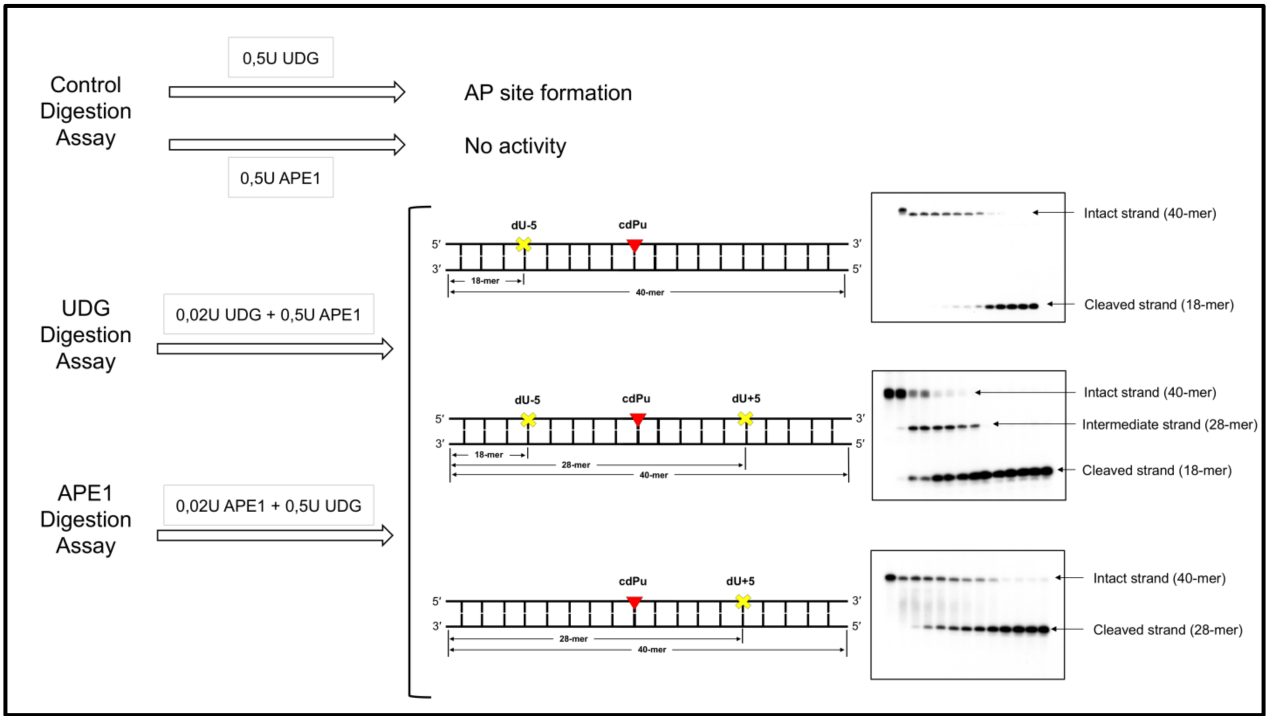

Figure S89. Schematic representation of cleavage assays and control cleavage assays.
